# Does cannabidiol make cannabis safer? A randomised, double-blind, cross-over trial of cannabis with four different CBD:THC ratios

**DOI:** 10.1038/s41386-022-01478-z

**Published:** 2022-11-16

**Authors:** Amir Englund, Dominic Oliver, Edward Chesney, Lucy Chester, Jack Wilson, Simina Sovi, Andrea De Micheli, John Hodsoll, Paolo Fusar-Poli, John Strang, Robin M. Murray, Tom P. Freeman, Philip McGuire

**Affiliations:** 1 National Addiction Centre, Institute of Psychiatry, Psychology and Neuroscience, King’s College London, 4 Windsor Walk, SE5 8AF London, UK; 2 Department of Psychosis Studies, Institute of Psychiatry, Psychology and Neuroscience, King’s College London, 16 De Crespigny Park, SE5 8AF London, UK; 3 The Matilda Centre for Research in Mental Health and Substance Use, The University of Sydney, Level 6, Jane Foss Russell Building, G02, Sydney, 2006 NSW Australia; 4 Department of Biostatistics and Health Informatics, Institute of Psychiatry, Psychology and Neuroscience, King’s College London, 16 De Crespigny Park, SE5 8AF London, UK; 5 Department of Brain and Behavioural Sciences, University of Pavia, Pavia, Italy; 6 South London & Maudsley, NHS Foundation Trust, Maudsley Hospital, London, UK; 7 Department of Psychology, University of Bath, Claverton Down, Bath, BA2 7AY UK

**Keywords:** Experimental models of disease, Translational research, Human behaviour

## Abstract

As countries adopt more permissive cannabis policies, it is increasingly important to identify strategies that can reduce the harmful effects of cannabis use. This study aimed to determine if increasing the CBD content of cannabis can reduce its harmful effects. Forty-six healthy, infrequent cannabis users participated in a double-blind, within-subject, randomised trial of cannabis preparations varying in CBD content. There was an initial baseline visit followed by four drug administration visits, in which participants inhaled vaporised cannabis containing 10 mg THC and either 0 mg (0:1 CBD:THC), 10 mg (1:1), 20 mg (2:1), or 30 mg (3:1) CBD, in a randomised, counter-balanced order. The primary outcome was change in delayed verbal recall on the Hopkins Verbal Learning Task. Secondary outcomes included change in severity of psychotic symptoms (e.g., Positive and Negative Syndrome Scale [PANSS] positive subscale), plus further cognitive, subjective, pleasurable, pharmacological and physiological effects. Serial plasma concentrations of THC and CBD were measured. THC (0:1) was associated with impaired delayed verbal recall (t(45) = 3.399, *d* = 0.50, *p* = 0.001) and induced positive psychotic symptoms on the PANSS (t(45) = −4.709, *d* = 0.69, *p* = 2.41 × 10^–5^). These effects were not significantly modulated by any dose of CBD. Furthermore, there was no evidence of CBD modulating the effects of THC on other cognitive, psychotic, subjective, pleasurable, and physiological measures. There was a dose-response relationship between CBD dose and plasma CBD concentration, with no effect on plasma THC concentrations. At CBD:THC ratios most common in medicinal and recreational cannabis products, we found no evidence that CBD protects against the acute adverse effects of cannabis. This should be considered in health policy and safety decisions about medicinal and recreational cannabis.

## Introduction

Several countries and US states have decriminalised or legalised cannabis use, and many permit the use of cannabis preparations for medicinal purposes. Over a similar period, the potency of cannabis, as indexed by its ∆9-tetrahydrocannabinol (THC) content, has been progressively increasing [1]. THC can cause acute impairments in memory and attention and psychotic symptoms among infrequent users [2–4]. In the longer term, using cannabis with a high THC content may increase the risk of developing a psychotic disorder [5] and cannabis use disorder [6].

As well as THC, cannabis also contains cannabidiol (CBD), which has very different effects. CBD does not impair cognitive performance, and has antipsychotic properties [7]. Both frequent and infrequent cannabis users who smoke varieties of cannabis with a high CBD content have a lower risk of cognitive impairments [8] and psychotic symptoms [9]. Some studies in infrequent users have reported that pre-treatment with CBD attenuates acute THC-induced memory impairments and psychotic symptoms [10], but others, in more frequent users, have not [11].

These findings suggest that cannabis with a relatively high CBD:THC ratio may be less likely to have adverse effects than cannabis with a low CBD:THC ratio. The present study sought to investigate this by examining the acute effects of cannabis containing four different CBD:THC ratios (0:1, 1:1, 2:1 and 3:1) on cognitive performance and psychotic symptoms in healthy volunteers. These ratios were selected to reflect the CBD:THC ratios typically found in recreational cannabis, and in medicinal cannabis products [1, 12, 13]. We tested the hypothesis that administration of cannabis with higher CBD:THC ratios would be associated with less memory impairment and fewer psychotic symptoms.

## Methods

The study was approved by the King’s College London Research Ethics Committee (RESCMR-16/17-4163). All participants provided written informed consent and the study was conducted in compliance with the principles of Good Clinical Practice, the Declaration of Helsinki (1996). The study was registered on Open Science Framework (https://osf.io/kt3f7) and clinicaltrials.gov (NCT05170217).

### Design

This randomised, double-blind, four-arm, within-subjects study was conducted at the NIHR Wellcome Trust Clinical Research Facility (CRF) at King’s College Hospital, London, UK (randomisation and masking described in Appendix pp2). Participants attended a baseline session, followed by four experimental visits, with a minimum one-week wash-out period between each experimental visit (average duration between experiments was 24 days).

### Participants

Forty-six healthy volunteers (age 21–50 years), who had used cannabis at least once in the past, but had not used cannabis >1/week over the last 12 months, had never used synthetic cannabinoids, and did not have a substance use disorder were recruited. Additional inclusion/exclusion criteria are listed in Appendix pp2.

### Procedure (Fig. 1)

At baseline, participants were assessed for study eligibility, and practiced the inhalation procedure. At baseline and all experimental visits, urine drug and pregnancy screen as well as alcohol and carbon monoxide breath tests (<10 ppm CO to verify 12 h tobacco abstinence) were completed. Participants were asked to avoid using cannabis and all other illicit drugs during the entire course of the study, including the periods between sessions.

**Fig. 1:**
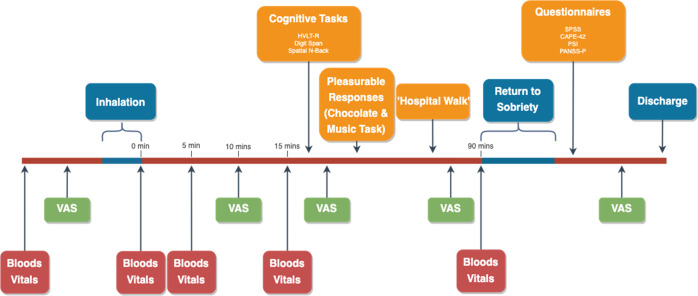
Timeline of baseline and experimental sessions (baseline did not include bloods or return to sobriety).

Prior to each experimental visit participants had their usual breakfast and amount of caffeine – caffeine was not allowed again until completion of cognitive tests. An intravenous cannula was inserted before participants were administered vaporised cannabis (detailed below). Fifteen minutes after the completion of cannabis inhalation, participants completed a battery of cognitive tasks (30–35 min). This was followed by assessments of pleasurable responses to cannabis as well as a ‘hospital walk’ (15 min), a task previously been found to increase paranoia following THC [14]. In this task, participants were given £2 to purchase an item of their choice from a till operator in the hospital shop and to ask for a receipt before returning to the CRF. The research team observed from a distance for safety purposes. Participants were then given lunch and enough of a break to allow any intoxicating effects to wear off. When participants felt that at least 90% of the drug effect had subsided they completed the psychological questionnaires (CAPE, PSI and SSPS, detailed below) and a semi-structured clinical interview (PANSS-P, detailed below). This approach allows the scales to capture all the symptoms which have occurred throughout the experiment, as opposed to those that are evident at a particular time point. We have previously found that assessing participants after the maximal phase of acute intoxication has subsided increases the likelihood of them disclosing delusional thoughts or suspiciousness [10, 15]. Participants were discharged after a field sobriety test, having been informed of safety protocols, and provided with a 24 h emergency number.

### Study drug and administration

The study drug was provided in the form of granulated cannabis inflorescence by Bedrocan BV (Netherlands) produced in accordance with Good Manufacturing Practice and confirms to the European Medicines Agency’s contaminant levels for products used in the respiratory tract. Each cannabis dose consisted of 10 mg of THC (two standard THC units [16]) and either 0 mg, 10 mg, 20 mg, or 30 mg of CBD. Participants were given preparations with CBD:THC ratios of 0:1, 1:1, 2:1, and 3:1, in a random order across visits. *Bedrocan* (22.6% THC, 0.1% CBD), *Bedrolite* (7.5% CBD, 0.3% THC) and Bedrocan placebo (<0.01% THC) were used to provide cannabis containing THC, CBD and placebo, respectively. The placebo cannabis was added to ensure that all preparations had the same weight (see Appendix pp4).

Cannabis preparations were administered using a Volcano® Medic Vaporiser (Storz-Bickel GmbH, Tüttlingen, Germany). Each preparation was vaporised at 210 °C into a transparent polythene bag. This temperature has been found to maximise cannabinoid delivery [17]. Once filled, the transparent bag was encased with an opaque bag to ensure blinding (a higher CBD:THC ratio produces a denser vapour). Inhalation was standardised by asking participants to hold their breath for 8 s before exhaling, with an 8 s break between inhalations (as described in [18]). Participants were asked to inhale a comfortable amount of vapour on each inhalation to minimise the risk of loss of study drug through coughing. The procedure continued until the contents of two bags had been emptied – all participants successfully inhaled the entire contents of both bags on all visits. The inhalation duration of each visit was recorded, and the severity of participant coughing was rated by the researchers using a visual analogue scale. A cup of warm lemon and honey water was provided to help with the abrasiveness of cannabis inhalation.

### Blood collection and analysis

Venous blood samples were taken before drug administration, and at 0, 5, 15, and 90 min following the final exhalation, alongside blood pressure, heart rate and temperature. The concentration of Δ9-THC, 11-OH-Δ9-THC (OH-THC), 11-COOH-Δ9-THC (COOH-THC), CBD and 7-OH-CBD were determined using high performance LC/MS at the Mass Spectrometry Facility, KCL [19].

### Cognitive tasks

#### Hopkins verbal learning task—Revised (HVLT-R) [20]

A researcher read out a list of 12 words to the participant, who then repeated the list back. This was repeated over three trials, with the total number of words recalled indexing immediate recall. 20–25 min later participants were asked to recall the words again, indexing delayed recall. The percentage of correctly recalled words indexed retention. Recalled words that were related to the words in the original list, but not part of it, were defined as intrusions. Repetitions referred to the number of times a correctly recalled word was repeated. A different word list was used on each study visit and the order was randomised.

#### Forward and reverse digit span

Digit span is a measure of verbal working memory and attention, involving the recall of sequences of numbers with increasing length (WAIS-III). Beginning with three digits on forward and two digits on reverse, the task ceased when the participant failed two consecutive attempts at a number sequence.

#### Spatial N-back [21]

Participants responded to a visual stimulus appearing in one of eight locations, with task demand varied across 0-back, 1-back, and 2-back conditions. Participants were required to indicate (by pressing a Yes or No button) whether the stimulus appeared at the 12 o’clock position (0-back), the same position as the previous visual stimulus (1-back), or the same position as the visual stimulus two previous (2-back).

### Psychological measures

#### Positive and negative syndrome scale—positive subscale (PANSS-P) [22]

The PANSS-P is an investigator-rated semi-structured interview, which assesses positive psychotic symptoms (delusions, conceptual disorganisation, hallucinations, hyperactivity, grandiosity, suspiciousness, and hostility). Information from this assessment was supplemented by the researcher’s observations of, and interactions with the participant, while they were intoxicated.

#### State social paranoia scale (SSPS) [23]

The SSPS was used to assess persecutory thoughts.

#### Community assessment of Psychic Experiences—state (CAPE-state) [24]

The CAPE-state is a self-rated scale and was used to assess psychotic-like experiences.

#### Psychotomimetic states inventory (PSI) [25]

The PSI questionnaire was used to assess psychotic-like experiences following the use of cannabis use.

#### Visual analogue scales (VAS)

VAS were used to measure subjective effects along a continuum. Participants marked on a 100 mm horizontal line to indicate the level of a given feeling at that moment (0 mm ‘Not at all’ to 100 mm ‘Extremely’). The feeling states included: *‘feel drug effect’, ‘like drug effect’, ‘want more drug’, ‘mentally impaired’, ‘dry mouth’, ‘enhanced sound perception’, ‘enhanced colour perception’, ‘want food’, ‘want alcohol’, ‘high’, ‘calm and relaxed’, ‘tired’, ‘anxious’, ‘paranoid’, ‘stoned’, and ‘pleasure’*. VAS were administered 5 times over the course of the experimental session: pre-drug, 10 min post-drug, after cognitive assessment, after the hospital walk, and finally before discharge. In order to explore drug effects over time, area under the curve (AUC) analyses we included as well as peak effects.

#### Pleasurable responses

Pleasurable effects of cannabis were assessed by the participant rating their enjoyment of a piece of either milk (Marabou) or dark (Lindt 70%) chocolate, and a self-selected piece of music, on a visual analogue scale (VAS), ranging from −5 to +5 on a 100 mm line. The centre of the line (indicated by 0) indicates that the chocolate and music is enjoyed as much as it was at baseline. A negative score indicates that they were enjoyed less compared to baseline, while a positive score indicates that they were more enjoyable.

### Statistical analysis

According to our power calculation, at 80% power and Bonferroni adjusted alpha <0.008, a sample size of *n* = 45 will give a target ES of *d* = 0.5 as a minimum difference of interest for any of the 6 comparisons. The full power calculation for the study is presented in Appendix pp3.

The effect of THC was determined by comparing outcome scores from the baseline visit with those following administration with THC alone (0:1) using paired t-tests. For the primary analysis, we used linear mixed models to assess the effect of varying the CBD:THC ratio on delayed recall on the HVLT-R. The four CBD:THC ratios (0:1, 1:1, 2:1, 3:1) were included as a fixed effect, with participant as a random effect to account for the dependency between repeated measures. All 6 contrasts were of interest (0:1 vs 1:1, 0:1 vs 2:1, 0:1 vs 3:1, 1:1 vs 2:1, 1:1 vs 3:1, 2:1 vs 3:1) and alpha was set according to the results of our power calculation at *p* < 0.008 with the expectation that modulatory effects of CBD could emerge in any one of these comparisons. The same analysis was used for secondary pharmacokinetic, cognitive, psychological, pleasurable, and physiological outcomes. To account for any potential order effects, sensitivity analyses were conducted adding visit into the model as a fixed effect.

For pharmacokinetics, VAS scores and physiological outcomes, both peak effects (0 min for pharmacokinetic and physiological outcomes) and area under the curve (AUC) were investigated. For the AUC analyses, values were baseline corrected before using the spline method using the bayestestR package (version 0.7.5.1) [26]. Potential differences in VAS scores for ‘feel paranoid’ between the ‘post-cognition’ and ‘post-walk’ timepoints were assessed using paired t-tests to assess the effect of the walk on paranoia.

The relationships between both inhalation time and coughing with peak plasma THC and CBD, in addition with their respective AUCs, were assessed using Pearson’s correlation coefficients.

We additionally categorised clinically significant psychotic-like reactions as increases in PANSS scores from baseline of ≥3 points, as in previous studies due to floor effects [27, 28]. Similarly, we categorised any increase in SSPS score from baseline. The difference in the frequency of these reactions across CBD:THC ratios was analysed using Pearson’s Chi-square test.

Multiple imputation chain equations (MICE) were used to impute missing values in pharmacokinetic, cognitive, pleasurable, and physiological outcomes using the mice package (version 3.13.0) [29], following no detection of deviation from missing completely at random (MCAR) based on Little’s MCAR test.

All analyses were conducted using R version 3.5.3. lme4 (version 1.1-26) [30] was used to fit the linear mixed effects models and estimated marginal mean (EMM) contrasts were calculated using the emmeans package (version 1.5.2-1) [31].

## Results

### Participants and demographics

**Fig. 2:**
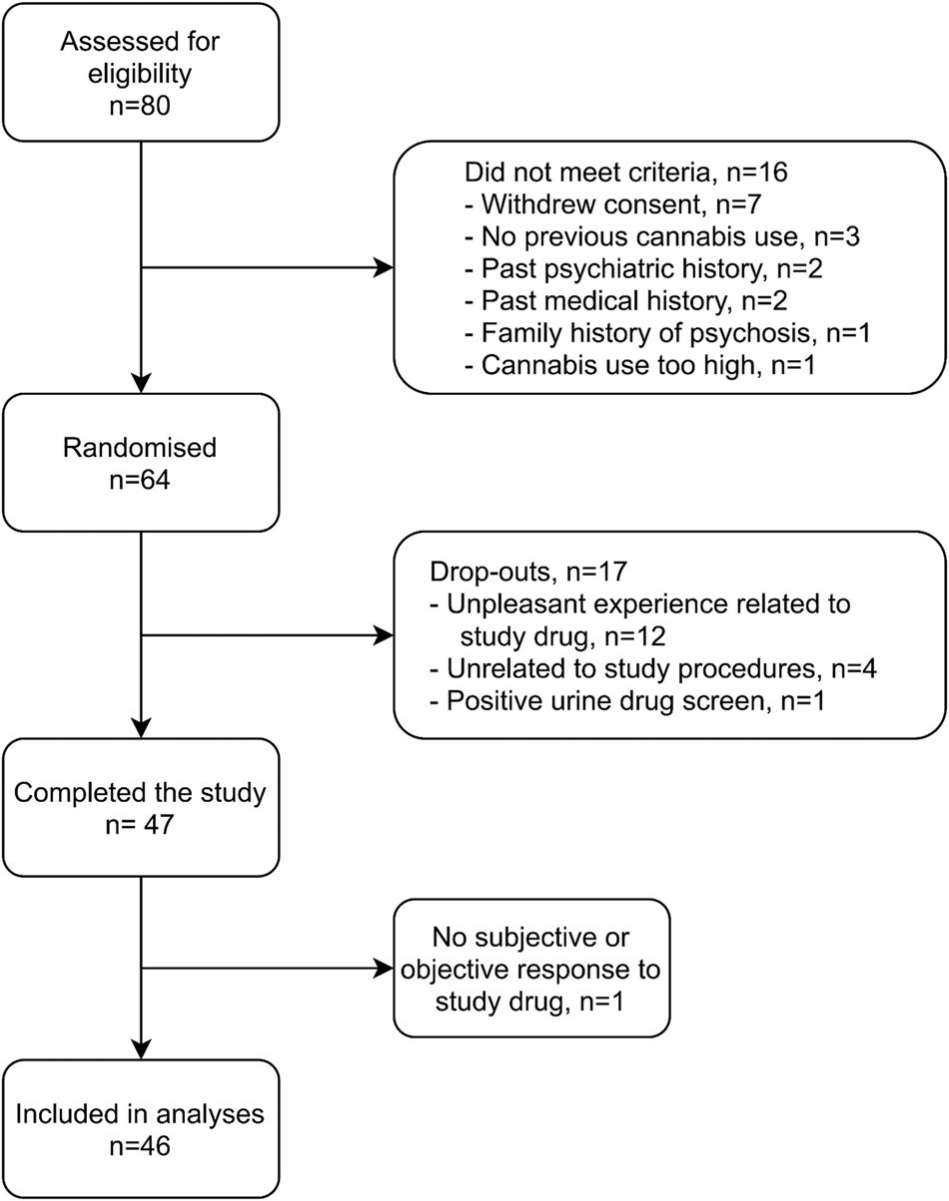
Study flow diagram.

80 potential participants were screened from which 64 were randomised and 46 completed the study (Fig. 2) between November 2017 and June 2019. Of the 18 randomised participants who were later excluded (one excluded at completion, two following the second visit, and the remaining did not complete their first visit), 12 dropped out due to unpleasant drug effects, one due to a positive drug screen, one due to an absence of subjective and objective THC effects, and four for reasons unrelated to study procedures. Of the participants who dropped out on their first visit significantly more dropped out after receiving 3:1, although there was no statistical difference between number of sessions stratified by visit and CBD:THC ratio (Appendix pp5). Demographics for participants who completed the study compared with those who dropped out are presented in Table 1. All analyses were restricted to data from subjects (*n* = 46) with complete datasets.

**Table 1:** Demographics and cannabis use at baseline.

	Completers (*n* = 46)	Drop-outs (*n* = 17)
Sex; *N* (%)		
Male	25 (54.3)	6 (35.3)
Female	21 (45.7)	11 (64.7)
Age; Mean (SD)	26.62 (4.94)	25.88 (4.41)
Ethnicity; *N* (%)		
White	31 (67.4)	12 (70.6)
Asian	11 (23.9)	1 (5.9)
Mixed	3 (6.5)	4 (23.5)
Black	1 (2.2)	0 (0)
Education; *N* (%)		
A Levels	9 (19.6)	2 (11.8)
Vocational	0 (0)	1 (5.9)
University/Professional qualification (degree+)	18 (39.1)	11 (64.7)
Postgraduate degree	19 (41.3)	3 (17.6)
Weight (kg); Mean (SD)	70.68 (11.3)	66.14 (1.97)
BMI (kg/m^2^); Mean (SD)	23.72 (2.57)	22.62 (1.97)
Body Fat (%)- Male; Mean (SD)	15.56 (5.50)	11.76 (3.67)
Body Fat (%)- Female; Mean (SD)	25.50 (6.33)	24.47 (3.27)
Age of first cannabis use; Mean (SD)	17.67 (2.46)	16.71 (2.02)
Years of cannabis use; Median (IQR)	5.50 (6.5)	5.00 (3.00)
Cannabis use occasions in last year; Median (IQR)	5.00 (6.00)	3.00 (7.00)

### Pharmacokinetics

**Fig. 3:**
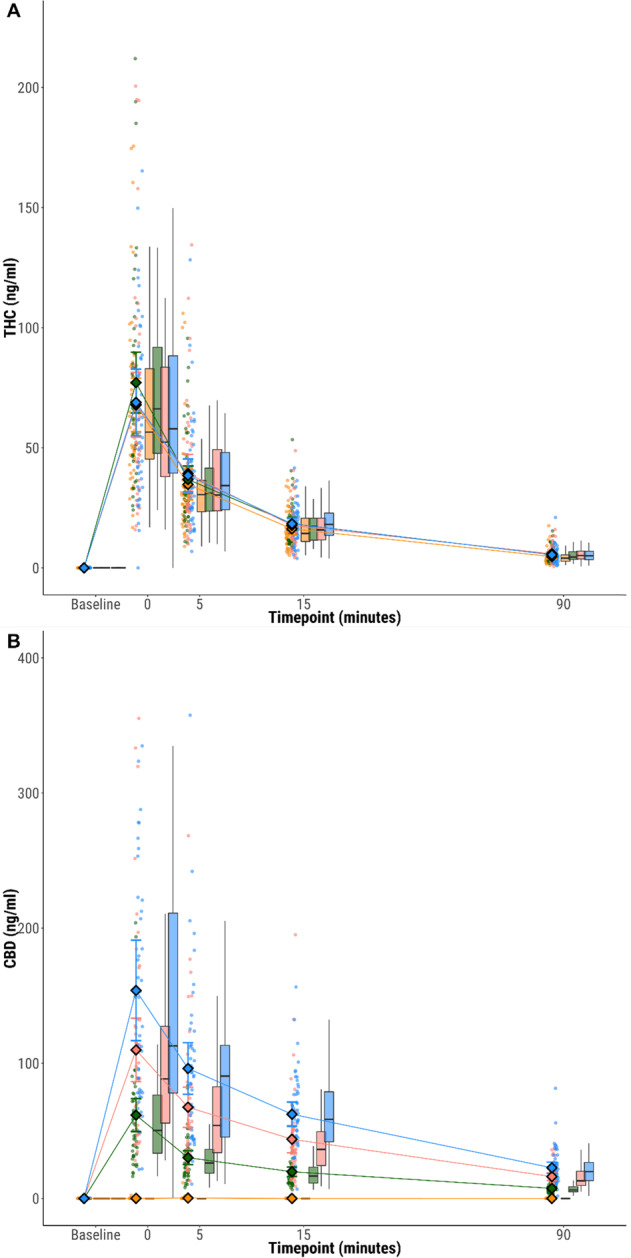
Blood plasma THC and CBD concentrations over time and across CBD:THC ratio. Plasma concentrations of **A** THC, **B** CBD at each time point, stratified by CBD:THC ratio. Circles show individual data points, diamonds show mean values and boxplots show median and interquartile range. CBD:THC ratios 0:1 (orange) 1:1 (green); 2:1 (pink); 3:1 (blue).

There were no significant differences in either peak plasma THC, OH-THC or COOH-THC, or their respective AUCs between the CBD:THC ratios (*p* > 0.008, Fig. 3A, Appendix pp6–12). In contrast, there was a significant, dose-dependent increase in peak plasma CBD, and in plasma CBD AUC, as CBD:THC ratio increased (*p* < 0.001, Fig. 3B, Appendix pp6–12). Peak plasma 7-OH-CBD was higher for the 3:1 ratio compared to 0:1 (EMM difference = 2.686, 95%CI: 1.888, 3.483, *p* = 1.25 × 10^−9^) and 1:1 (EMM difference = 2.206, 95% CI: 1.551, 2.861, *p* = 0.002), with AUC higher for 2:1 compared to 0:1 (EMM difference = 4.676, 95% CI: 3.287, 6.064, *p* = 0.003) and for 3:1 compared to 0:1 EMM difference = 8.898, 95%CI: 6.256, 11.540, *p* = 1.71 × 10^−9^) and 1:1 (EMM difference = 6.843, 95% CI: 4.811, 8.875, *p* = 3.57 × 10^−6^). Logarithmic concentrations of THC and CBD over time, with intercept and slope across ratios are presented in Appendix pp13–14.

### Cognitive effects

#### Hopkins verbal learning task

**Fig. 4:**
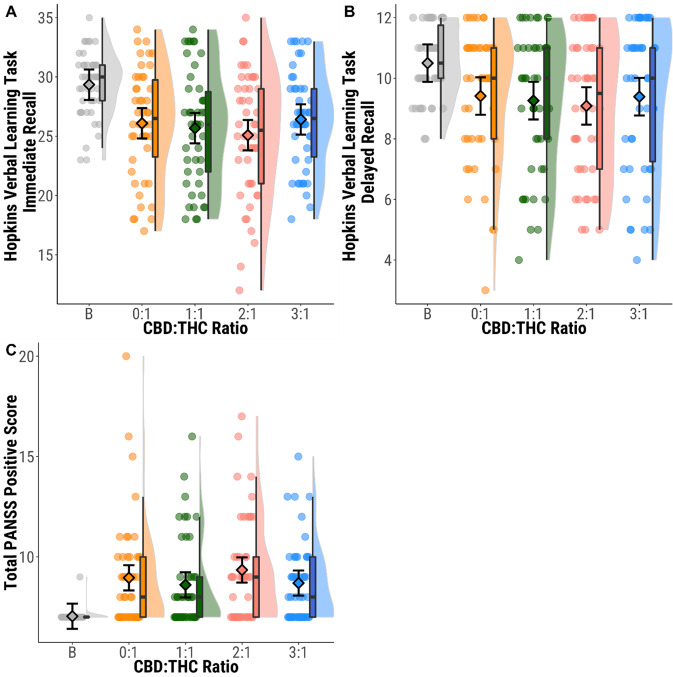
Immediate and delayed verbal recall, and psychotic symptoms across CBD:THC ratios compared to baseline. **A** HVLT-R Immediate recall (number of words recalled across three encoding trials) **B** HVLT-R Delayed recall (number of words recalled from encoding phase). **C** PANSS positive subscale symptom score. Circles show individual data points, diamonds show mean values, boxplots show median and interquartile range, and half violin plots show distribution of participant scores. Baseline (B; grey), CBD:THC ratios 0:1 (orange) 1:1 (green); 2:1 (pink); 3:1 (blue).

When the 0:1 condition (THC only) was compared to baseline, there were impairments in both immediate (t(45) = 5.580, *d* = 0.82, *p* = 1.31 × 10^−6^) and delayed recall (t(45) = 3.399, *d* = 0.50, *p* = 0.001), and higher rates of intrusion in both conditions (t(45) = −3.824, *d* = 0.56, *p* = 4.02 × 10^−4^; t(45) = −3.322, *d* = 0.49, *p* = 0.002). However, there were no significant differences on any measure of performance between the different CBD:THC ratios (*p* > 0.008, Fig. 4A, B, Appendix pp15–23).

#### Digit span

There was significant impairment in the 0:1 condition compared to baseline in forward digit span (t(45) = 3.309, *d* = 0.49, *p* = 0.002), but not for reverse digit span (t(45) = 2.361, *d* = 0.35, *p* = 0.023). There were no significant differences in either forward or reverse digit span between the CBD:THC ratios (*p* > 0.008, Appendix pp16–20).

#### Spatial N-Back

There were no significant differences between baseline and 0:1, or between CBD:THC ratios (*p* > 0.008, Appendix pp16–21).

### Psychological effects

#### PANSS positive subscale

There was a significant increase in PANSS positive score between baseline and 0:1 (t(45) = −4.709, *d* = 0.69, *p* = 2.41 × 10^−5^). 24 participants (52.2%) had an increase of 3 points on the PANSS on at least one visit across 47 visits (25.5%) with a PANSS response (*n* = 12 (26.1%) in the 0:1 condition, *n* = 10 (21.7%) in the 1:1 condition, *n* = 15 (32.6%) in the 2:1 condition, *n* = 10 (21.7%) in 3:1 condition). There were no significant differences in PANSS positive scores (*p* > 0.008, Fig. 4C, Appendix pp25–26) or PANSS response (*X*^2^(3, *n* = 46) = 2.202, *d* = 0.44, *p* = 0.532) between CBD:THC ratios.

#### SSPS

There were no significant differences in SSPS scores between baseline and 0:1, between CBD:THC ratios (t(45) = −1.096, *d* = 0.16, *p* = 0.279, Appendix pp24–27), or SSPS response between CBD:THC ratios (*X*^2^(3, *n* = 46) = 5.4474, *d* = 0.73, *p* = 0.142).

#### CAPE

There was a significant increase in total CAPE score between baseline and 0:1 (t(45) = −4.088, *d* = 0.60, *p* = 0.0002) but not between CBD:THC ratios (*p* > 0.008, Appendix pp24–26).

#### PSI

There was a significant increase in total PSI score between baseline and 0:1 (t(39) = −7.461, *d* = 1.18, *p* = 5.025 × 10^−9^) but not between CBD:THC ratios (*p* > 0.008, Appendix pp24–27).

#### VAS

There were no significant differences in subjective effects between CBD:THC ratios in terms of either VAS AUC or peak VAS ratings (*p* > 0.008, Appendix pp28–42). There were no significant correlations between VAS measures of feeling high (either peak or AUC) with plasma THC or CBD (either peak or AUC) (p > 0.008, Appendix pp51–52).

#### Pleasurable responses

All CBD:THC ratios increased scores for both chocolate and music compared to baseline, but there were no significant differences between the CBD:THC ratios (*p* > 0.008, Appendix pp43–45).

### Physiological effects

#### Blood pressure

There were no significant differences in systolic (t(44) = −1.19, *d* = 0.18, *p* = 0.240) or diastolic blood pressure between baseline and the 0:1 condition (t(44) = 0.312, *d* = 0.05, *p* = 0.756). There were no significant differences between CBD:THC ratios in peak systolic, diastolic blood pressure or AUC (*p* > 0.008, Appendix pp46-49).

#### Heart rate

There was a significant increase in heart rate in the 0:1 condition compared to baseline (t(44) = −9.35, *d* = 1.39, *p* = 5.06 × 10^−12^).There were no significant differences between CBD:THC ratios in peak heart rate or AUC (*p* > 0.008, Appendix pp46–49).

#### Body temperature

There were no significant differences in body temperature between any of the conditions (Appendix pp46–50).

#### Inhalation and coughing

There was evidence of greater CBD:THC ratios increasing inhalation time and coughing in a dose responsive manner (Appendix pp46–50). Greater inhalation time was correlated with lower peak and AUC concentrations of cannabinoids at higher CBD:THC ratios (Appendix pp51–52).

#### Order and sex effects

Adding order to the models did not have any impact on the significance or direction of pharmacokinetic, cognitive, psychological, subjective, pleasurable, or physiological effects. Restricting the analysis of the primary outcome to visit 1 found no differences across conditions, suggesting no evidence for significant practice or fatigue effects (Appendix pp53). There were no additional significant differences when analysis was stratified by sex on any measure (Appendix pp54–90).

## Discussion

Our main finding is that the co-administration of CBD with THC had no effect on the induction of either cognitive impairments or psychotic symptoms following cannabis use. Similarly, CBD did not influence the subjective (as measured by VAS) or the pleasurable effects (music and chocolate) of THC. This was true across the range of CBD:THC dose ratios that are typically present in both recreational and medicinal cannabis [13]. Because we detected robust effects of THC on cognitive performance and psychotic symptoms and studied a relatively large number of subjects (given the within-subject design), it is unlikely that the absence of a modulatory effect of CBD was due to a lack of statistical power. Furthermore, THC failed to show a significant effect on reverse digit span, spatial N-back, SSPS, blood pressure and body temperature.

Using a within-subjects design minimised the potentially confounding effects of inter-individual differences in responses to THC and CBD [32], while confounding effects of previous cannabis use and of placebo responses were reduced by ensuring that the participants were infrequent users and were blind to the content of the preparations. Some participants dropped out of the study because they could not tolerate the symptomatic effects of THC, raising the possibility that those who completed it may have been less sensitive to these effects. However, among those who completed the study, THC induced significant changes on three independent psychopathological instruments, as well as significant impairments in memory and attention. Furthermore, there was a significantly greater number of participants who dropped out on their first visit when administered the 3:1 ratio. However, as we did not observe a dose response, we find it unlikely that there was a specific CBD effect on drop-outs. Previous studies have found that females experience similar subjective effects of cannabis to males at lower doses of THC, suggesting a greater sensitivity towards cannabinoids [33]. However, in the present study, we found no additional differences when stratifying analyses by sex on any measure, across all CBD:THC ratios—suggesting that females and males respond the same to cannabis when administered the same dose. However, our study was not powered to explore sex differences and further studies with larger male and female subgroups may be needed to clarify if such differences exist.

Including an additional placebo arm might have made it easier to establish the effects of THC. However, the focus of the study was to compare cannabis with different CBD:THC ratios, rather than to examine the effects of THC alone. The latter have been described in previous studies, and the cognitive and psychological effects of THC that we observed relative to baseline were in line with those reported relative to placebo in infrequent users [2, 3]. Serial measurements of the plasma concentrations of CBD, THC and their metabolites indicated that the findings were not attributable to pharmacokinetic effects. However, longer inhalation time was associated with decreased peak and AUC plasma CBD and THC, although only within higher CBD:THC ratios.

Our findings are consistent with previous reports that co-administration of CBD with THC did not alter the effects of THC on memory, psychotic symptoms [11], performance on a reward task (in frequent users) [18], or driving (in infrequent users) [34]. Studies that examined the impact of pre-treatment with CBD on the effects of THC have had more mixed results. Pre-treatment with oral CBD did not alter the effect of THC on attention and processing speed in infrequent users [35], and did not change the subjective effects of inhaled THC in frequent cannabis users [36]. In contrast, two studies in infrequent users reported that administration of CBD prior to intravenous THC attenuated the induction of psychotic symptoms and memory impairments [27, 37]. The latter studies used relatively large doses of CBD (5 mg i.v. and 600 mg orally, respectively), raising the possibility that we might have seen similar effects if we had used cannabis with higher CBD:THC ratios than those usually present in recreational and medicinal cannabis. However, higher CBD:THC ratios may be impractical when inhaled as a previous study found participants were only able to inhale 62.5% of the high-CBD condition (50:1 CBD:THC ratio; 400 mg CBD, 8 mg THC) [38].

There are other mechanisms by which cannabis with higher CBD:THC ratios may be less harmful to users. The cannabis plant produces both THC and CBD (in their acid forms) from a precursor named cannabigerolic acid [39], which implies that a plant with a higher CBD:THC ratio will produce less THC than a THC-dominant one. The purported reduced risk from using high CBD varieties (cognitive impairment and psychosis) may thus not be an effect of the high CBD content, but due to the relatively low THC content. This issue could be addressed in studies with a similar design to the present one, but with experimental manipulation of the dose of THC, rather than of CBD. Lastly, the present study found that CBD did not acutely protect against the effects of THC - future studies should explore if the presence of CBD in cannabis may protect against the long-term harms of cannabis use.

## Conclusions

At the doses typically present in recreational and medicinal cannabis, we found no evidence of CBD reducing the acute adverse effects of THC on cognition and mental health. Similarly, there was no evidence that it altered the subjective or pleasurable effects of THC. These results suggest that the CBD content in cannabis may not be a critical consideration in decisions about its regulation or the definition of a standard THC unit [16, 40]. The data are also relevant to the safety of licensed medicines that contain THC and CBD, as they suggest that the presence of CBD may not reduce the risk of adverse effects from the THC they contain. Cannabis users may reduce harms when using a higher CBD:THC ratio, due to the reduced THC exposure rather than the presence of CBD. Further studies are needed to determine if cannabis with even higher ratios of CBD:THC may protect against its adverse effects.

## Supplementary information

eCBD Appendix3
