# Supplementary material for: Does cannabidiol make cannabis safer? A randomised, double-blind, cross-over trial of cannabis with four different CBD:THC ratios
Source: Neuropsychopharmacology. 2022 Nov 16;48(6):869–76. doi: 10.1038/s41386-022-01478-z (PMC10156730; doi:10.1038/s41386-022-01478-z)
Supplement: Supplementary file 1 — eCBD Appendix3 [file 41386_2022_1478_MOESM1_ESM.docx]

# **Appendix**

Englund A, Oliver D, Chesney E, Chester L, Wilson J, Sovi S, De Micheli A, Hodsoll J, Fusar-Poli P, Strang J, Murray R, Freeman T, McGuire P. Does cannabidiol make cannabis safer? A randomised, double-blind, cross-over trial of four CBD:THC ratios in healthy volunteers

**eMethods 1** Full inclusion and exclusion criteria

**eMethods 2** Randomisation and masking

**eMethods 3** Power calculations

**eTable 1** Weight of Bedrocan, Bedrolite and placebo cannabis in each CBD:THC ratio

**eTable 2** Dropouts by study visit and reasons for drop out

**eFigure 1** Plasma cannabinoid levels stratified by CBD:THC ratio

**eFigure 2** Plasma cannabinoid area under the curve stratified by CBD:THC ratio

**eTable 3** Pharmacokinetics stratified by CBD:THC ratio

**eTable 4** Results of linear mixed models for pharmacokinetic outcomes

**eTable 5** Logarithmic concentrations of THC and CBD over time, with intercept and slope across ratios

**eFigure 3** Logarithmic concentrations of THC and CBD over time

**eFigure 4** Hopkins Verbal Learning Task – Revised performance stratified by CBD:THC ratio

**eFigure 5** Digit span and Spatial N-back performance stratified by CBD:THC ratio

**eTable 6** Cognitive performance stratified by CBD:THC ratio

**eTable 7** Results of linear mixed models for cognitive outcomes

**eFigure 6** Box and whisker plots for individual slopes and distribution of intercepts for delayed recall on the HVLT-R

**eTable 8** Effect sizes across ratio contrasts (Cohen’s d and 95% CI) for delayed recall on the HVLT-R

**eFigure 7** Psychological effects stratified by CBD:THC ratio

**eTable 9** Psychological effects stratified by CBD:THC ratio

**eTable 10** Results of linear mixed models for psychological outcomes

**eFigure 8** Subjective drug effects stratified by CBD:THC ratio

**eFigure 9** Subjective drug effects area under the curve stratified by CBD:THC ratios

**eTable 11** Subjective drug effects measured by visual analogue scales (VAS) stratified by CBD:THC ratio

**eTable 12** Results of linear mixed models for subjective drug effects

**eFigure 10** Pleasurable response ratings for chocolate and music stratified by CBD:THC ratio

**eTable 13** Pleasurable response ratings for chocolate and music stratified by CBD:THC ratio

**eTable 14** Results of linear mixed models for chocolate and music

**eFigure 11** Physiological outcomes stratified by CBD:THC ratio

**eTable 15** Physiology stratified by CBD:THC ratio

**eTable 16** Results of linear mixed models for physiological outcomes

**eFigure 12** Inhalation time correlation matrices

**eFigure 13** Coughing correlation matrices

**eTable 17** Delayed recall performance on the HVLT-R for Visit 1 across ratios compared with all visits combined

**eTable 18** Gender differences across ratios on all outcomes

**eMethods 1** Full inclusion and exclusion criteria

For inclusion into the study each participant needs to fulfil ALL of the following criteria:

- Be male or female aged between 21-50 years old

- Have used cannabis at least once in the past

- Willing and able to provide written informed consent

- Willing to provide blood samples

- Be a fluent English speaker

The participant will be excluded if they fulfil ONE OR MORE of the following criteria:

- Having a past or present major mental illness

- Having a past or present major physical illness (e.g. respiratory or heart condition)

- Having a past or present substance use disorder

- Having ever been treated with an anti-psychotic or anti-depressant medication

- Having a first degree relative (parent or sibling) with psychotic illness

- Currently taking psychotropic medication

- Having a positive urine drug screen (any drug) at any of the study visits (baseline and experimental sessions)

- Having used alcohol within 24h an experimental visit or tobacco on the same day (measured by alcohol breath test and CO breath test)

- Being pregnant or planning pregnancy or lactating in women

- Discovery of significant abnormality during physical examination at screening visit

- Is using cannabis (defined as days in which cannabis is used recreationally) more often than once per week on average over the last 12 months.

- Any past use of synthetic cannabinoids

- Having a score of 5 or more on the Fagerstrom Nicotine Dependence Questionnaire

- Having a BMI classified as obese or underweight (below 18 or over 30)

- Having participated in a drug study within the last 30 days or taking part in another research study over the course of the study

- Having a known sensitivity or allergy towards cannabis or Lorazepam

**eMethods 2** Randomisation and masking

The study design was a 4 phase cross-over with each phase corresponding to one of the 4 CBD:THC dosing ratios: 0:1, 1:1, 2:1, 3:1. Each participant was required to complete all phases corresponding to all ratios of cannabis preparation.. For 4 conditions there are 24 possible permutations of sequential order, e.g. Visit 1, 2:1 – Visit 2, 0:1 – Visit 3, 1:1 – Visit 4, 3:1*.* Randomised sequences were generated in blocks with the first 24 participants allocated each of the 24 possible order of ratios, as were the next 24 and so on. Where there were fewer participants than possible order sequences, each participant received a random selection from the 24, sampled without replacement. The randomisation list was generated by a statistician not involved with the study, using a customised randomisation script generated in R software v 3.1 (available on request).

The randomisation was double blinded to both researchers and participants. The randomisation list was passed from the independent statistician to the Maudsley Pharmacy who prepared the cannabis preparations. The pharmacy dispensed the study drug to a blinded researcher. The cannabis preparation was then loaded into the filling chamber and vaporised by a research nurse at the Clinical Research Facility (CRF) who was not involved with any other study procedures. Upon completion of data collection and entry the randomisation schedule was revealed to the research team prior to data analysis.

**eMethods 3** Power calculations

The study is powered based on our primary outcome measure (change in delayed verbal recall on the HVLT-R), specifically the change in our primary outcome between the smallest ratio-contrast (0:1 versus 1:1, CBD:THC). We have estimated the likely effect size of d=1 based on our previous study, a between-subject study with oral CBD or placebo pre-treatment prior to intravenous THC administration (Englund et al., 2013). The assumption here is that the CBD group from this study (compared to placebo) represents the greatest CBD:THC ratio-contrast (0:1 versus 3:1, CBD:THC) proposed in our study.

However, as the aim of this study is to explore the dose-response relationship between the four CBD:THC ratios – we are also interested in the difference between the smallest ratio-contrasts (0:1 versus 1:1, CBD:THC). Unfortunately, there are no studies to date which have explored this before and we have therefore decided to approximate the effect size to half that of the larges ratio-contrast, d=0.5. In the absence of other evidence we feel d=0.5 is a fair estimation of the likely effect size between 0:1 and 1:1 CBD:THC ratio, which would yield a sample size of n=45. A power calculation indicates that this would provide 80% power to detect differences between the cannabis preparations with largest differences in CBD: THC ratio (0:1 to 3:1) at alpha = 0.008 (paired t-test; adjusted for multiple comparisons). As we expect a drop-out rate of 25% (20-30% from previous studies) we aim to recruit 56 participants.

**eTable 1** Weight of Bedrocan, Bedrolite and placebo cannabis in each CBD:THC ratio

| CBD:THC ratio | 0:1 | 1:1 | 2:1 | 3:1 |
| --- | --- | --- | --- | --- |
| THC dose (mg) | 10 | 10 | 10 | 10 |
| CBD dose (mg) | 0 | 10 | 20 | 30 |
| Bedrocan cannabis (mg) | 44.2 | 42.5 | 40.7 | 38.9 |
| Bedrolite cannabis (mg) | 0.0 | 132.8 | 266.1 | 399.5 |
| Placebo cannabis (mg) | 394.2 | 263.1 | 131.6 | 0.0 |
|  |  |  |  |  |

**eTable 2** Upper table: number of participants receiving study drug at each visit, stratified by CBD:THC ratio. Number of participants who completed each visit are presented in parentheses. Lower table: reasons for drop out stratified by CBD:THC ratio. There were no significant differences in number of sessions completed stratified by visit and CBD:THC ratio (χ(9) = 6.800, p-value = 0.658)

|  |  |  | | |  |  |  |  |
| --- | --- | --- | --- | --- | --- | --- | --- | --- |
|  |  | |  | **CBD:THC Ratio** | | | | |
|  |  | |  | **0:1** | | **1:1** | **2:1** | **3:1** |
|  | **Visit** | | **1** | 14 (12) | | 16 (15) | 16 (14) | 17 (7) |
|  |  |  | **2** | 13 (12) | | 12 (11) | 11 (11) | 12 (12) |
|  |  |  | **3** | 13 (12) | | 10 (10) | 12 (12) | 11 (11) |
|  |  |  | **4** | 10 (10) | | 11 (11) | 9 (9) | 16 (16) |
|  | **Total dropouts** | | | 3 | | 2 | 2 | 10 |
|  | **Reason for drop-out** | | |  | |  |  |  |
|  | **Unpleasant drug experience** | | | 1 | | 0 | 1 | 4 |
|  | **Nausea/vomiting** | | | 1 | | 0 | 0 | 2 |
|  | **Overintoxication** | | | 0 | | 1 | 0 | 2 |
|  | **Unrelated to study procedures** | | | 1 | | 1 | 1 | 2 |

**eFigure 1** Plasma levels of **A** OH-THC, **B** COOH-THC and **C** 7-OH-CBD at each time point, stratified by CBD:THC ratio. Circles show individual data points, diamonds show mean values and boxplots show median and interquartile range.

**
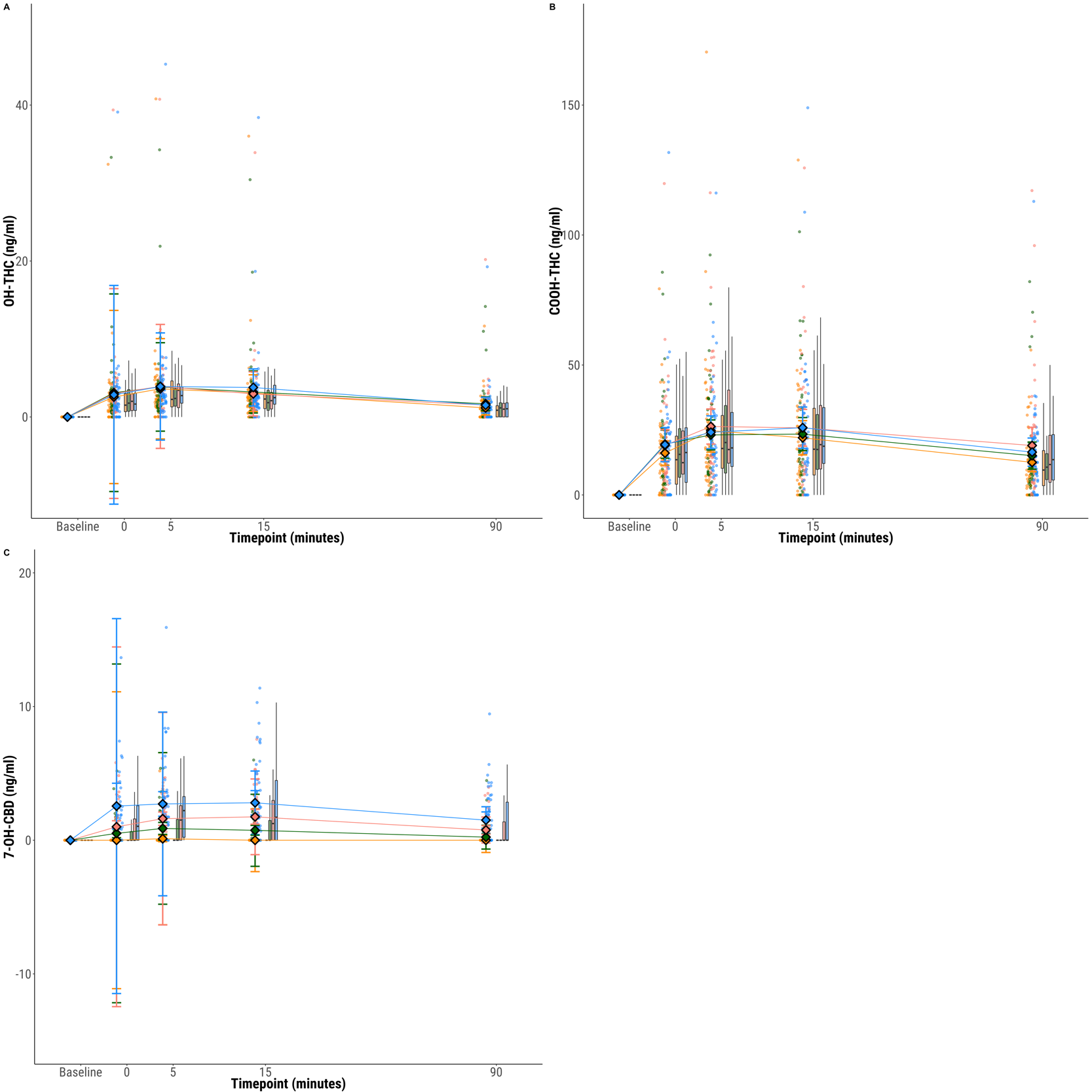
**

0:1

1:1

2:1

3:1


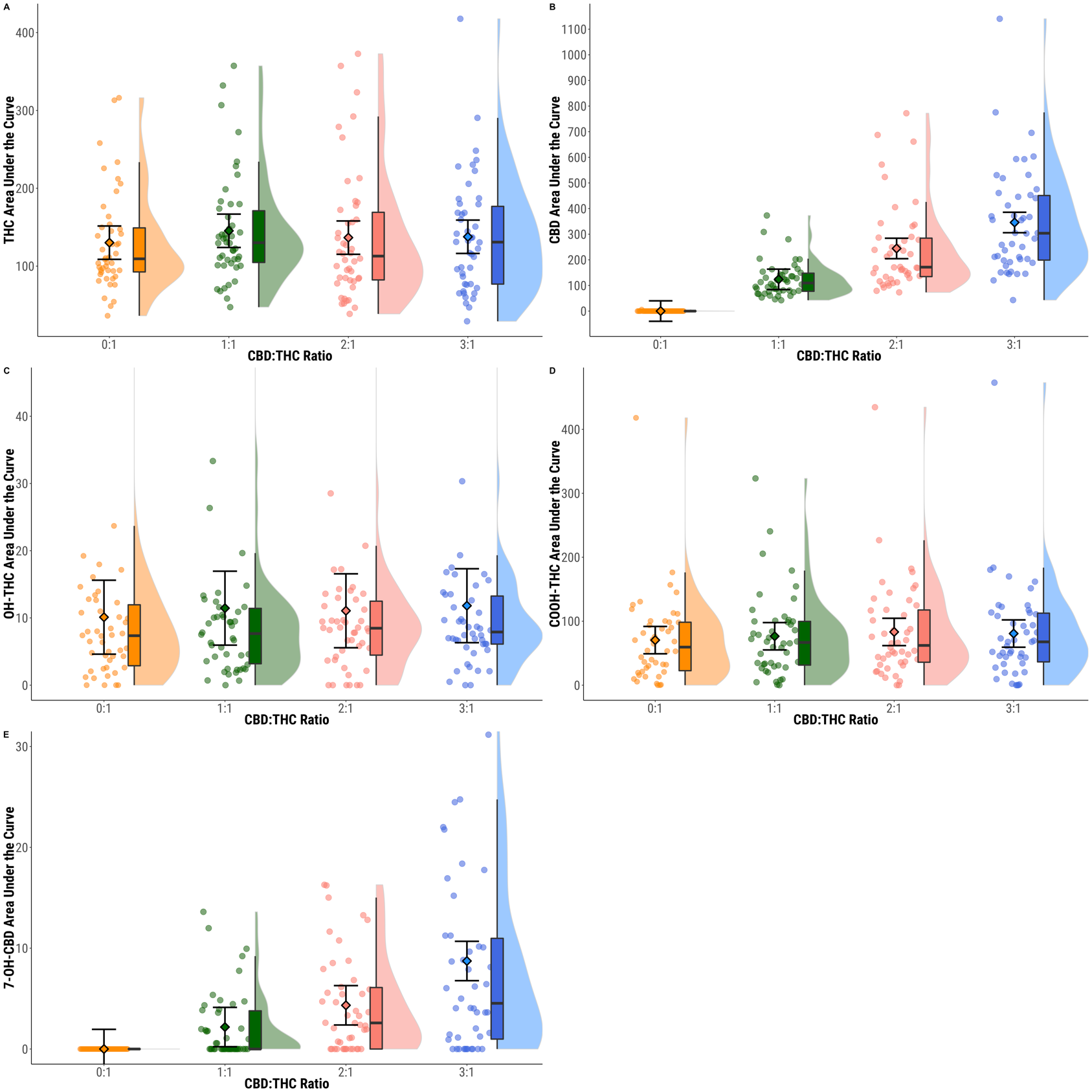


**eFigure 2** Plasma area under the curve of **A** OH-THC, **B** COOH-THC and **C** 7-OH-CBD stratified by CBD:THC ratio. Circles show individual data points, diamonds show mean values, boxplots show median and interquartile range and half violin plots show the distribution of participants.

0:1

1:1

2:1

3:1

| **eTable 3** Pharmacokinetics stratified by CBD:THC ratio. Means (SD) are presented for each time point. C_max_ and t_max_ for each CBD:THC ratio are presented in bold | | | | |
| --- | --- | --- | --- | --- |
|  | **CBD:THC Ratio** | | | |
|  | **0:1** | **1:1** | **2:1** | **3:1** |
| **Outcome** | **THC (ng/ml)** | | | |
| **Baseline** | 0 (0) | 0.01 (0.05) | 0 (0) | 0.01 (0.06) |
| **0 minutes** | **67.85 (37.39)** | **77.11 (42.66)** | **67.93 (45.3)** | **68.31 (47.27)** |
| **5 minutes** | 34.54 (21.64) | 36.8 (19.1) | 39.26 (26.76) | 38.46 (23.14) |
| **15 minutes** | 16.26 (7.91) | 18.37 (9.07) | 17.71 (9.53) | 18.35 (8) |
| **90 minutes** | 4.69 (3.09) | 5.5 (3.01) | 5.85 (3.26) | 5.46 (3.38) |
|  | **CBD (ng/ml)** | | | |
| **Baseline** | 0 (0) | 0 (0) | 0.01 (0.07) | 0 (0) |
| **0 minutes** | **0.05 (0.35)** | **61.59 (40.98)** | **109.83 (79)** | **152.85 (125.86)** |
| **5 minutes** | 0.04 (0.28) | 30.12 (17.19) | 67.43 (50.16) | 96.12 (64.25) |
| **15 minutes** | 0.02 (0.17) | 19.74 (11.82) | 43.76 (33.57) | 62.25 (30.06) |
| **90 minutes** | 0 (0) | 7.43 (4.38) | 16.15 (9.52) | 22.67 (14.06) |
|  | **OH-THC (ng/ml)** |  |  |  |
| **Baseline** | 0 (0) | 0 (0) | 0.01 (0.07) | 0 (0) |
| **0 minutes** | 2.56 (4.90) | 3.11 (5.17) | 3.01 (5.75) | 2.85 (5.73) |
| **5 minutes** | **3.54 (5.98)** | **3.85 (5.82)** | **3.92 (5.99)** | **3.91 (6.53)** |
| **15 minutes** | 3.06 (5.41) | 3.20 (5.17) | 2.97 (5.00) | 3.78 (5.97) |
| **90 minutes** | 1.17 (1.92) | 1.69 (2.77) | 1.52 (3.06) | 1.52 (2.88) |
|  | **COOH-THC (ng/ml)** |  |  |  |
| **Baseline** | 0.01 (0.07) | 0 (0) | 0 (0) | 0 (0) |
| **0 minutes** | 16.10 (15.69) | 19.53 (18.23) | 19.07 (20.28) | 19.35 (22.15) |
| **5 minutes** | **24.62 (27.82)** | 23.09 (19.41) | **26.34 (22.76)** | 24.11 (21.27) |
| **15 minutes** | 22.00 (22.29) | **23.41 (21.41)** | 25.81 (23.89) | **25.88 (26.77)** |
| **90 minutes** | 12.54 (12.29) | 15.12 (17.63) | 19.02 (23.73) | 16.48 (18.25) |
|  | **7-OH-CBD (ng/ml)** |  |  |  |
| **Baseline** | 0 (0) | 0 (0) | 0 (0) | 0 (0) |
| **0 minutes** | 0 (0) | 0.48 (1.07) | 0.95 (1.52) | 2.47 (5.77) |
| **5 minutes** | 0 (0) | **0.77 (1.35)** | **1.66 (1.79)** | 2.68 (3.15) |
| **15 minutes** | **0.05 (0.32)** | 0.75 (1.23) | 1.56 (1.59) | **2.97 (3.10)** |
| **90 minutes** | 0 (0) | 0.21 (0.81) | 0.74 (1.08) | 1.29 (1.94) |

| **eTable 4** Results of linear mixed models of pharmacokinetics for each contrast between CBD:THC ratios. Statistically significant rows are presented in bold | | | | |
| --- | --- | --- | --- | --- |
|  |  | | | |
| **Contrast** | **Estimated marginal mean difference** | **Lower 95% CI** | **Upper 95% CI** | **p-value** |
|  | **THC AUC** |  |  |  |
| 0:1 - 1:1 | -15.336 | -32.679 | 2.008 | 0.287 |
| 0:1 - 2:1 | -6.549 | -23.893 | 10.794 | 0.872 |
| 0:1 - 3:1 | -7.412 | -24.756 | 9.931 | 0.825 |
| 1:1 - 2:1 | 8.786 | -8.557 | 26.130 | 0.738 |
| 1:1 - 3:1 | 7.923 | -9.420 | 25.267 | 0.794 |
| 2:1 - 3:1 | -0.863 | -18.207 | 16.480 | 1.000 |
|  | **THC AUC (visit included in model)** | | | |
| 0:1 - 1:1 | -16.038 | -33.514 | 1.437 | 0.256 |
| 0:1 - 2:1 | -7.112 | -24.571 | 10.347 | 0.845 |
| 0:1 - 3:1 | -6.943 | -24.495 | 10.609 | 0.856 |
| 1:1 - 2:1 | 8.927 | -8.524 | 26.377 | 0.732 |
| 1:1 - 3:1 | 9.095 | -8.499 | 26.689 | 0.726 |
| 2:1 - 3:1 | 0.169 | -17.475 | 17.813 | 1.000 |
|  | **THC Peak** |  |  |  |
| 0:1 - 1:1 | -9.255 | -21.379 | 2.869 | 0.418 |
| 0:1 - 2:1 | -0.085 | -12.209 | 12.039 | 1.000 |
| 0:1 - 3:1 | -1.013 | -13.137 | 11.111 | 0.998 |
| 1:1 - 2:1 | 9.170 | -2.954 | 21.294 | 0.426 |
| 1:1 - 3:1 | 8.242 | -3.882 | 20.366 | 0.521 |
| 2:1 - 3:1 | -0.928 | -13.052 | 11.196 | 0.99 |
|  | **THC Peak (visit included in model)** |  |  |  |
| 0:1 - 1:1 | -9.606 | -21.843 | 2.631 | 0.393 |
| 0:1 - 2:1 | -0.282 | -12.508 | 11.944 | 1.000 |
| 0:1 - 3:1 | -1.158 | -13.449 | 11.132 | 0.998 |
| 1:1 - 2:1 | 9.324 | -2.896 | 21.544 | 0.419 |
| 1:1 - 3:1 | 8.448 | -3.872 | 20.768 | 0.513 |
| 2:1 - 3:1 | -0.876 | -13.232 | 11.479 | 0.999 |
|  | **CBD AUC** |  |  |  |
| **0:1 - 1:1** | **-123.709** | **-168.682** | **-78.736** | **9.088x10^-7^** |
| **0:1 - 2:1** | **-244.322** | **-289.295** | **-199.349** | **1.565x10^-14^** |
| **0:1 - 3:1** | **-345.036** | **-390.009** | **-300.063** | **<2.22x10^-16^** |
| **1:1 - 2:1** | **-120.613** | **-165.586** | **-75.640** | **1.722x10^-6^** |
| **1:1 - 3:1** | **-221.327** | **-266.300** | **-176.354** | **2.975x10^-14^** |
| **2:1 - 3:1** | **-100.714** | **-145.687** | **-55.741** | **8.148x10^-5^** |

|  | **CBD AUC (visit included in model)** |  |  |  |
| --- | --- | --- | --- | --- |
| **0:1 - 1:1** | **-124.426** | **-169.361** | **-79.492** | **7.738x10^-7^** |
| **0:1 - 2:1** | **-245.398** | **-290.354** | **-200.442** | **8.549x10^-15^** |
| **0:1 - 3:1** | **-342.526** | **-387.656** | **-297.397** | **<2.22x10^-16^** |
| **1:1 - 2:1** | **-120.972** | **-165.893** | **-76.050** | **1.570x10^-6^** |
| **1:1 - 3:1** | **-218.100** | **-263.368** | **-172.833** | **2.431x10^-14^** |
| **2:1 - 3:1** | **-97.129** | **-142.478** | **-51.779** | **1.798x10^-4^** |
|  | **CBD Peak** |  |  |  |
| **0:1 - 1:1** | **-61.543** | **-87.712** | **-35.374** | **3.197x10^-5^** |
| **0:1 - 2:1** | **-109.768** | **-135.936** | **-83.599** | **2.828x10^-13^** |
| **0:1 - 3:1** | **-153.819** | **-179.988** | **-127.650** | **1.110x10^-16^** |
| **1:1 - 2:1** | **-48.225** | **-74.394** | **-22.056** | **0.002** |
| **1:1 - 3:1** | **-92.277** | **-118.445** | **-66.108** | **3.820x10^-10^** |
| **2:1 - 3:1** | **-44.052** | **-70.221** | **-17.883** | **0.005** |
|  | **CBD Peak (visit included in model)** | | | |
| **0:1 - 1:1** | **-61.743** | **-87.989** | **-35.496** | **3.200x10^-5^** |
| **0:1 - 2:1** | **-110.067** | **-136.327** | **-83.808** | **2.964x10^-13^** |
| **0:1 - 3:1** | **-153.120** | **-179.480** | **-126.759** | **<2.22x10^-16^** |
| **1:1 - 2:1** | **-48.325** | **-74.564** | **-22.086** | **0.002** |
| **1:1 - 3:1** | **-91.377** | **-117.818** | **-64.936** | **8.214x10^-10^** |
| **2:1 - 3:1** | **-43.052** | **-69.541** | **-16.563** | **0.007** |
|  | **OH-THC AUC** |  |  |  |
| 0:1 - 1:1 | -1.329 | -3.456 | 0.798 | 0.591 |
| 0:1 - 2:1 | -0.940 | -3.067 | 1.187 | 0.810 |
| 0:1 - 3:1 | -1.695 | -3.822 | 0.432 | 0.379 |
| 1:1 - 2:1 | 0.389 | -1.738 | 2.516 | 0.983 |
| 1:1 - 3:1 | -0.366 | -2.493 | 1.761 | 0.986 |
| 2:1 - 3:1 | -0.754 | -2.881 | 1.372 | 0.891 |
|  | **OH-THC AUC (visit included in model)** |  |  |  |
| 0:1 - 1:1 | -1.368 | -3.490 | 0.753 | 0.565 |
| 0:1 - 2:1 | -0.999 | -3.122 | 1.123 | 0.779 |
| 0:1 - 3:1 | -1.557 | -3.687 | 0.574 | 0.458 |
| 1:1 - 2:1 | 0.369 | -1.752 | 2.490 | 0.985 |
| 1:1 - 3:1 | -0.189 | -2.326 | 1.949 | 0.998 |
| 2:1 - 3:1 | -0.557 | -2.698 | 1.584 | 0.953 |
|  | **OH-THC Peak** |  |  |  |
| 0:1 - 1:1 | -0.548 | -1.253 | 0.157 | 0.402 |
| 0:1 - 2:1 | -0.445 | -1.150 | 0.261 | 0.584 |
| 0:1 - 3:1 | -0.300 | -1.005 | 0.405 | 0.827 |
| 1:1 - 2:1 | 0.103 | -0.602 | 0.809 | 0.991 |
| 1:1 - 3:1 | 0.248 | -0.457 | 0.953 | 0.894 |
| 2:1 - 3:1 | 0.145 | -0.561 | 0.850 | 0.976 |
|  | **OH-THC Peak (visit included in model)** | | | |
| 0:1 - 1:1 | -0.548 | -1.256 | 0.160 | 0.406 |
| 0:1 - 2:1 | -0.445 | -1.154 | 0.264 | 0.587 |
| 0:1 - 3:1 | -0.299 | -1.011 | 0.412 | 0.832 |
| 1:1 - 2:1 | 0.103 | -0.605 | 0.811 | 0.991 |
| 1:1 - 3:1 | 0.249 | -0.465 | 0.963 | 0.896 |
| 2:1 - 3:1 | 0.146 | -0.569 | 0.861 | 0.976 |
|  | **COOH-THC AUC** |  |  |  |
| 0:1 - 1:1 | -5.913 | -17.845 | 6.020 | 0.751 |
| 0:1 - 2:1 | -12.670 | -24.602 | -0.737 | 0.146 |
| 0:1 - 3:1 | -10.249 | -22.181 | 1.684 | 0.312 |
| 1:1 - 2:1 | -6.757 | -18.689 | 5.176 | 0.665 |
| 1:1 - 3:1 | -4.336 | -16.268 | 7.596 | 0.884 |
| 2:1 - 3:1 | 2.421 | -9.512 | 14.353 | 0.977 |
|  | **COOH-THC AUC (visit included in model)** |  |  |  |
| 0:1 - 1:1 | -6.072 | -18.012 | 5.869 | 0.736 |
| 0:1 - 2:1 | -12.908 | -24.854 | -0.962 | 0.135 |
| 0:1 - 3:1 | -9.693 | -21.685 | 2.299 | 0.366 |
| 1:1 - 2:1 | -6.836 | -18.773 | 5.100 | 0.657 |
| 1:1 - 3:1 | -3.621 | -15.650 | 8.407 | 0.930 |
| 2:1 - 3:1 | 3.215 | -8.836 | 15.265 | 0.950 |
|  | **COOH-THC Peak** |  |  |  |
| 0:1 - 1:1 | -3.441 | -7.967 | 1.085 | 0.422 |
| 0:1 - 2:1 | -2.974 | -7.500 | 1.552 | 0.550 |
| 0:1 - 3:1 | -3.465 | -7.991 | 1.061 | 0.416 |
| 1:1 - 2:1 | 0.467 | -4.059 | 4.993 | 0.997 |
| 1:1 - 3:1 | -0.024 | -4.550 | 4.502 | 1.000 |
| 2:1 - 3:1 | -0.491 | -5.017 | 4.035 | 0.996 |
|  | **COOH-THC Peak (visit included in model)** | | | |
| 0:1 - 1:1 | -3.481 | -8.018 | 1.057 | 0.414 |
| 0:1 - 2:1 | -3.033 | -7.573 | 1.507 | 0.536 |
| 0:1 - 3:1 | -3.327 | -7.885 | 1.231 | 0.458 |
| 1:1 - 2:1 | 0.448 | -4.089 | 4.984 | 0.997 |
| 1:1 - 3:1 | 0.153 | -4.418 | 4.725 | 1.000 |
| 2:1 - 3:1 | -0.294 | -4.874 | 4.286 | 0.999 |
|  | **7-OH-CBD AUC** |  |  |  |
| 0:1 - 1:1 | -2.055 | -4.685 | 0.575 | 0.397 |
| **0:1 - 2:1** | **-4.676** | **-7.306** | **-2.046** | **0.003** |
| **0:1 - 3:1** | **-8.898** | **-11.528** | **-6.268** | **1.713x10^-9^** |
| 1:1 - 2:1 | -2.620 | -5.250 | 0.009 | 0.191 |
| **1:1 - 3:1** | **-6.843** | **-9.473** | **-4.213** | **3.572x10^-6^** |
| 2:1 - 3:1 | -4.222 | -6.852 | -1.592 | 0.008 |
|  | **7-OH-CBD AUC (visit included in model)** |  |  |  |
| 0:1 - 1:1 | -2.072 | -4.711 | 0.567 | 0.393 |
| **0:1 - 2:1** | **-4.701** | **-7.340** | **-2.061** | **0.003** |
| **0:1 - 3:1** | **-8.840** | **-11.490** | **-6.190** | **2.868x10^-9^** |
| 1:1 - 2:1 | -2.629 | -5.267 | 0.009 | 0.191 |
| **1:1 - 3:1** | **-6.768** | **-9.426** | **-4.110** | **5.942x10^-6^** |
| 2:1 - 3:1 | -4.140 | -6.803 | -1.477 | 0.011 |
|  | **7-OH-CBD Peak** |  |  |  |
| 0:1 - 1:1 | -0.480 | -1.708 | 0.748 | 0.86 |
| 0:1 - 2:1 | -0.912 | -2.140 | 0.316 | 0.443 |
| **0:1 - 3:1** | **-2.686** | **-3.914** | **-1.458** | **1.247x10^-4^** |
| 1:1 - 2:1 | -0.432 | -1.660 | 0.796 | 0.894 |
| **1:1 - 3:1** | **-2.206** | **-3.434** | **-0.978** | **0.002** |
| 2:1 - 3:1 | -1.774 | -3.002 | -0.546 | 0.022 |
|  | **7-OH-CBD Peak (visit included in model)** | | | |
| 0:1 - 1:1 | -0.459 | -1.684 | 0.767 | 0.875 |
| 0:1 - 2:1 | -0.879 | -2.106 | 0.347 | 0.474 |
| **0:1 - 3:1** | **-2.761** | **-3.992** | **-1.530** | **7.960x10^-5^** |
| 1:1 - 2:1 | -0.421 | -1.646 | 0.804 | 0.900 |
| **1:1 - 3:1** | **-2.302** | **-3.537** | **-1.068** | **0.001** |
| 2:1 - 3:1 | -1.882 | -3.119 | -0.645 | 0.014 |

**eTable 5** Logarithmic concentrations of THC and CBD over time, with intercept and slope across ratios

| PK Concentration (log) over time | | | | |
| --- | --- | --- | --- | --- |
| Intercept and slope of concentration (log) from peak over time | | | | |
| Ratio | Intercept | | Slope | |
|  | Mean | SD | Mean | SD |
| THC | | | | |
| 0:1 | 2.56 | 0.34 | -0.37 | 0.08 |
| 1:1 | 2.59 | 0.32 | -0.36 | 0.07 |
| 2:1 | 2.48 | 0.39 | -0.33 | 0.08 |
| 3:1 | 2.48 | 0.55 | -0.34 | 0.13 |
| CBD | | | | |
| 0:1 | -0.27 | 0.20 | -0.01 | 0.04 |
| 1:1 | 2.28 | 0.36 | -0.28 | 0.08 |
| 2:1 | 2.50 | 0.40 | -0.26 | 0.08 |
| 3:1 | 2.59 | 0.64 | -0.24 | 0.15 |

**eFigure 3** Logarithmic concentrations of THC and CBD over time


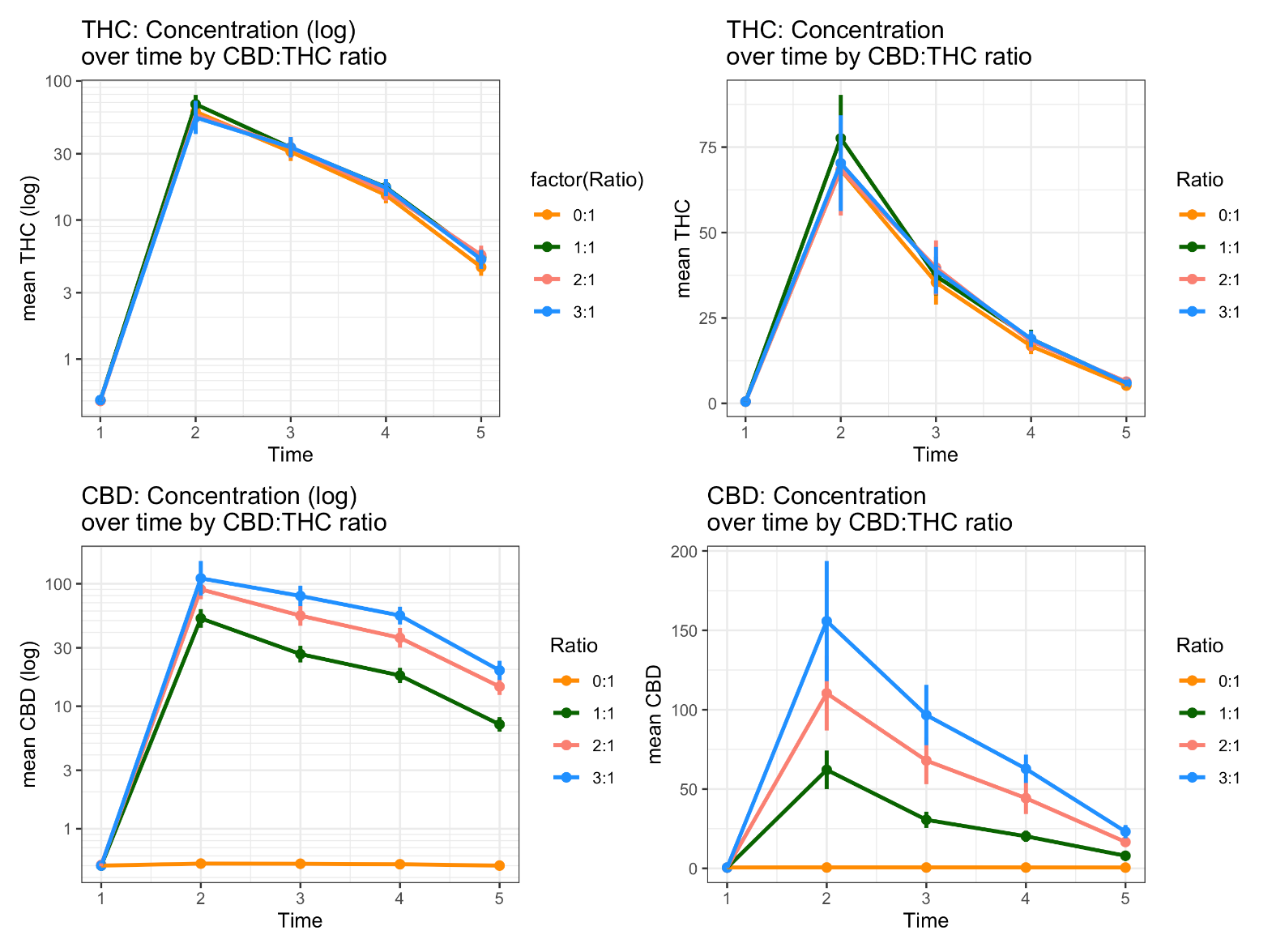


**eFigure 4** Hopkins Verbal Learning Task – Revised performance, stratified by CBD:THC ratios. Circles show individual data points, diamonds show mean values, boxplots show median and interquartile range and half violin plots show distribution of participant scores.

**A** Immediate repetitions **B** Delayed repetitions **C** Immediate intrusions **D** Delayed intrusions


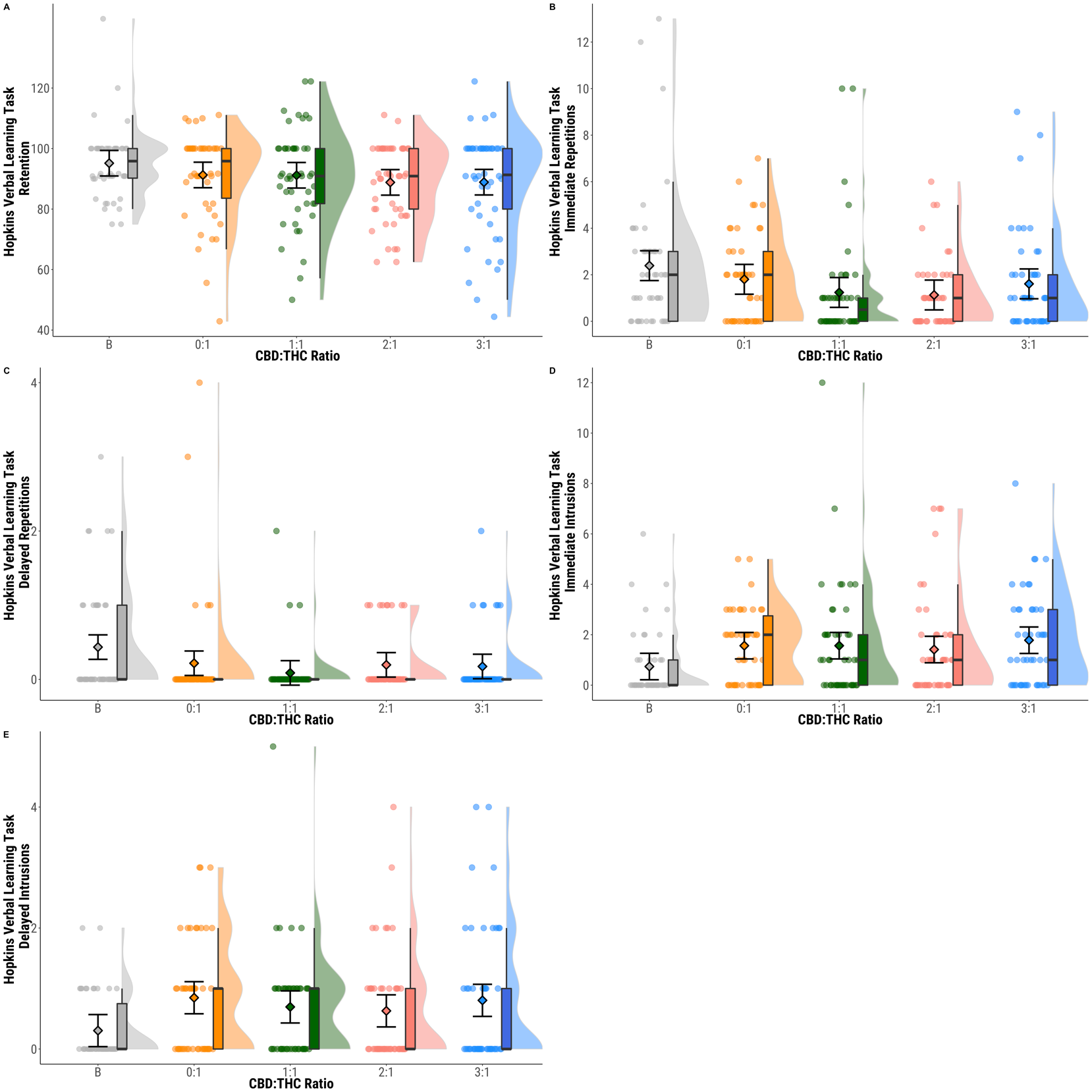


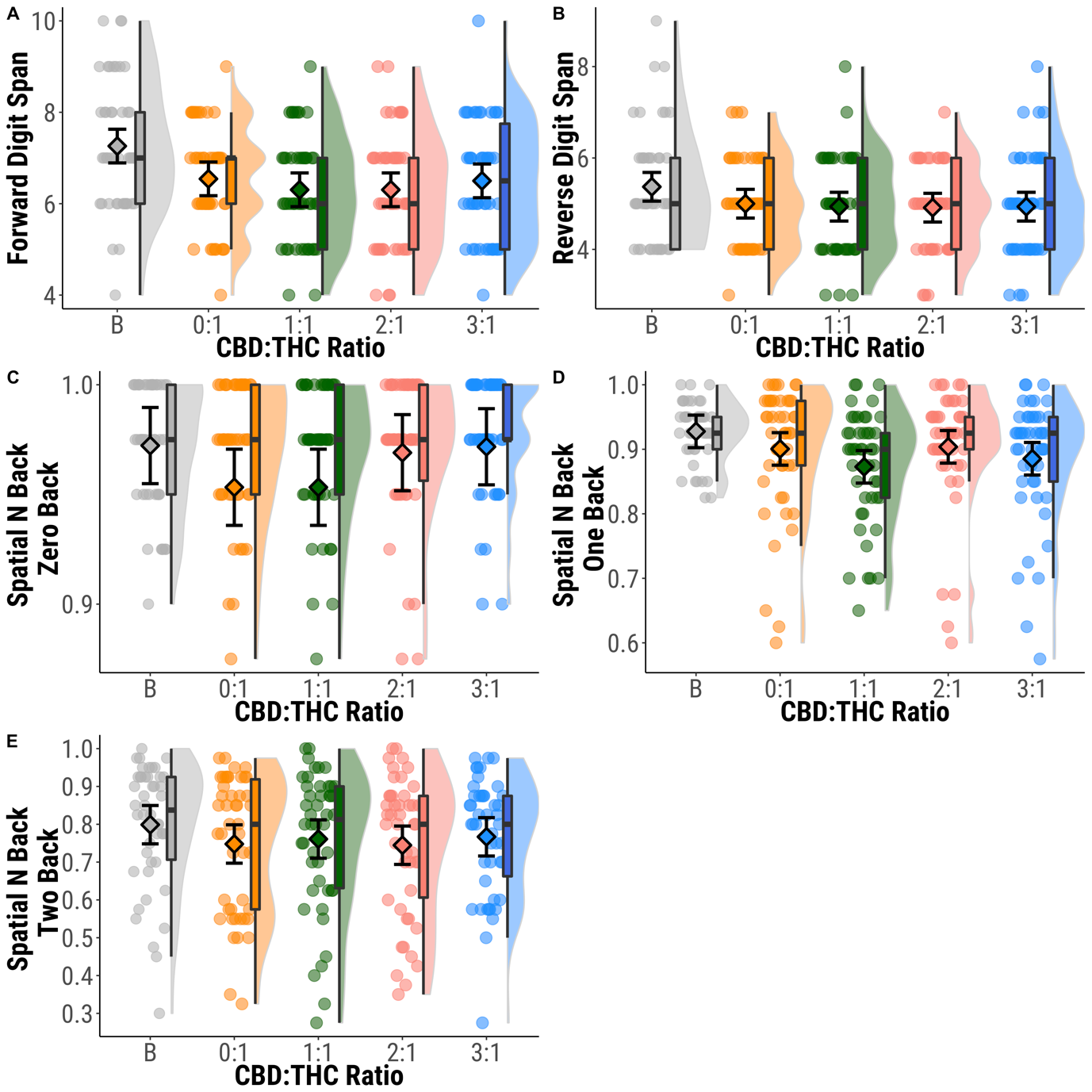


**eFigure 5** Digit span and Spatial N-back performance stratified by CBD:THC ratios. Circles show individual data points, diamonds show mean values, boxplots show median and interquartile range and half violin plots show distribution of participant scores.

**A** Forward digit span **B** Reverse digit span **C** Spatial N-back zero back **D** Spatial N-back one back **E** Spatial N-back two back

| **eTable 6** Hopkins verbal learning task – revised (HVLT), digit span and spatial N-back (SNB) performance stratified by CBD:THC ratio. EMM, estimated marginal mean; SE, standard error | | | | | | | | | |
| --- | --- | --- | --- | --- | --- | --- | --- | --- | --- |
|  |  | **CBD:THC Ratio** | | | | | |  | |
|  |  | **Baseline** | **0:1** | | **1:1** | **2:1** | **3:1** | **Significant impairment from baseline?** | **Significant difference between CBD:THC ratios?** |
| **Outcome** |  | |  |  | | |  |  | |
| **HVLT - Immediate Recall** | **EMM (SE)** | 29.3 (0.647) | 26.1 (0.647) | | 25.7 (0.647) | 25.1 (0.647) | 26.4 (0.647) | Yes  t(45)=5·365, p=2·71x10^-6^ | No |
| **HVLT - Delayed Recall** | **EMM (SE)** | 10.50 (0.314) | 9.41 (0.314) | | 9.26 (0.314) | 9.09 (0.314) | 9.39 (0.314) | Yes  t(45)=3·399, p=0·001 | No |
| **HVLT - Retention** | **EMM (SE)** | 95.2 (2.14) | 91.3 (2.14) | | 91.2 (2.14) | 88.8 (2.14) | 88.9 (2.14) | No | No |
| **HVLT - Immediate Repetitions** | **EMM (SE)** | 2.39 (0.325) | 1.80 (0.325) | | 1.24 (0.325) | 1.13 (0.325) | 1.61 (0.325) | No | No |
| **HVLT - Delayed Repetitions** | **EMM (SE)** | 0.44 (0.084) | 0.22 (0.084) | | 0.09 (0.084) | 0.20 (0.084) | 0.17 (0.084) | No | No |
| **HVLT - Immediate Intrusions** | **EMM (SE)** | 0.74 (0.266) | 1.57 (0.266) | | 1.57 (0.266) | 1.41 (0.266) | 1.78 (0.266) | Yes  t(45)=-3·824, p=4·02x10^-4^ | No |
| **HVLT - Delayed Intrusions** | **EMM (SE)** | 0.30 (0.135) | 0.85 (0.135) | | 0.70 (0.135) | 0.63 (0.135) | 0.80 (0.135) | Yes  t(45)=-3·322, p=0·002 | No |
| **Forward Digit Span** | **EMM (SE)** | 7.26 (0.186) | 6.54 (0.186) | | 6.30 (0.186) | 6.30 (0.186) | 6.50 (0.186) | Yes  t(45)=3·309, p=0·002 | No |
| **Reverse Digit Span** | **EMM (SE)** | 5.47 (0.159) | 5.00 (0.159) | | 4.93 (0.159) | 4.91 (0.159) | 4.93 (0.159) | No | No |
| **SNB – 0 Back** | **EMM (SE)** | 0.972 (0.004) | 0.969 (0.004) | | 0.968 (0.004) | 0.971 (0.004) | 0.977 (0.004) | No | No |
| **SNB – 1 Back** | **EMM (SE)** | 0.927 (0.013) | 0.901 (0.013) | | 0.873 (0.013) | 0.904 (0.013) | 0.885 (0.013) | No | No |
| **SNB – 2 Back** | **EMM (SE)** | 0.799 (0.026) | 0.748 (0.026) | | 0.761 (0.026) | 0.745 (0.026) | 0.767 (0.026) | No | No |

| **eTable 7** Results of linear mixed models of cognitive outcomes for each contrast between CBD:THC ratios. Statistically significant rows are presented in bold. CI, confidence interval; EMM, estimated marginal mean; HVLT, Hopkins Verbal Learning Task – revised; SNB, spatial N-back | | | | |
| --- | --- | --- | --- | --- |
|  |  | | | |
| **Contrast** | **EMM difference** | **Lower 95% CI** | **Upper 95% CI** | **p-value** |
|  | **HVLT - Immediate recall** |  |  |  |
| 0:1 - 1:1 | 0.565 | -0.736 | 1.823 | 0.811 |
| 0:1 - 2:1 | 1.152 | -0.149 | 2.410 | 0.273 |
| 0:1 - 3:1 | -0.174 | -1.475 | 1.084 | 0.993 |
| 1:1 - 2:1 | 0.587 | -0.692 | 1.866 | 0.793 |
| 1:1 - 3:1 | -0.739 | -2.018 | 0.540 | 0.652 |
| 2:1 - 3:1 | -1.326 | -2.605 | -0.047 | 0.164 |
|  | **HVLT - Immediate recall (visit included in model)** | | | |
| 0:1 - 1:1 | 0.519 | -0.754 | 1.747 | 0.839 |
| 0:1 - 2:1 | 1.082 | -0.191 | 2.312 | 0.308 |
| 0:1 - 3:1 | -0.011 | -1.288 | 1.224 | 1.000 |
| 1:1 - 2:1 | 0.564 | -0.687 | 1.814 | 0.802 |
| 1:1 - 3:1 | -0.530 | -1.789 | 0.731 | 0.833 |
| 2:1 - 3:1 | -1.093 | -2.355 | 0.170 | 0.307 |
|  | **HVLT – Delayed recall** |  |  |  |
| 0:1 - 1:1 | 0.152 | -0.522 | 0.827 | 0.969 |
| 0:1 - 2:1 | 0.326 | -0.348 | 1.001 | 0.765 |
| 0:1 - 3:1 | 0.022 | -0.653 | 0.696 | 1.000 |
| 1:1 - 2:1 | 0.174 | -0.501 | 0.848 | 0.954 |
| 1:1 - 3:1 | -0.130 | -0.805 | 0.544 | 0.980 |
| 2:1 - 3:1 | -0.304 | -0.979 | 0.370 | 0.800 |
|  | **HVLT - Delayed recall (visit included in model)** | | | |
| 0:1 - 1:1 | 0.143 | -0.532 | 0.818 | 0.974 |
| 0:1 - 2:1 | 0.312 | -0.363 | 0.987 | 0.788 |
| 0:1 - 3:1 | 0.054 | -0.624 | 0.732 | 0.999 |
| 1:1 - 2:1 | 0.169 | -0.505 | 0.844 | 0.958 |
| 1:1 - 3:1 | -0.089 | -0.769 | 0.591 | 0.994 |
| 2:1 - 3:1 | -0.258 | -0.939 | 0.423 | 0.870 |
|  | **HVLT - Retention** |  |  |  |
| 0:1 - 1:1 | 0.098 | -5.470 | 5.666 | 1.000 |
| 0:1 - 2:1 | 2.448 | -3.120 | 8.016 | 0.812 |
| 0:1 - 3:1 | 2.383 | -3.185 | 7.950 | 0.824 |
| 1:1 - 2:1 | 2.350 | -3.218 | 7.918 | 0.830 |
| 1:1 - 3:1 | 2.285 | -3.283 | 7.852 | 0.842 |
| 2:1 - 3:1 | -0.065 | -5.633 | 5.502 | 1.000 |
|  | **HVLT - Retention (visit included in model)** | | | |
| 0:1 - 1:1 | 0.132 | -5.454 | 5.719 | 1.000 |
| 0:1 - 2:1 | 2.500 | -3.089 | 8.089 | 0.804 |
| 0:1 - 3:1 | 2.262 | -3.349 | 7.872 | 0.849 |
| 1:1 - 2:1 | 2.367 | -3.218 | 7.952 | 0.829 |
| 1:1 - 3:1 | 2.129 | -3.499 | 7.757 | 0.871 |
| 2:1 - 3:1 | -0.238 | -5.876 | 5.400 | 1.000 |

|  |  | | | |
| --- | --- | --- | --- | --- |
|  | **HVLT - Immediate Repetitions** | | | |
| 0:1 - 1:1 | 0.565 | -0.083 | 1.213 | 0.299 |
| 0:1 - 2:1 | 0.674 | 0.026 | 1.322 | 0.160 |
| 0:1 - 3:1 | 0.196 | -0.452 | 0.844 | 0.929 |
| 1:1 - 2:1 | 0.109 | -0.539 | 0.757 | 0.987 |
| 1:1 - 3:1 | -0.370 | -1.017 | 0.278 | 0.660 |
| 2:1 - 3:1 | -0.478 | -1.126 | 0.170 | 0.448 |
|  | **HVLT - Immediate Repetitions (visit included in model)** | | | |
| 0:1 - 1:1 | 0.556 | -0.092 | 1.203 | 0.314 |
| 0:1 - 2:1 | 0.659 | 0.011 | 1.307 | 0.175 |
| 0:1 - 3:1 | 0.230 | -0.421 | 0.880 | 0.893 |
| 1:1 - 2:1 | 0.104 | -0.544 | 0.751 | 0.988 |
| 1:1 - 3:1 | -0.326 | -0.979 | 0.327 | 0.746 |
| 2:1 - 3:1 | -0.430 | -1.084 | 0.224 | 0.549 |
|  | **HVLT - Delayed Repetitions** | | | |
| 0:1 - 1:1 | 0.130 | -0.075 | 0.336 | 0.578 |
| 0:1 - 2:1 | 0.022 | -0.184 | 0.227 | 0.997 |
| 0:1 - 3:1 | 0.043 | -0.162 | 0.249 | 0.974 |
| 1:1 - 2:1 | -0.109 | -0.314 | 0.097 | 0.711 |
| 1:1 - 3:1 | -0.087 | -0.292 | 0.118 | 0.829 |
| 2:1 - 3:1 | 0.022 | -0.184 | 0.227 | 0.997 |
|  | **HVLT - Delayed Repetitions (visit included in model)** | | | |
| 0:1 - 1:1 | 0.131 | -0.076 | 0.337 | 0.579 |
| 0:1 - 2:1 | 0.022 | -0.184 | 0.229 | 0.996 |
| 0:1 - 3:1 | 0.042 | -0.165 | 0.250 | 0.976 |
| 1:1 - 2:1 | -0.109 | -0.315 | 0.098 | 0.714 |
| 1:1 - 3:1 | -0.088 | -0.296 | 0.120 | 0.828 |
| 2:1 - 3:1 | 0.020 | -0.188 | 0.228 | 0.997 |
|  | **HVLT - Immediate Intrusions** | | | |
| 0:1 - 1:1 | 0.000 | -0.700 | 0.700 | 1.000 |
| 0:1 - 2:1 | 0.152 | -0.548 | 0.852 | 0.972 |
| 0:1 - 3:1 | -0.217 | -0.917 | 0.483 | 0.924 |
| 1:1 - 2:1 | 0.152 | -0.548 | 0.852 | 0.972 |
| 1:1 - 3:1 | -0.217 | -0.917 | 0.483 | 0.924 |
| 2:1 - 3:1 | -0.370 | -1.070 | 0.330 | 0.712 |
|  | **HVLT - Immediate Intrusions (visit included in model)** | | | |
| 0:1 - 1:1 | -0.005 | -0.707 | 0.697 | 1.000 |
| 0:1 - 2:1 | 0.144 | -0.558 | 0.847 | 0.976 |
| 0:1 - 3:1 | -0.199 | -0.904 | 0.506 | 0.941 |
| 1:1 - 2:1 | 0.150 | -0.552 | 0.851 | 0.973 |
| 1:1 - 3:1 | -0.194 | -0.901 | 0.514 | 0.946 |
| 2:1 - 3:1 | -0.343 | -1.052 | 0.365 | 0.764 |
|  | **HVLT - Delayed Intrusions** | | | |
| 0:1 - 1:1 | 0.152 | -0.213 | 0.517 | 0.835 |
| 0:1 - 2:1 | 0.217 | -0.147 | 0.582 | 0.628 |
| 0:1 - 3:1 | 0.043 | -0.321 | 0.408 | 0.995 |
| 1:1 - 2:1 | 0.065 | -0.300 | 0.430 | 0.984 |
| 1:1 - 3:1 | -0.109 | -0.474 | 0.256 | 0.932 |
| 2:1 - 3:1 | -0.174 | -0.539 | 0.191 | 0.772 |
|  | **HVLT - Delayed Intrusions (visit included in model)** | | | |
| 0:1 - 1:1 | 0.153 | -0.214 | 0.519 | 0.836 |
| 0:1 - 2:1 | 0.218 | -0.148 | 0.585 | 0.629 |
| 0:1 - 3:1 | 0.042 | -0.326 | 0.410 | 0.996 |
| 1:1 - 2:1 | 0.065 | -0.301 | 0.432 | 0.984 |
| 1:1 - 3:1 | -0.111 | -0.480 | 0.258 | 0.930 |
| 2:1 - 3:1 | -0.176 | -0.546 | 0.193 | 0.772 |

|  | **Forward Digit Span** | | | |
| --- | --- | --- | --- | --- |
| 0:1 - 1:1 | 0.239 | -0.142 | 0.620 | 0.588 |
| 0:1 - 2:1 | 0.239 | -0.142 | 0.620 | 0.588 |
| 0:1 - 3:1 | 0.043 | -0.338 | 0.425 | 0.996 |
| 1:1 - 2:1 | 0.000 | -0.381 | 0.381 | 1.000 |
| 1:1 - 3:1 | -0.196 | -0.577 | 0.186 | 0.730 |
| 2:1 - 3:1 | -0.196 | -0.577 | 0.186 | 0.730 |
|  | **Forward Digit Span (visit included in model)** | | | |
| 0:1 - 1:1 | 0.252 | -0.121 | 0.626 | 0.526 |
| 0:1 - 2:1 | 0.259 | -0.115 | 0.633 | 0.505 |
| 0:1 - 3:1 | -0.003 | -0.378 | 0.372 | 1.000 |
| 1:1 - 2:1 | 0.007 | -0.367 | 0.380 | 1.000 |
| 1:1 - 3:1 | -0.255 | -0.632 | 0.121 | 0.523 |
| 2:1 - 3:1 | -0.262 | -0.639 | 0.115 | 0.502 |
|  | **Reverse Digit Span** | | | |
| 0:1 - 1:1 | 0.065 | -0.289 | 0.419 | 0.982 |
| 0:1 - 2:1 | 0.087 | -0.267 | 0.441 | 0.960 |
| 0:1 - 3:1 | 0.065 | -0.289 | 0.419 | 0.982 |
| 1:1 - 2:1 | 0.022 | -0.332 | 0.376 | 0.999 |
| 1:1 - 3:1 | 0.000 | -0.354 | 0.354 | 1.000 |
| 2:1 - 3:1 | -0.022 | -0.376 | 0.332 | 0.999 |
|  | **Reverse Digit Span (visit included in model)** | | | |
| 0:1 - 1:1 | 0.058 | -0.294 | 0.409 | 0.988 |
| 0:1 - 2:1 | 0.075 | -0.277 | 0.428 | 0.973 |
| 0:1 - 3:1 | 0.092 | -0.261 | 0.446 | 0.953 |
| 1:1 - 2:1 | 0.018 | -0.334 | 0.370 | 1.000 |
| 1:1 - 3:1 | 0.035 | -0.320 | 0.389 | 0.997 |
| 2:1 - 3:1 | 0.017 | -0.338 | 0.372 | 1.000 |
|  | **SNB - 0 Back** | | | |
| 0:1 - 1:1 | -0.001 | -0.023 | 0.022 | 1.000 |
| 0:1 - 2:1 | -0.016 | -0.039 | 0.006 | 0.454 |
| 0:1 - 3:1 | -0.019 | -0.041 | 0.003 | 0.315 |
| 1:1 - 2:1 | -0.016 | -0.038 | 0.006 | 0.484 |
| 1:1 - 3:1 | -0.018 | -0.041 | 0.004 | 0.341 |
| 2:1 - 3:1 | -0.003 | -0.025 | 0.019 | 0.995 |
|  | **SNB - 0 Back (visit included in model)** | | | |
| 0:1 - 1:1 | -0.001 | -0.023 | 0.022 | 1.000 |
| 0:1 - 2:1 | -0.017 | -0.039 | 0.006 | 0.443 |
| 0:1 - 3:1 | -0.018 | -0.041 | 0.004 | 0.349 |
| 1:1 - 2:1 | -0.016 | -0.038 | 0.006 | 0.482 |
| 1:1 - 3:1 | -0.018 | -0.040 | 0.005 | 0.387 |
| 2:1 - 3:1 | -0.002 | -0.024 | 0.021 | 0.998 |
|  | **SNB - 1 Back** | | | |
| 0:1 - 1:1 | 0.023 | -0.017 | 0.064 | 0.646 |
| 0:1 - 2:1 | -0.013 | -0.053 | 0.027 | 0.914 |
| 0:1 - 3:1 | 0.010 | -0.030 | 0.051 | 0.955 |
| 1:1 - 2:1 | -0.036 | -0.077 | 0.004 | 0.266 |
| 1:1 - 3:1 | -0.013 | -0.053 | 0.027 | 0.914 |
| 2:1 - 3:1 | 0.023 | -0.017 | 0.064 | 0.646 |
|  | **SNB - 1 Back (visit included in model)** | | | |
| 0:1 - 1:1 | 0.022 | -0.017 | 0.062 | 0.671 |
| 0:1 - 2:1 | -0.014 | -0.054 | 0.026 | 0.886 |
| **0:1 - 3:1** | 0.014 | -0.027 | 0.054 | 0.904 |
| 1:1 - 2:1 | -0.037 | -0.077 | 0.003 | 0.250 |
| 1:1 - 3:1 | -0.009 | -0.049 | 0.031 | 0.971 |
| 2:1 - 3:1 | 0.028 | -0.012 | 0.068 | 0.501 |

|  | **SNB - 2 Back** | | | |
| --- | --- | --- | --- | --- |
| 0:1 - 1:1 | 0.023 | -0.053 | 0.027 | 0.646 |
| 0:1 - 2:1 | -0.013 | -0.037 | 0.044 | 0.914 |
| 0:1 - 3:1 | 0.010 | -0.059 | 0.021 | 0.955 |
| 1:1 - 2:1 | -0.036 | -0.024 | 0.057 | 0.266 |
| 1:1 - 3:1 | -0.013 | -0.046 | 0.034 | 0.914 |
| 2:1 - 3:1 | 0.023 | -0.063 | 0.018 | 0.646 |
|  | **SNB - 2 Back (visit included in model)** | | | |
| 0:1 - 1:1 | -0.014 | -0.054 | 0.026 | 0.898 |
| 0:1 - 2:1 | 0.002 | -0.038 | 0.042 | 1.000 |
| 0:1 - 3:1 | -0.016 | -0.056 | 0.024 | 0.854 |
| 1:1 - 2:1 | 0.016 | -0.024 | 0.056 | 0.856 |
| 1:1 - 3:1 | -0.002 | -0.043 | 0.038 | 1.000 |
| 2:1 - 3:1 | -0.018 | -0.059 | 0.022 | 0.807 |

**eFigure 6** Box and whisker plots for individual slopes and distribution of intercepts for delayed recall on the HVLT-R. Effect size for slope: -0.036 (95% CI: -0.31, 0.32).


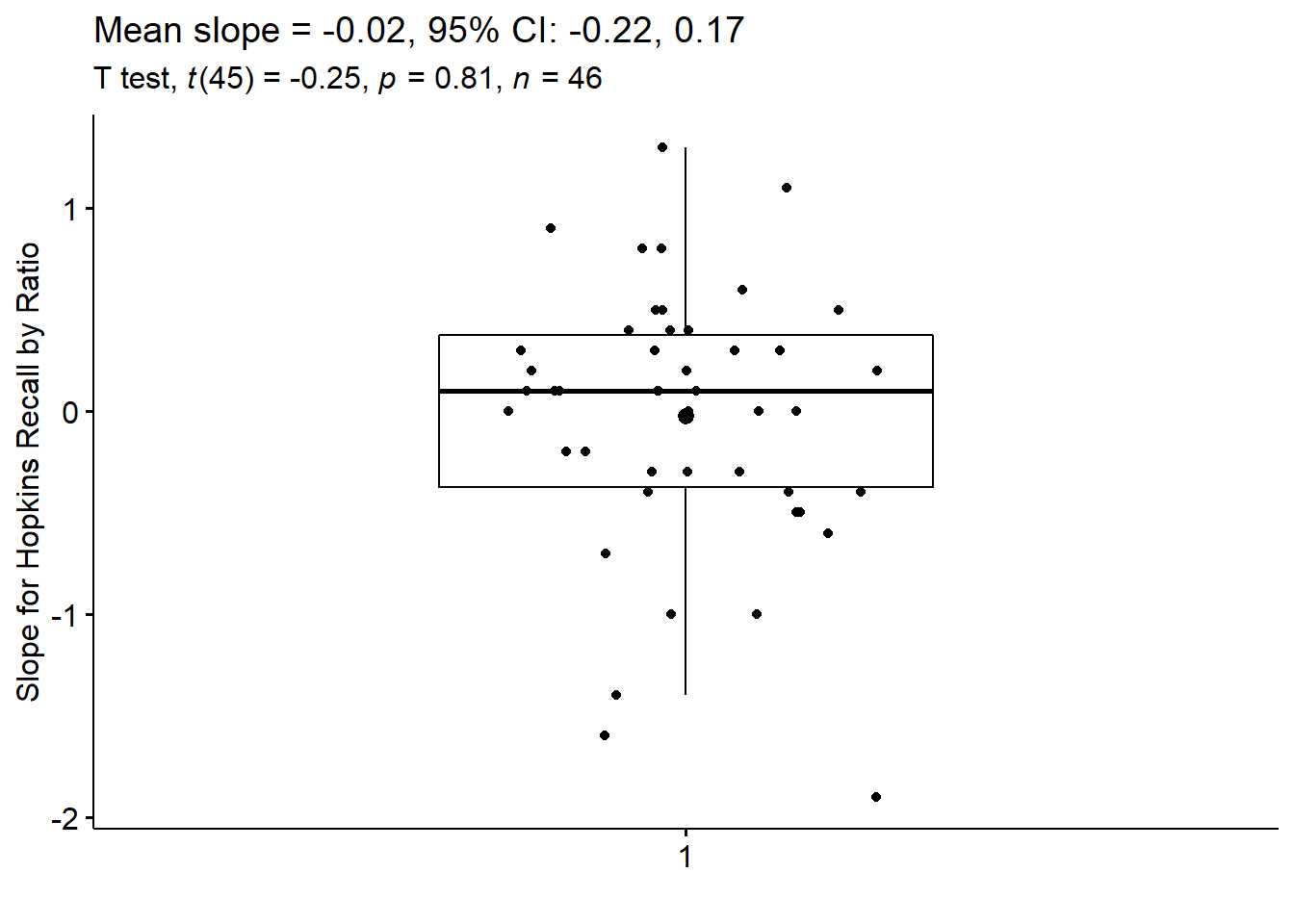

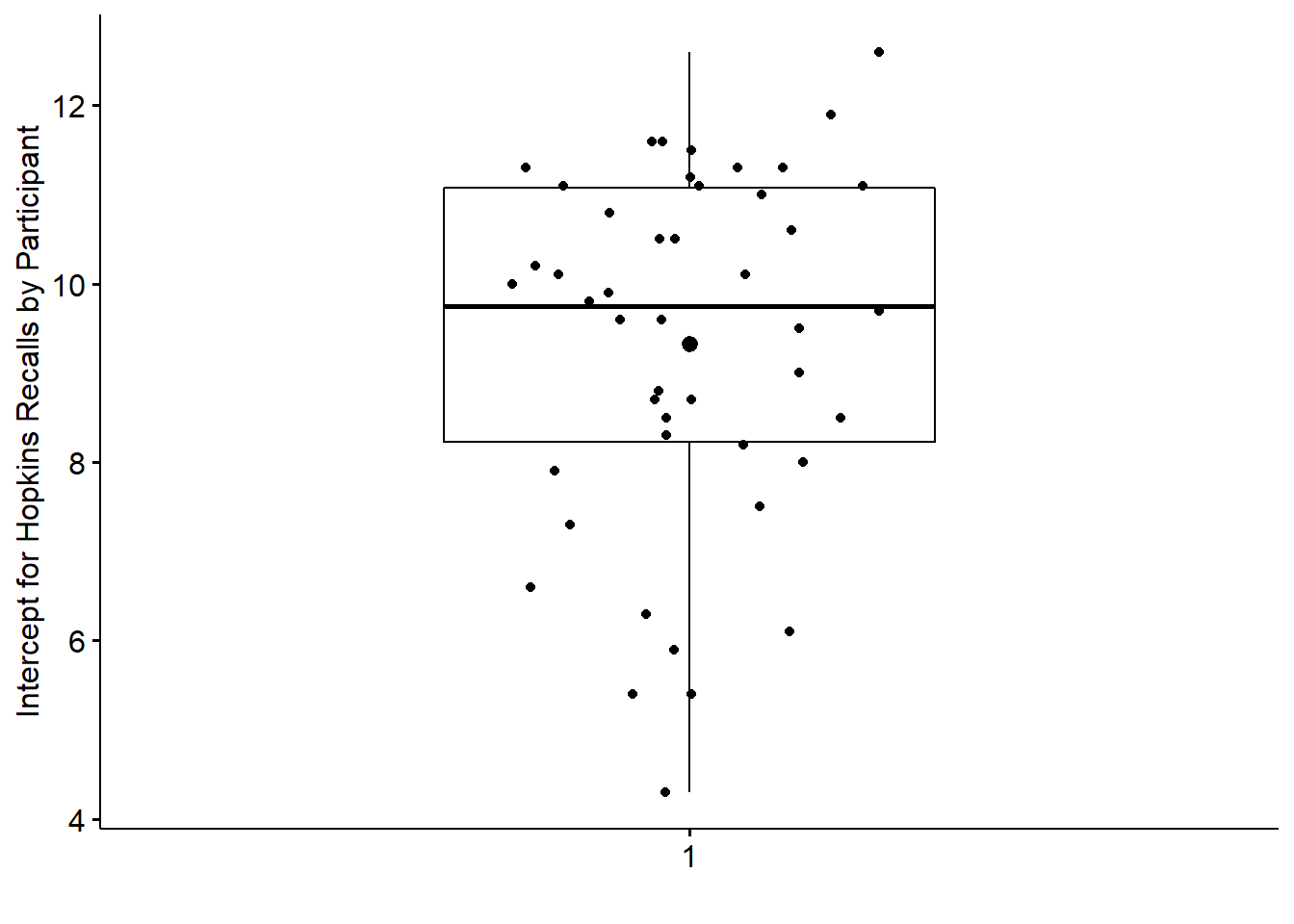


**eTable 8** Effect sizes across ratio contrasts (Cohen’s d and 95% CI) for delayed recall on the HVLT-R

| **Contrast** | **Cohens_d** | **CI_low** | **CI_high** |
| --- | --- | --- | --- |
| 0:1 - 1:1 | -0.07 | -0.36 | 0.23 |
| 0:1 - 2:1 | -0.17 | -0.46 | 0.13 |
| 0:1 - 3:1 | -0.01 | -0.30 | 0.28 |
| 1:1 - 2:1 | -0.07 | -0.37 | 0.22 |
| 1:1 - 3:1 | 0.05 | -0.24 | 0.34 |
| 2:1 - 3:1 | 0.13 | -0.16 | 0.42 |


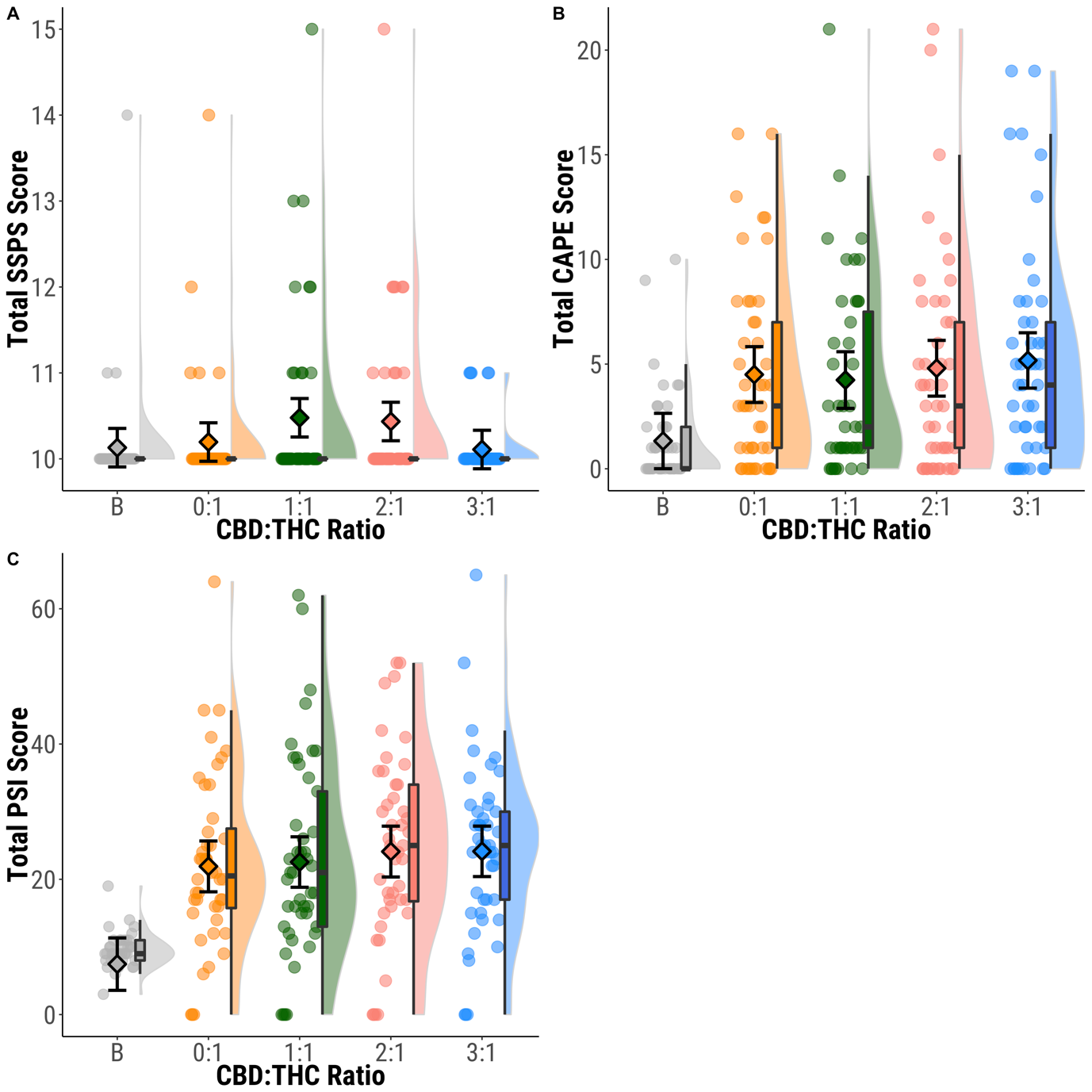


**eFigure 7** Psychological effects stratified by CBD:THC ratios. Circles show individual data points, diamonds show mean values, boxplots show median and interquartile range and half violin plots show distribution of participant scores.

**A** State Social Paranoia Scale (SSPS) **B** Community Assessment of Psychic Experiences (CAPE) **C** Psychomimetic States Inventory (PSI)

|  |  | **CBD:THC Ratio** | | | | | |  |
| --- | --- | --- | --- | --- | --- | --- | --- | --- |
|  |  | **Baseline** | **0:1** | | **1:1** | **2:1** | **3:1** | **Significant impairment from baseline?** |
| **Outcome** |  | |  |  | | |  |  |
| **PANSS** | **EMM (SE)** | 7.04 (0.317) | 8.96 (0.317) | | 8.61 (0.317) | 9.35 (0.317) | 8.70 (0.317) | Yes: t(45)=-4·709, d=0.69, p=2.41x10^-5^ |
| **SSPS** | **EMM (SE)** | 10.1 (0.116) | 10.2 (0.116) | | 10.5 (0.116) | 10.5 (0.116) | 10.1 (0.116) | No: t(45)=-1·096, d=0.16, p=0.279 |
| **CAPE** | **EMM (SE)** | 1.33 (0.669) | 4.50 (0.674) | | 4.24 (0.684) | 4.80 (0.674) | 5.17 (0.669) | Yes: t(45)=-4.088, d=1.18, p=0.0002 |
| **PSI** | **EMM (SE)** | 7.46 (1.96) | 21.91 (1.90) | | 22.5 (1.88) | 24.1 (1.90) | 24.1 (1.88) | Yes: t(39)=-7.461, d=0.60, p=5.025x10^-9^ |

**eTable 9** Results of linear mixed models of psychological outcomes for each contrast between CBD:THC ratios. Statistically significant rows are presented in bold. CI, confidence interval; EMM, estimated marginal mean; PANSS, Positive and Negative Syndrome Scale; SSPS, State Social Paranoia Scale; CAPE, Community Assessment of Psychic Experiences; PSI, Psychomimetic States Inventory

| **eTable 10** Results of linear mixed models of psychological measures for each contrast between CBD:THC ratios. Statistically significant rows are presented in bold. CI, confidence interval; EMM, estimated marginal mean. | | | | |
| --- | --- | --- | --- | --- |
|  |  | | | |
| **Contrast** | **EMM difference** | **Lower 95% CI** | **Upper 95% CI** | **p-value** |
|  | **PANSS** |  |  |  |
| 0:1 - 1:1 | -0.658 | -1.382 | 0.066 | 0.264 |
| 0:1 - 2:1 | -0.087 | -0.806 | 0.632 | 0.995 |
| 0:1 - 3:1 | -0.105 | -0.828 | 0.619 | 0.991 |
| 1:1 - 2:1 | 0.571 | -0.153 | 1.295 | 0.389 |
| 1:1 - 3:1 | 0.553 | -0.175 | 1.282 | 0.423 |
| 2:1 - 3:1 | -0.018 | -0.741 | 0.706 | 1.000 |
|  | **PANSS (visit included in model)** | | | |
| 0:1 - 1:1 | -0.657 | -1.381 | 0.067 | 0.265 |
| 0:1 - 2:1 | -0.124 | -0.849 | 0.601 | 0.986 |
| 0:1 - 3:1 | -0.114 | -0.839 | 0.610 | 0.989 |
| 1:1 - 2:1 | 0.533 | -0.197 | 1.263 | 0.458 |
| 1:1 - 3:1 | 0.543 | -0.186 | 1.273 | 0.441 |
| 2:1 - 3:1 | 0.010 | -0.717 | 0.738 | 1.000 |
|  | **SSPS** |  |  |  |
| 0:1 - 1:1 | -0.283 | -0.599 | 0.077 | 0.326 |
| 0:1 - 2:1 | -0.239 | -0.555 | 0.121 | 0.476 |
| 0:1 - 3:1 | 0.087 | -0.229 | 0.447 | 0.953 |
| 1:1 - 2:1 | 0.043 | -0.295 | 0.382 | 0.994 |
| 1:1 - 3:1 | 0.370 | 0.032 | 0.708 | 0.121 |
| 2:1 - 3:1 | 0.326 | -0.012 | 0.664 | 0.207 |
|  | **SSPS (visit included in model)** | | | |
| 0:1 - 1:1 | -0.292 | -0.605 | 0.064 | 0.285 |
| 0:1 - 2:1 | -0.254 | -0.566 | 0.103 | 0.412 |
| 0:1 - 3:1 | 0.121 | -0.193 | 0.478 | 0.882 |
| 1:1 - 2:1 | 0.039 | -0.295 | 0.373 | 0.995 |
| 1:1 - 3:1 | 0.413 | 0.076 | 0.749 | 0.064 |
| 2:1 - 3:1 | 0.375 | 0.037 | 0.711 | 0.111 |
|  | **CAPE** |  |  |  |
| 0:1 - 1:1 | 0.235 | -1.208 | 1.678 | 0.988 |
| 0:1 - 2:1 | -0.344 | -1.771 | 1.084 | 0.962 |
| 0:1 - 3:1 | -0.651 | -2.069 | 0.766 | 0.792 |
| 1:1 - 2:1 | -0.579 | -2.027 | 0.870 | 0.852 |
| 1:1 - 3:1 | -0.886 | -2.325 | 0.552 | 0.602 |
| 2:1 - 3:1 | -0.307 | -1.724 | 1.109 | 0.972 |
|  | **CAPE (visit included in model)** | | | |
| 0:1 - 1:1 | 0.249 | -1.195 | 1.694 | 0.986 |
| 0:1 - 2:1 | -0.328 | -1.757 | 1.100 | 0.967 |
| 0:1 - 3:1 | -0.713 | -2.138 | 0.713 | 0.746 |
| 1:1 - 2:1 | -0.577 | -2.027 | 0.872 | 0.853 |
| 1:1 - 3:1 | -0.962 | -2.412 | 0.488 | 0.542 |
| 2:1 - 3:1 | -0.385 | -1.813 | 1.044 | 0.948 |
|  | **PSI** |  |  |  |
| 0:1 - 1:1 | -2.110 | -7.735 | 3.515 | 0.874 |
| 0:1 - 2:1 | -2.238 | -7.750 | 3.274 | 0.846 |
| 0:1 - 3:1 | 0.732 | -4.621 | 6.085 | 0.993 |
| 1:1 - 2:1 | -0.129 | -5.563 | 5.305 | 1.000 |
| 1:1 - 3:1 | 2.841 | -2.415 | 8.098 | 0.697 |
| 2:1 - 3:1 | 2.970 | -2.099 | 8.039 | 0.641 |

|  | **PSI (visit included in model)** | | | |
| --- | --- | --- | --- | --- |
| 0:1 - 1:1 | -2.107 | -7.775 | 3.560 | 0.877 |
| 0:1 - 2:1 | -2.235 | -7.789 | 3.319 | 0.849 |
| 0:1 - 3:1 | 0.731 | -4.747 | 6.208 | 0.993 |
| 1:1 - 2:1 | -0.128 | -5.598 | 5.343 | 1.000 |
| 1:1 - 3:1 | 2.838 | -2.511 | 8.186 | 0.710 |
| 2:1 - 3:1 | 2.965 | -2.192 | 8.122 | 0.655 |

**eFigure 8** Subjective drug effects, as measured by visual analogues scales (VAS), at each time point, stratified by CBD:THC ratios. Circles show individual data points, diamonds show mean values and boxplots show median and interquartile range.

**A** Drug effects pleasurable **B** Dry mouth **C** Enhanced colour perception **D** Enhanced sound perception **E** Feel anxious **F** Feel calm and relaxed **G** Feel drug effect **H** Feel high **I** Feel paranoid **J** Feel stoned **K** Feel tired **L** Like drug effect **M** Mentally impaired **N** Want alcohol **O** Want food **P** Want more drug


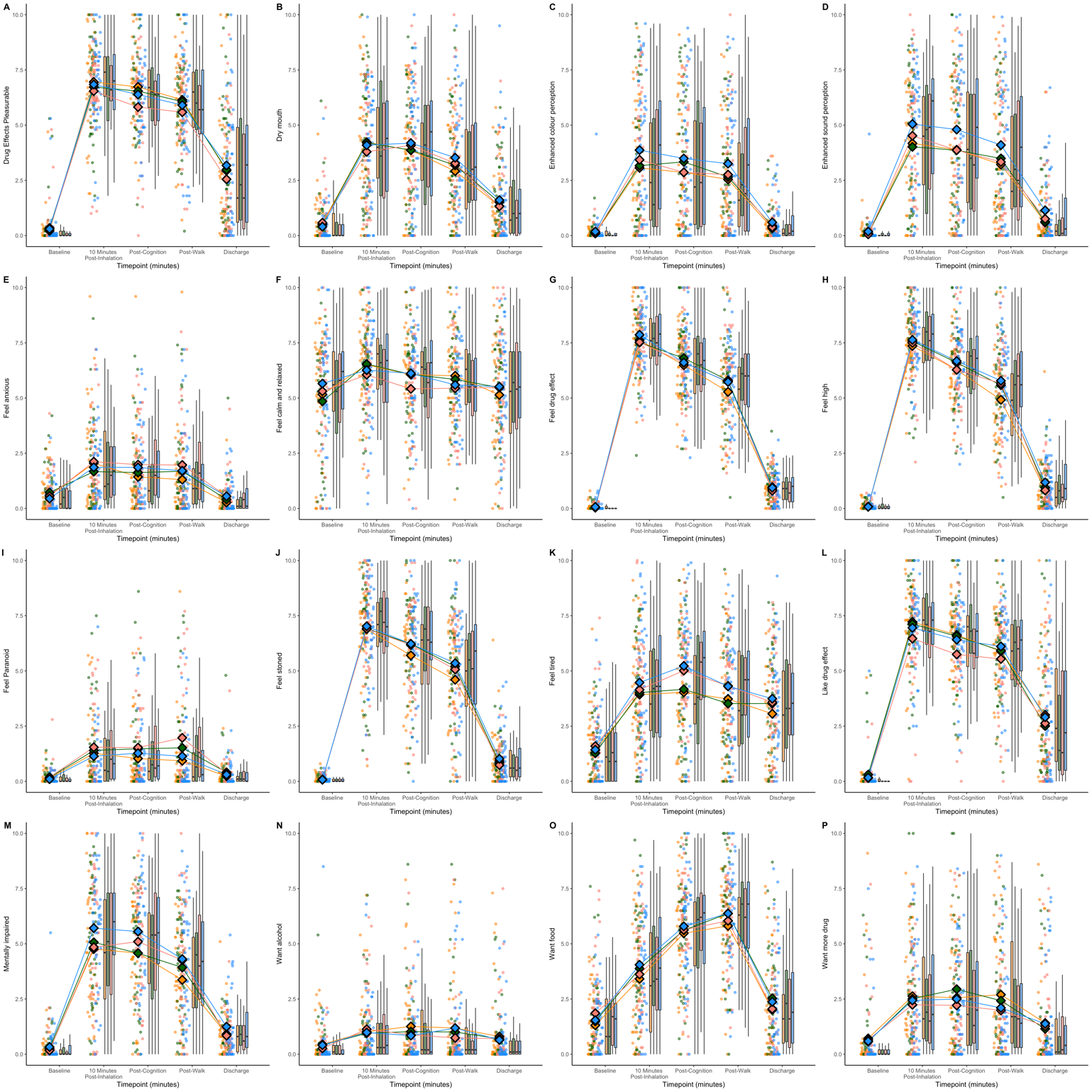


0:1

1:1

2:1

3:1

**eFigure 9** Area under the curve for subjective drug effects, as measured by visual analogues scales (VAS), at each time point, stratified by CBD:THC ratios. Circles show individual data points, diamonds show mean values, boxplots show median and interquartile range and half violin plots show the distribution of participants.

**A** Drug effects pleasurable **B** Dry mouth **C** Enhanced colour perception **D** Enhanced sound perception **E** Feel anxious **F** Feel calm and relaxed **G** Feel drug effect **H** Feel high **I** Feel paranoid **J** Feel stoned **K** Feel tired **L** Like drug effect **M** Mentally impaired **N** Want alcohol **O** Want food **P** Want more drug


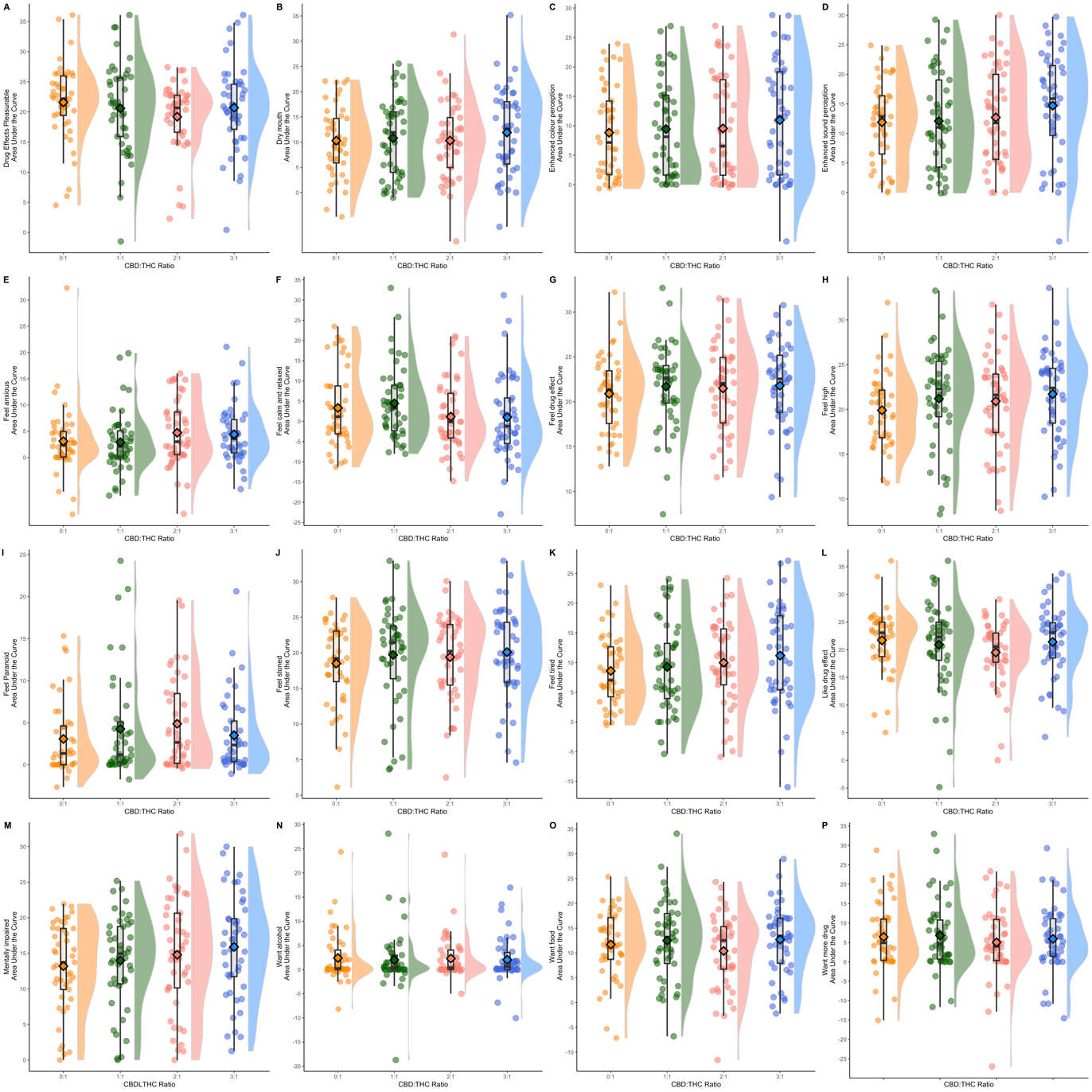


0:1

1:1

2:1

3:1

| **eTable 11** Subjective drug effects measured by visual analogue scales (VAS) by CBD:THC ratio | | | | | |  |
| --- | --- | --- | --- | --- | --- | --- |
|  |  | **CBD:THC Ratio** | | | |  |
|  |  | **0:1** | **1:1** | **2:1** | **3:1** |  |
| **Outcome** |  | **Drug effects pleasurable** | | | |  |
| **Baseline** | **Mean (SD)** | 0.21 (0.730 | 0.34 (1.06) | 0.29 (0.87) | 0.30 (1.01) |  |
| **10 minutes** | **Mean (SD)** | 6.95 (1.91) | 6.72 (1.99) | 6.54 (1.82) | 6.84 (1.94) |  |
| **Post-cognition** | **Mean (SD)** | 6.73 (1.65) | 6.54 (1.85) | 5.82 (1.98) | 6.39 (1.75) |  |
| **Post-walk** | **Mean (SD)** | 6.14 (2.01) | 6.07 (2.07) | 5.59 (1.79) | 5.91 (2.24) |  |
| **Discharge** | **Mean (SD)** | 2.94 (2.95) | 2.99 (2.73) | 2.55 (2.50) | 3.18 (2.73) |  |
| **Peak effects** | **EMM (SE)** | 6.74 (0.328) | 6.38 (0.328) | 6.26 (0.328) | 6.54 (0.328) |  |
| **Significant differences?** | | No | No | No | No |  |
| **AUC** | **EMM (SE)** | 21.6 (.01) | 20.6 (1.0) | 19.2 (1.0) | 20.7 (1.0) |  |
| **Significant differences?** | | No | No | No | No |  |
|  |  | **Dry mouth** | | | |  |
| **Baseline** | **Mean (SD)** | 0.56 (0.92) | 0.50 (1.08) | 0.58 (1.19) | 0.40 (0.90) |  |
| **10 minutes** | **Mean (SD)** | 4.22 (2.60) | 4.20 (2.86) | 3.79 (2.38) | 4.09 (2.51) |  |
| **Post-cognition** | **Mean (SD)** | 3.86 (2.28) | 3.86 (2.67) | 4.09 (2.58) | 4.19 (2.75) |  |
| **Post-walk** | **Mean (SD)** | 2.92 (2.11) | 3.18 (2.44) | 3.27 (2.48) | 3.52 (2.50) |  |
| **Discharge** | **Mean (SD)** | 1.34 (1.62) | 1.51 (1.59) | 1.32 (1.60) | 1.60 (2.06) |  |
| **Peak effects** | **EMM (SE)** | 3.66 (0.412) | 3.70 (0.412) | 3.21 (0.412) | 3.69 (0.412) |  |
| **Significant differences?** | | No | No | No | No |  |
| **AUC** | **EMM (SE)** | 10.3 (1.14) | 10.8 (1.14) | 10.3 (1.14) | 11.9 (1.14) |  |
| **Significant differences?** | | No | No | No | No |  |
|  | | **Enhanced colour perception** | | | |  |
| **Baseline** | **Mean (SD)** | 0.10 (0.17) | 0.13 (0.30) | 0.09 (0.22) | 0.18 (0.70) |  |
| **10 minutes** | **Mean (SD)** | 3.06 (2.63) | 3.16 (3.26) | 3.43 (2.96) | 3.87 (3.11) |  |
| **Post-cognition** | **Mean (SD)** | 2.86 (2.74) | 3.34 (2.93) | 2.86 (2.67) | 3.49 (3.02) |  |
| **Post-walk** | **Mean (SD)** | 2.54 (2.52) | 2.65 (2.36) | 2.76 (2.85) | 3.26 (2.88) |  |
| **Discharge** | **Mean (SD)** | 0.33 (0.66) | 0.43 (0.81) | 0.38 (0.69) | 0.60 (0.87) |  |
| **Peak effects** | **EMM (SE)** | 2.96 (0.451) | 3.03 (0.451) | 3.34 (0.451) | 3.69 (0.451) |  |
| **Significant differences?** | | No | No | No | No |  |
| **AUC** | **EMM (SE)** | 8.83 (1.27) | 9.44 (1.27) | 9.55 (1.27) | 10.97 (1.27) |  |
| **Significant differences?** | | No | No | No | No |  |
|  | | **Enhanced sound perception** | | | |  |
| **Baseline** | **Mean (SD)** | 0.10 (0.27) | 0.08 (0.17) | 0.06 (0.15) | 0.18 (0.69) |  |
| **10 minutes** | **Mean (SD)** | 4.18 (2.57) | 4.08 (2.98) | 4.52 (2.82) | 5.05 (2.53) |  |
| **Post-cognition** | **Mean (SD)** | 3.89 (2.78) | 3.86 (2.98) | 3.87 (2.89) | 4.80 (2.99) |  |
| **Post-walk** | **Mean (SD)** | 3.22 (2.84) | 3.49 (2.66) | 3.33 (2.91) | 4.10 (2.90) |  |
| **Discharge** | **Mean (SD)** | 0.59 (1.20) | 0.63 (1.04) | 0.75 (1.32) | 1.14 (1.63) |  |
| **Peak effects** | **EMM (SE)** | 4.08 (0.416) | 3.94 (0.416) | 4.46 (0.416) | 4.87 (0.416) |  |
| **Significant differences?** |  | No | No | No | No |  |
| **AUC** | **EMM (SE)** | 11.9 (1.21) | 12.1 (1.21) | 12.7 (1.21) | 14.7 (1.21) |  |
| **Significant differences?** |  | No | No | No | No |  |
|  | |  | | | |  |
|  | | **Feel anxious** | | | |  |
| **Baseline** | **Mean (SD)** | 0.60 (0.81) | 0.72 (0.97) | 0.56 (0.91) | 0.45 (0.68) |  |
| **10 minutes** | **Mean (SD)** | 2.05 (2.18) | 1.67 (1.87) | 2.12 (1.97) | 1.86 (1.71) |  |
| **Post-cognition** | **Mean (SD)** | 1.43 (1.80) | 1.63 (1.66) | 1.98 (1.71) | 1.86 (1.68) |  |
| **Post-walk** | **Mean (SD)** | 1.31 (1.68) | 1.63 (2.06) | 1.98 (1.95) | 1.86 (1.85) |  |
| **Discharge** | **Mean (SD)** | 0.27 (0.50) | 0.41 (0.80) | 0.55 (0.80) | 0.55 (0.77) |  |
| **Peak effects** | **EMM (SE)** | 1.45 (0.292) | 0.95 (0.292) | 1.56 (0.292) | 1.42 (0.292) |  |
| **Significant differences?** |  | No | No | No | No |  |
| **AUC** | **EMM (SE)** | 3.10 (0.885) | 2.90 (0.885) | 4.71 (0.885) | 4.41 (0.885) |  |
| **Significant differences?** |  | No | No | No | No |  |
|  | | **Feel calm and relaxed** | | | |  |
| **Baseline** | **Mean (SD)** | 5.16 (2.69) | 4.85 (2.43) | 5.31 (2.44) | 5.66 (2.40) |  |
| **10 minutes** | **Mean (SD)** | 6.51 (1.98) | 6.54 (1.73) | 6.06 (1.94) | 6.26 (2.35) |  |
| **Post-cognition** | **Mean (SD)** | 6.06 (1.91) | 6.07 (1.98) | 5.42 (2.20) | 6.11 (2.11) |  |
| **Post-walk** | **Mean (SD)** | 6.01 (2.11) | 5.84 (1.91) | 5.44 (1.95) | 5.60 (1.88) |  |
| **Discharge** | **Mean (SD)** | 5.14 (2.49) | 5.47 (2.08) | 5.48 (2.61) | 5.52 (2.17) |  |
| **Peak effects** | **EMM (SE)** | 1.34 (0.451) | 1.69 (0.451) | 0.75 (0.451) | 0.61 (0.451) |  |
| **Significant differences?** |  | No | No | No | No |  |
| **AUC** | **EMM (SE)** | 3.32 (1.41) | 4.45 (1.41) | 1.17 (1.41) | 1.00 (1.41) |  |
| **Significant differences?** |  | No | No | No | No |  |
|  | | **Feel drug effect** | | | |  |
| **Baseline** | **Mean (SD)** | 0.06 (0.15) | 0.05 (0.13) | 0.04 (0.09) | 0.07 (0.18) |  |
| **10 minutes** | **Mean (SD)** | 7.62 (1.33) | 7.51 (1.46) | 7.53 (1.45) | 7.87 (1.40) |  |
| **Post-cognition** | **Mean (SD)** | 6.49 (1.58) | 6.81 (1.63) | 6.60 (1.65) | 6.61 (1.77) |  |
| **Post-walk** | **Mean (SD)** | 5.28 (1.83) | 5.80 (1.91) | 5.75 (1.98) | 5.73 (1.79) |  |
| **Discharge** | **Mean (SD)** | 0.88 (0.64) | 0.96 (0.71) | 0.81 (0.64) | 0.94 (0.73) |  |
| **Peak effects** | **EMM (SE)** | 7.55 (0.216) | 7.46 (0.216) | 7.49 (0.216) | 7.80 (0.216) |  |
| **Significant differences?** |  | No | No | No | No |  |
| **AUC** | **EMM (SE)** | 20.9 (0.693) | 21.7 (0.693) | 21.5 (0.693) | 21.8 (0.693) |  |
| **Significant differences?** |  | No | No | No | No |  |
|  | | **Feel high** | | | |  |
| **Baseline** | **Mean (SD)** | 0.08 (0.16) | 0.08 (0.15) | 0.08 (0.17) | 0.07 (0.16) |  |
| **10 minutes** | **Mean (SD)** | 7.36 (1.28) | 7.58 (1.88) | 7.50 (1.69) | 7.64 (1.35) |  |
| **Post-cognition** | **Mean (SD)** | 6.28 (1.42) | 6.60 (1.71) | 6.28 (1.64) | 6.67 (1.72) |  |
| **Post-walk** | **Mean (SD)** | 4.92 (1.99) | 5.58 (1.98) | 5.66 (2.07) | 5.78 (1.86) |  |
| **Discharge** | **Mean (SD)** | 0.82 (1.06) | 0.94 (0.87) | 0.82 (0.81) | 1.18 (1.02) |  |
| **Peak effects** | **EMM (SE)** | 7.28 (0.243) | 7.49 (0.243) | 7.42 (0.243) | 7.56 (0.243) |  |
| **Significant differences?** |  | No | No | No | No |  |
| **AUC** | **EMM (SE)** | 19.9 (0.737) | 21.2 (0.737) | 20.9 (0.737) | 21.7 (0.737) |  |
| **Significant differences?** |  | No | No | No | No |  |
|  | | **Feel paranoid** | | | |  |
| **Baseline** | **Mean (SD)** | 0.14 (0.26) | 0.17 (0.31) | 0.17 (0.48) | 0.10 (0.17) |  |
| **10 minutes** | **Mean (SD)** | 1.28 (1.71) | 1.40 (1.98) | 1.54 (1.70) | 1.14 (1.34) |  |
| **Post-cognition** | **Mean (SD)** | 1.03 (1.55) | 1.48 (2.08) | 1.53 (1.75) | 1.27 (1.58) |  |
| **Post-walk** | **Mean (SD)** | 0.93 (1.60) | 1.52 (2.13) | 1.97 (2.28) | 1.13 (1.68) |  |
| **Discharge** | **Mean (SD)** | 0.23 (0.42) | 0.41 (0.88) | 0.36 (0.71) | 0.30 (0.50) |  |
| **Peak effects** | **EMM (SE)** | 1.14 (0.264) | 1.23 (0.264) | 1.35 (0.267) | 1.02 (0.261) |  |
| **Significant differences?** |  | No | No | No | No |  |
| **AUC** | **EMM (SE)** | 30.7 (0.822) | 4.25 (0.822) | 4.87 (0.830) | 3.49 (0.813) |  |
| **Significant differences?** |  | No | No | No | No |  |
|  | | **Feel stoned** | | | |  |
| **Baseline** | **Mean (SD)** | 0.09 (0.17) | 0.08 (0.14) | 0.08 (0.14) | 0.07 (0.15) |  |
| **10 minutes** | **Mean (SD)** | 6.89 (1.89) | 6.92 (2.27) | 6.91 (2.21) | 7.02 (1.84) |  |
| **Post-cognition** | **Mean (SD)** | 5.70 (2.17) | 6.22 (2.30) | 6.17 (2.07) | 6.23 (2.09) |  |
| **Post-walk** | **Mean (SD)** | 4.60 (2.16) | 5.20 (2.58) | 5.07 (2.21) | 5.35 (2.43) |  |
| **Discharge** | **Mean (SD)** | 0.92 (1.20) | 0.83 (0.85) | 0.74 (0.75) | 1.02 (1.16) |  |
| **Peak effects** | **EMM (SE)** | 6.80 (0.31) | 6.84 (0.31) | 6.83 (0.31) | 6.95 (0.31) |  |
| **Significant differences?** |  | No | No | No | No |  |
| **AUC** | **EMM (SE)** | 18.5 (0.936) | 19.7 (0.936) | 19.4 (0.936) | 20.1 (0.936) |  |
| **Significant differences?** |  | No | No | No | No |  |
|  | | **Feel tired** | | | |  |
| **Baseline** | **Mean (SD)** | 1.41 (1.53) | 1.28 (1.31) | 1.59 (1.70) | 1.43 (1.61) |  |
| **10 minutes** | **Mean (SD)** | 3.93 (2.39) | 4.02 (2.51) | 4.15 (2.30) | 4.47 (2.85) |  |
| **Post-cognition** | **Mean (SD)** | 4.03 (2.61) | 4.17 (2.80) | 5.02 (1.99) | 5.23 (2.70) |  |
| **Post-walk** | **Mean (SD)** | 3.73 (2.56) | 5.52 (2.58) | 4.32 (1.95) | 4.30 (2.47) |  |
| **Discharge** | **Mean (SD)** | 3.06 (2.25) | 3.52 (2.41) | 3.58 (2.38) | 3.74 (2.20) |  |
| **Peak effects** | **EMM (SE)** | 2.51 (0.372) | 2.74 (0.372) | 2.56 (0.372) | 3.04 (0.372) |  |
| **Significant differences?** |  | No | No | No | No |  |
| **AUC** | **EMM (SE)** | 8.60 (1.08) | 9.27 (1.08) | 10.00 (1.08) | 11.18 (1.08) |  |
| **Significant differences?** |  | No | No | No | No |  |
|  | | **Like drug effect** | | | |  |
| **Baseline** | **Mean (SD)** | 0.16 (0.70) | 0.30 (0.99) | 0.15 (0.49) | 0.17 (0.75) |  |
| **10 minutes** | **Mean (SD)** | 7.22 (1.85) | 7.13 (1.86) | 6.47 (2.06) | 6.95 (1.81) |  |
| **Post-cognition** | **Mean (SD)** | 6.63 (1.65) | 6.57 (1.98) | 5.75 (1.81) | 6.42 (1.92) |  |
| **Post-walk** | **Mean (SD)** | 5.89 (1.88) | 5.92 (2.08) | 5.54 (2.04) | 6.11 (1.89) |  |
| **Discharge** | **Mean (SD)** | 3.00 (2.62) | 2.51 (2.72) | 2.62 (2.68) | 2.91 (2.76) |  |
| **Peak effects** | **EMM (SE)** | 7.06 (0.315) | 6.83 (0.315) | 6.32 (0.315) | 6.79 (0.315) |  |
| **Significant differences?** |  | No | No | No | No |  |
| **AUC** | **EMM (SE)** | 21.7 (0.972) | 20.9 (0.972) | 19.5 (0.972) | 21.4 (0.972) |  |
| **Significant differences?** |  | No | No | No | No |  |
|  | | **Mentally impaired** | | | |  |
| **Baseline** | **Mean (SD)** | 0.18 (0.40) | 0.26 (0.50) | 0.17 (0.44) | 0.33 (0.88) |  |
| **10 minutes** | **Mean (SD)** | 4.76 (2.89) | 5.07 (2.55) | 4.85 (2.94) | 5.71 (2.50) |  |
| **Post-cognition** | **Mean (SD)** | 4.58 (2.30) | 4.58 (2.44) | 5.10 (2.54) | 5.56 (2.27) |  |
| **Post-walk** | **Mean (SD)** | 3.37 (2.05) | 3.93 (2.25) | 3.26 (2.56) | 4.31 (2.47) |  |
| **Discharge** | **Mean (SD)** | 0.91 (1.00) | 0.89 (0.69) | 0.84 (0.83) | 1.25 (1.36) |  |
| **Peak effects** | **EMM (SE)** | 4.58 (0.394) | 4.81 (0.394) | 4.67 (0.394) | 5.38 (0.394) |  |
| **Significant differences?** |  | No | No | No | No |  |
| **AUC** | **EMM (SE)** | 13.2 (1.05) | 14.0 (1.05) | 14.8 (1.05) | 15.9 (1.05) |  |
| **Significant differences?** |  | No | No | No | No |  |
|  | | **Want alcohol** | | | |  |
| **Baseline** | **Mean (SD)** | 0.45 (0.80) | 0.41 (0.93) | 0.26 (0.48) | 0.41 (1.32) |  |
| **10 minutes** | **Mean (SD)** | 1.02 (1.64) | 0.95 (1.52) | 1.13 (1.67) | 0.98 (1.52) |  |
| **Post-cognition** | **Mean (SD)** | 1.26 (1.85) | 1.02 (1.76) | 0.88 (1.56) | 0.84 (1.26) |  |
| **Post-walk** | **Mean (SD)** | 1.19 (2.00) | 1.01 (1.95) | 0.74 (1.46) | 1.18 (1.99) |  |
| **Discharge** | **Mean (SD)** | 0.80 (1.70) | 0.70 (1.29) | 0.70 (1.50) | 0.65 (1.23) |  |
| **Peak effects** | **EMM (SE)** | 0.57 (0.234) | 0.54 (0.234) | 0.87 (0.234) | 0.57 (0.234) |  |
| **Significant differences?** |  | No | No | No | No |  |
| **AUC** | **EMM (SE)** | 2.39 (0.775) | 1.99 (0.775) | 2.28 (0.775) | 2.03 (0.775) |  |
| **Significant differences?** |  | No | No | No | No |  |
|  | | **Want food** | | | |  |
| **Baseline** | **Mean (SD)** | 1.31 (1.43) | 1.49 (1.81) | 1.86 (1.93) | 1.54 (1.36) |  |
| **10 minutes** | **Mean (SD)** | 3.41 (2.53) | 3.88 (2.49) | 3.62 (2.13) | 4.05 (2.31) |  |
| **Post-cognition** | **Mean (SD)** | 5.48 (2.33) | 5.62 (2.40) | 5.63 (2.47) | 5.79 (2.25) |  |
| **Post-walk** | **Mean (SD)** | 5.81 (2.05) | 6.37 (2.46) | 6.04 (1.89) | 6.38 (2.27) |  |
| **Discharge** | **Mean (SD)** | 2.01 (2.05) | 2.53 (2.28) | 2.05 (1.91) | 2.37 (2.31) |  |
| **Peak effects** | **EMM (SE)** | 2.10 (0.367) | 2.39 (0.367) | 1.76 (0.367) | 2.51 (0.367) |  |
| **Significant differences?** |  | No | No | No | No |  |
| **AUC** | **EMM (SE)** | 2.39 (0.775) | 1.99 (0.775) | 2.28 (0.775) | 2.03 (0.775) |  |
| **Significant differences?** |  | No | No | No | No |  |
|  | | **Want more drug** | | | |  |
| **Baseline** | **Mean (SD)** | 0.72 (1.93) | 0.57 (1.42) | 0.66 (1.61) | 0.59 (1.61) |  |
| **10 minutes** | **Mean (SD)** | 2.63 (2.45) | 2.51 (2.57) | 2.24 (2.06) | 2.44 (2.45) |  |
| **Post-cognition** | **Mean (SD)** | 2.56 (2.47) | 2.94 (2.76) | 2.21 (2.33) | 2.50 (2.37) |  |
| **Post-walk** | **Mean (SD)** | 2.70 (2.65) | 2.42 (2.46) | 1.99 (2.08) | 2.10 (2.34) |  |
| **Discharge** | **Mean (SD)** | 1.41 (2.31) | 1.15 (2.13) | 1.27 (2.02) | 1.38 (2.22) |  |
| **Peak effects** | **EMM (SE)** | 1.91 (0.431) | 1.94 (0.431) | 1.58 (0.431) | 1.85 (0.431) |  |
| **Significant differences?** |  | No | No | No | No |  |
| **AUC** | **EMM (SE)** | 6.43 (1.32) | 6.77 (1.32) | 5.02 (1.32) | 5.92 (1.32) |  |
| **Significant differences?** |  | No | No | No | No |  |

| **eTable 12** Results of linear mixed models of visual analogue scales (VAS) for each contrast between CBD:THC ratios. Statistically significant rows are presented in bold. AUC, area under the curve; CI, confidence interval; EMM, estimated marginal mean. | | | | |
| --- | --- | --- | --- | --- |
|  |  | | | |
| **Contrast** | **EMM difference** | **Lower 95% CI** | **Upper 95% CI** | **p-value** |
|  | **Drug effects pleasurable AUC** |  |  |  |
| 0:1 - 1:1 | 0.972 | -1.088 | 3.033 | 0.778 |
| 0:1 - 2:1 | 2.373 | 0.312 | 4.433 | 0.099 |
| 0:1 - 3:1 | 0.895 | -1.166 | 2.955 | 0.818 |
| 1:1 - 2:1 | 1.401 | -0.660 | 3.461 | 0.521 |
| 1:1 - 3:1 | -0.077 | -2.138 | 1.983 | 1.000 |
| 2:1 - 3:1 | -1.478 | -3.538 | 0.582 | 0.474 |
|  | **Drug effects pleasurable AUC (visit included in model)** | | | |
| 0:1 - 1:1 | 0.973 | -1.096 | 3.041 | 0.780 |
| 0:1 - 2:1 | 2.373 | 0.305 | 4.442 | 0.101 |
| 0:1 - 3:1 | 0.892 | -1.188 | 2.972 | 0.823 |
| 1:1 - 2:1 | 1.401 | -0.668 | 3.469 | 0.524 |
| 1:1 - 3:1 | -0.080 | -2.164 | 2.003 | 1.000 |
| 2:1 - 3:1 | -1.481 | -3.566 | 0.604 | 0.483 |
|  | **Drug effects pleasurable peak** | | | |
| 0:1 - 1:1 | 0.358 | -0.290 | 1.006 | 0.683 |
| 0:1 - 2:1 | 0.484 | -0.164 | 1.133 | 0.437 |
| 0:1 - 3:1 | 0.196 | -0.453 | 0.844 | 0.929 |
| 1:1 - 2:1 | 0.127 | -0.522 | 0.775 | 0.979 |
| 1:1 - 3:1 | -0.162 | -0.810 | 0.486 | 0.958 |
| 2:1 - 3:1 | -0.289 | -0.937 | 0.359 | 0.806 |
|  | **Drug effects pleasurable peak (visit included in model)** | | | |
| 0:1 - 1:1 | 0.357 | -0.294 | 1.007 | 0.688 |
| 0:1 - 2:1 | 0.483 | -0.168 | 1.133 | 0.444 |
| 0:1 - 3:1 | 0.204 | -0.450 | 0.858 | 0.923 |
| 1:1 - 2:1 | 0.126 | -0.524 | 0.777 | 0.980 |
| 1:1 - 3:1 | -0.153 | -0.808 | 0.502 | 0.966 |
| 2:1 - 3:1 | -0.279 | -0.935 | 0.377 | 0.827 |
|  | **Dry mouth AUC** |  |  |  |
| 0:1 - 1:1 | -0.564 | -3.085 | 1.956 | 0.969 |
| 0:1 - 2:1 | -0.039 | -2.560 | 2.482 | 1.000 |
| 0:1 - 3:1 | -1.678 | -4.199 | 0.842 | 0.539 |
| 1:1 - 2:1 | 0.525 | -1.996 | 3.046 | 0.975 |
| 1:1 - 3:1 | -1.114 | -3.635 | 1.407 | 0.810 |
| 2:1 - 3:1 | -1.639 | -4.160 | 0.882 | 0.558 |
|  | **Dry mouth AUC (visit included in model)** | | | |
| 0:1 - 1:1 | -0.538 | -3.047 | 1.971 | 0.973 |
| 0:1 - 2:1 | 0.000 | -2.509 | 2.510 | 1.000 |
| 0:1 - 3:1 | -1.876 | -4.399 | 0.647 | 0.442 |
| 1:1 - 2:1 | 0.538 | -1.971 | 3.048 | 0.973 |
| 1:1 - 3:1 | -1.338 | -3.865 | 1.189 | 0.711 |
| 2:1 - 3:1 | -1.876 | -4.406 | 0.653 | 0.444 |
|  | **Dry mouth peak** | | | |
| 0:1 - 1:1 | -0.040 | -0.963 | 0.883 | 1.000 |
| 0:1 - 2:1 | 0.451 | -0.472 | 1.374 | 0.758 |
| 0:1 - 3:1 | -0.029 | -0.952 | 0.894 | 1.000 |
| 1:1 - 2:1 | 0.491 | -0.432 | 1.414 | 0.707 |
| 1:1 - 3:1 | 0.011 | -0.912 | 0.934 | 1.000 |
| 2:1 - 3:1 | -0.480 | -1.403 | 0.443 | 0.722 |

|  | **Dry mouth peak (visit included in model)** | | | |
| --- | --- | --- | --- | --- |
| 0:1 - 1:1 | -0.036 | -0.961 | 0.889 | 1.000 |
| 0:1 - 2:1 | 0.457 | -0.468 | 1.382 | 0.752 |
| 0:1 - 3:1 | -0.059 | -0.990 | 0.871 | 0.999 |
| 1:1 - 2:1 | 0.493 | -0.432 | 1.418 | 0.706 |
| 1:1 - 3:1 | -0.023 | -0.955 | 0.908 | 1.000 |
| 2:1 - 3:1 | -0.517 | -1.449 | 0.416 | 0.680 |
|  | **Enhanced colour perception AUC** | | | |
| 0:1 - 1:1 | -0.617 | -2.695 | 1.461 | 0.933 |
| 0:1 - 2:1 | -0.720 | -2.799 | 1.358 | 0.898 |
| 0:1 - 3:1 | -2.148 | -4.227 | -0.070 | 0.164 |
| 1:1 - 2:1 | -0.104 | -2.182 | 1.975 | 1.000 |
| 1:1 - 3:1 | -1.531 | -3.610 | 0.547 | 0.450 |
| 2:1 - 3:1 | -1.428 | -3.506 | 0.651 | 0.512 |
|  | **Enhanced colour perception AUC (visit included in model)** | | | |
| 0:1 - 1:1 | -0.626 | -2.709 | 1.457 | 0.930 |
| 0:1 - 2:1 | -0.734 | -2.818 | 1.349 | 0.893 |
| 0:1 - 3:1 | -2.080 | -4.174 | 0.015 | 0.194 |
| 1:1 - 2:1 | -0.108 | -2.191 | 1.975 | 1.000 |
| 1:1 - 3:1 | -1.453 | -3.552 | 0.645 | 0.505 |
| 2:1 - 3:1 | -1.345 | -3.445 | 0.755 | 0.571 |
|  | **Enhanced colour perception peak** | | | |
| 0:1 - 1:1 | -0.071 | -0.977 | 0.835 | 0.999 |
| 0:1 - 2:1 | -0.376 | -1.282 | 0.530 | 0.838 |
| 0:1 - 3:1 | -0.729 | -1.635 | 0.177 | 0.371 |
| 1:1 - 2:1 | -0.304 | -1.210 | 0.602 | 0.906 |
| 1:1 - 3:1 | -0.658 | -1.564 | 0.248 | 0.463 |
| 2:1 - 3:1 | -0.353 | -1.259 | 0.553 | 0.861 |
|  | **Enhanced colour perception peak (visit included in model)** | | | |
| 0:1 - 1:1 | -0.079 | -0.983 | 0.825 | 0.998 |
| 0:1 - 2:1 | -0.387 | -1.292 | 0.517 | 0.824 |
| 0:1 - 3:1 | -0.670 | -1.579 | 0.239 | 0.450 |
| 1:1 - 2:1 | -0.308 | -1.213 | 0.596 | 0.902 |
| 1:1 - 3:1 | -0.591 | -1.502 | 0.320 | 0.560 |
| 2:1 - 3:1 | -0.283 | -1.194 | 0.629 | 0.924 |
|  | **Enhanced sound perception AUC** | | | |
| 0:1 - 1:1 | -0.211 | -2.316 | 1.893 | 0.997 |
| 0:1 - 2:1 | -0.722 | -2.826 | 1.382 | 0.900 |
| 0:1 - 3:1 | -2.775 | -4.879 | -0.670 | 0.044 |
| 1:1 - 2:1 | -0.511 | -2.615 | 1.594 | 0.962 |
| 1:1 - 3:1 | -2.563 | -4.667 | -0.459 | 0.072 |
| 2:1 - 3:1 | -2.053 | -4.157 | 0.052 | 0.207 |
|  | **Enhanced sound perception AUC (visit included in model)** | | | |
| 0:1 - 1:1 | -0.206 | -2.317 | 1.906 | 0.997 |
| 0:1 - 2:1 | -0.714 | -2.826 | 1.398 | 0.904 |
| 0:1 - 3:1 | -2.815 | -4.938 | -0.692 | 0.042 |
| 1:1 - 2:1 | -0.508 | -2.619 | 1.603 | 0.962 |
| 1:1 - 3:1 | -2.609 | -4.735 | -0.482 | 0.069 |
| 2:1 - 3:1 | -2.101 | -4.229 | 0.028 | 0.198 |
|  | **Enhanced sound perception peak** | | | |
| 0:1 - 1:1 | 0.133 | -0.750 | 1.017 | 0.990 |
| 0:1 - 2:1 | -0.387 | -1.270 | 0.497 | 0.815 |
| 0:1 - 3:1 | -0.793 | -1.677 | 0.090 | 0.274 |
| 1:1 - 2:1 | -0.520 | -1.404 | 0.364 | 0.637 |
| 1:1 - 3:1 | -0.927 | -1.810 | -0.043 | 0.155 |
| 2:1 - 3:1 | -0.407 | -1.290 | 0.477 | 0.790 |

|  | **Enhanced sound perception peak (visit included in model)** | | | |
| --- | --- | --- | --- | --- |
| 0:1 - 1:1 | 0.134 | -0.753 | 1.021 | 0.990 |
| 0:1 - 2:1 | -0.386 | -1.273 | 0.502 | 0.818 |
| 0:1 - 3:1 | -0.799 | -1.691 | 0.093 | 0.276 |
| 1:1 - 2:1 | -0.520 | -1.407 | 0.367 | 0.641 |
| 1:1 - 3:1 | -0.933 | -1.826 | -0.040 | 0.157 |
| 2:1 - 3:1 | -0.413 | -1.307 | 0.481 | 0.788 |
|  | **Feel anxious AUC** | | | |
| 0:1 - 1:1 | 0.205 | -1.799 | 2.210 | 0.997 |
| 0:1 - 2:1 | -1.607 | -3.612 | 0.397 | 0.374 |
| 0:1 - 3:1 | -1.310 | -3.314 | 0.695 | 0.555 |
| 1:1 - 2:1 | -1.813 | -3.818 | 0.192 | 0.268 |
| 1:1 - 3:1 | -1.515 | -3.520 | 0.490 | 0.427 |
| 2:1 - 3:1 | 0.298 | -1.707 | 2.302 | 0.991 |
|  | **Feel anxious AUC (visit included in model)** | | | |
| 0:1 - 1:1 | 0.236 | -1.741 | 2.213 | 0.995 |
| 0:1 - 2:1 | -1.562 | -3.538 | 0.415 | 0.387 |
| 0:1 - 3:1 | -1.539 | -3.527 | 0.449 | 0.405 |
| 1:1 - 2:1 | -1.798 | -3.774 | 0.179 | 0.263 |
| 1:1 - 3:1 | -1.775 | -3.766 | 0.216 | 0.280 |
| 2:1 - 3:1 | 0.023 | -1.970 | 2.015 | 1.000 |
|  | **Feel anxious peak** | | | |
| 0:1 - 1:1 | 0.500 | -0.214 | 1.214 | 0.495 |
| 0:1 - 2:1 | -0.111 | -0.825 | 0.602 | 0.989 |
| 0:1 - 3:1 | 0.031 | -0.682 | 0.745 | 1.000 |
| 1:1 - 2:1 | -0.611 | -1.325 | 0.102 | 0.315 |
| 1:1 - 3:1 | -0.469 | -1.182 | 0.245 | 0.550 |
| 2:1 - 3:1 | 0.142 | -0.571 | 0.856 | 0.978 |
|  | **Feel anxious peak (visit included in model)** | | | |
| 0:1 - 1:1 | 0.515 | -0.177 | 1.207 | 0.442 |
| 0:1 - 2:1 | -0.089 | -0.781 | 0.604 | 0.994 |
| 0:1 - 3:1 | -0.080 | -0.776 | 0.616 | 0.996 |
| 1:1 - 2:1 | -0.604 | -1.296 | 0.089 | 0.299 |
| 1:1 - 3:1 | -0.595 | -1.292 | 0.102 | 0.318 |
| 2:1 - 3:1 | 0.009 | -0.689 | 0.706 | 1.000 |
|  | **Feel calm and relaxed AUC** | | | |
| 0:1 - 1:1 | -1.128 | -4.175 | 1.918 | 0.878 |
| 0:1 - 2:1 | 2.147 | -0.899 | 5.193 | 0.489 |
| 0:1 - 3:1 | 2.321 | -0.726 | 5.367 | 0.420 |
| 1:1 - 2:1 | 3.275 | 0.229 | 6.322 | 0.138 |
| 1:1 - 3:1 | 3.449 | 0.403 | 6.495 | 0.108 |
| 2:1 - 3:1 | 0.173 | -2.873 | 3.220 | 0.999 |
|  | **Feel calm and relaxed AUC (visit included in model)** | | | |
| 0:1 - 1:1 | -1.128 | -4.186 | 1.930 | 0.880 |
| 0:1 - 2:1 | 2.148 | -0.911 | 5.206 | 0.493 |
| 0:1 - 3:1 | 2.317 | -0.758 | 5.392 | 0.430 |
| 1:1 - 2:1 | 3.276 | 0.218 | 6.334 | 0.141 |
| 1:1 - 3:1 | 3.445 | 0.365 | 6.525 | 0.115 |
| 2:1 - 3:1 | 0.169 | -2.913 | 3.252 | 1.000 |
|  | **Feel calm and relaxed peak** | | | |
| 0:1 - 1:1 | -0.349 | -1.403 | 0.706 | 0.910 |
| 0:1 - 2:1 | 0.596 | -0.459 | 1.650 | 0.667 |
| 0:1 - 3:1 | 0.733 | -0.321 | 1.788 | 0.501 |
| 1:1 - 2:1 | 0.944 | -0.110 | 1.999 | 0.276 |
| 1:1 - 3:1 | 1.082 | 0.028 | 2.137 | 0.169 |
| 2:1 - 3:1 | 0.138 | -0.917 | 1.192 | 0.994 |

|  | **Feel calm and relaxed peak (visit included in model)** | | | |
| --- | --- | --- | --- | --- |
| 0:1 - 1:1 | -0.348 | -1.407 | 0.710 | 0.911 |
| 0:1 - 2:1 | 0.596 | -0.462 | 1.655 | 0.669 |
| 0:1 - 3:1 | 0.730 | -0.334 | 1.794 | 0.513 |
| 1:1 - 2:1 | 0.945 | -0.114 | 2.003 | 0.279 |
| 1:1 - 3:1 | 1.078 | 0.012 | 2.145 | 0.180 |
| 2:1 - 3:1 | 0.134 | -0.933 | 1.201 | 0.994 |
|  | **Feel drug effect AUC** | | | |
| 0:1 - 1:1 | -0.839 | -2.143 | 0.465 | 0.568 |
| 0:1 - 2:1 | -0.605 | -1.909 | 0.699 | 0.787 |
| 0:1 - 3:1 | -0.910 | -2.215 | 0.394 | 0.498 |
| 1:1 - 2:1 | 0.234 | -1.070 | 1.538 | 0.984 |
| 1:1 - 3:1 | -0.072 | -1.376 | 1.233 | 1.000 |
| 2:1 - 3:1 | -0.306 | -1.610 | 0.999 | 0.965 |
|  | **Feel drug effect AUC (visit included in model)** | | | |
| 0:1 - 1:1 | -0.838 | -2.148 | 0.471 | 0.571 |
| 0:1 - 2:1 | -0.604 | -1.913 | 0.706 | 0.789 |
| 0:1 - 3:1 | -0.916 | -2.232 | 0.401 | 0.501 |
| 1:1 - 2:1 | 0.234 | -1.075 | 1.544 | 0.984 |
| 1:1 - 3:1 | -0.078 | -1.396 | 1.241 | 0.999 |
| 2:1 - 3:1 | -0.312 | -1.632 | 1.008 | 0.964 |
|  | **Feel drug effect peak** | | | |
| 0:1 - 1:1 | 0.093 | -0.373 | 0.560 | 0.978 |
| 0:1 - 2:1 | 0.060 | -0.406 | 0.526 | 0.994 |
| 0:1 - 3:1 | -0.244 | -0.711 | 0.222 | 0.717 |
| 1:1 - 2:1 | -0.033 | -0.500 | 0.433 | 0.999 |
| 1:1 - 3:1 | -0.338 | -0.804 | 0.129 | 0.465 |
| 2:1 - 3:1 | -0.304 | -0.771 | 0.162 | 0.555 |
|  | **Feel drug effect peak (visit included in model)** | | | |
| 0:1 - 1:1 | 0.095 | -0.373 | 0.563 | 0.977 |
| 0:1 - 2:1 | 0.063 | -0.405 | 0.530 | 0.993 |
| 0:1 - 3:1 | -0.257 | -0.727 | 0.213 | 0.690 |
| 1:1 - 2:1 | -0.032 | -0.500 | 0.435 | 0.999 |
| 1:1 - 3:1 | -0.352 | -0.823 | 0.119 | 0.437 |
| 2:1 - 3:1 | -0.320 | -0.791 | 0.152 | 0.523 |
|  | **Feel hungry AUC** | | | |
| 0:1 - 1:1 | -1.340 | -2.662 | -0.017 | 0.179 |
| 0:1 - 2:1 | -0.988 | -2.310 | 0.335 | 0.438 |
| 0:1 - 3:1 | -1.830 | -3.152 | -0.507 | 0.031 |
| 1:1 - 2:1 | 0.352 | -0.970 | 1.675 | 0.950 |
| 1:1 - 3:1 | -0.490 | -1.812 | 0.833 | 0.878 |
| 2:1 - 3:1 | -0.842 | -2.165 | 0.480 | 0.576 |
|  | **Feel hungry AUC (visit included in model)** | | | |
| 0:1 - 1:1 | -1.324 | -2.637 | -0.011 | 0.182 |
| 0:1 - 2:1 | -0.964 | -2.277 | 0.349 | 0.454 |
| 0:1 - 3:1 | -1.950 | -3.270 | -0.630 | 0.018 |
| 1:1 - 2:1 | 0.360 | -0.952 | 1.673 | 0.946 |
| 1:1 - 3:1 | -0.626 | -1.948 | 0.696 | 0.776 |
| 2:1 - 3:1 | -0.986 | -2.309 | 0.337 | 0.440 |
|  | **Feel hungry peak** | | | |
| 0:1 - 1:1 | -0.213 | -0.684 | 0.258 | 0.798 |
| 0:1 - 2:1 | -0.136 | -0.607 | 0.336 | 0.938 |
| 0:1 - 3:1 | -0.282 | -0.753 | 0.189 | 0.624 |
| 1:1 - 2:1 | 0.078 | -0.393 | 0.549 | 0.987 |
| 1:1 - 3:1 | -0.069 | -0.540 | 0.402 | 0.991 |
| 2:1 - 3:1 | -0.147 | -0.618 | 0.324 | 0.923 |

|  | **Feel hungry peak (visit included in model)** | | | |
| --- | --- | --- | --- | --- |
| 0:1 - 1:1 | -0.208 | -0.678 | 0.261 | 0.807 |
| 0:1 - 2:1 | -0.128 | -0.597 | 0.341 | 0.946 |
| 0:1 - 3:1 | -0.319 | -0.791 | 0.153 | 0.526 |
| 1:1 - 2:1 | 0.080 | -0.389 | 0.549 | 0.986 |
| 1:1 - 3:1 | -0.110 | -0.583 | 0.362 | 0.965 |
| 2:1 - 3:1 | -0.191 | -0.663 | 0.282 | 0.849 |
|  | **Feel paranoid AUC** | | | |
| 0:1 - 1:1 | -1.177 | -2.988 | 0.634 | 0.559 |
| 0:1 - 2:1 | -1.798 | -3.624 | 0.028 | 0.200 |
| 0:1 - 3:1 | -0.416 | -2.230 | 1.397 | 0.967 |
| 1:1 - 2:1 | -0.621 | -2.447 | 1.205 | 0.903 |
| 1:1 - 3:1 | 0.761 | -1.053 | 2.574 | 0.833 |
| 2:1 - 3:1 | 1.381 | -0.440 | 3.203 | 0.424 |
|  | **Feel paranoid AUC (visit included in model)** | | | |
| 0:1 - 1:1 | -1.122 | -2.890 | 0.646 | 0.579 |
| 0:1 - 2:1 | -1.780 | -3.563 | 0.003 | 0.190 |
| 0:1 - 3:1 | -0.576 | -2.351 | 1.198 | 0.914 |
| 1:1 - 2:1 | -0.658 | -2.441 | 1.125 | 0.879 |
| 1:1 - 3:1 | 0.545 | -1.233 | 2.324 | 0.926 |
| 2:1 - 3:1 | 1.204 | -0.580 | 2.987 | 0.527 |
|  | **Feel paranoid peak** | | | |
| 0:1 - 1:1 | -0.093 | -0.689 | 0.504 | 0.989 |
| 0:1 - 2:1 | -0.210 | -0.812 | 0.392 | 0.896 |
| 0:1 - 3:1 | 0.117 | -0.480 | 0.714 | 0.979 |
| 1:1 - 2:1 | -0.118 | -0.719 | 0.484 | 0.979 |
| 1:1 - 3:1 | 0.210 | -0.387 | 0.807 | 0.894 |
| 2:1 - 3:1 | 0.327 | -0.273 | 0.927 | 0.691 |
|  | **Feel paranoid peak (visit included in model)** | | | |
| 0:1 - 1:1 | -0.075 | -0.659 | 0.509 | 0.994 |
| 0:1 - 2:1 | -0.204 | -0.793 | 0.384 | 0.897 |
| 0:1 - 3:1 | 0.067 | -0.519 | 0.652 | 0.996 |
| 1:1 - 2:1 | -0.129 | -0.718 | 0.460 | 0.971 |
| 1:1 - 3:1 | 0.142 | -0.445 | 0.729 | 0.962 |
| 2:1 - 3:1 | 0.271 | -0.318 | 0.860 | 0.791 |
|  | **Feel stoned AUC** | | | |
| 0:1 - 1:1 | -1.205 | -2.870 | 0.459 | 0.465 |
| 0:1 - 2:1 | -0.946 | -2.610 | 0.719 | 0.663 |
| 0:1 - 3:1 | -1.582 | -3.246 | 0.082 | 0.227 |
| 1:1 - 2:1 | 0.259 | -1.405 | 1.924 | 0.989 |
| 1:1 - 3:1 | -0.377 | -2.041 | 1.287 | 0.968 |
| 2:1 - 3:1 | -0.636 | -2.301 | 1.028 | 0.868 |
|  | **Feel stoned AUC (visit included in model)** | | | |
| 0:1 - 1:1 | -1.195 | -2.860 | 0.471 | 0.474 |
| 0:1 - 2:1 | -0.930 | -2.596 | 0.736 | 0.675 |
| 0:1 - 3:1 | -1.661 | -3.336 | 0.014 | 0.194 |
| 1:1 - 2:1 | 0.265 | -1.401 | 1.930 | 0.989 |
| 1:1 - 3:1 | -0.466 | -2.144 | 1.211 | 0.944 |
| 2:1 - 3:1 | -0.731 | -2.410 | 0.948 | 0.817 |
|  | **Feel stoned peak** | | | |
| 0:1 - 1:1 | -0.040 | -0.697 | 0.617 | 0.999 |
| 0:1 - 2:1 | -0.027 | -0.683 | 0.630 | 1.000 |
| 0:1 - 3:1 | -0.144 | -0.801 | 0.512 | 0.971 |
| 1:1 - 2:1 | 0.013 | -0.643 | 0.670 | 1.000 |
| 1:1 - 3:1 | -0.104 | -0.761 | 0.552 | 0.989 |
| 2:1 - 3:1 | -0.118 | -0.774 | 0.539 | 0.984 |

|  | **Feel stoned peak (visit included in model)** | | | |
| --- | --- | --- | --- | --- |
| 0:1 - 1:1 | -0.036 | -0.693 | 0.621 | 1.000 |
| 0:1 - 2:1 | -0.021 | -0.678 | 0.637 | 1.000 |
| 0:1 - 3:1 | -0.175 | -0.836 | 0.486 | 0.951 |
| 1:1 - 2:1 | 0.015 | -0.642 | 0.673 | 1.000 |
| 1:1 - 3:1 | -0.139 | -0.801 | 0.523 | 0.974 |
| 2:1 - 3:1 | -0.155 | -0.817 | 0.508 | 0.965 |
|  | **Feel tired AUC** | | | |
| 0:1 - 1:1 | -0.672 | -3.156 | 1.811 | 0.948 |
| 0:1 - 2:1 | -1.402 | -3.886 | 1.081 | 0.667 |
| 0:1 - 3:1 | -2.586 | -5.069 | -0.102 | 0.159 |
| 1:1 - 2:1 | -0.730 | -3.214 | 1.753 | 0.934 |
| 1:1 - 3:1 | -1.913 | -4.397 | 0.570 | 0.410 |
| 2:1 - 3:1 | -1.183 | -3.667 | 1.300 | 0.773 |
|  | **Feel tired AUC (visit included in model)** | | | |
| 0:1 - 1:1 | -0.640 | -3.101 | 1.821 | 0.953 |
| 0:1 - 2:1 | -1.354 | -3.815 | 1.107 | 0.685 |
| 0:1 - 3:1 | -2.829 | -5.303 | -0.354 | 0.103 |
| 1:1 - 2:1 | -0.714 | -3.174 | 1.747 | 0.937 |
| 1:1 - 3:1 | -2.189 | -4.667 | 0.290 | 0.288 |
| 2:1 - 3:1 | -1.475 | -3.955 | 1.006 | 0.630 |
|  | **Feel tired peak** | | | |
| 0:1 - 1:1 | -0.229 | -1.123 | 0.665 | 0.955 |
| 0:1 - 2:1 | -0.042 | -0.936 | 0.852 | 1.000 |
| 0:1 - 3:1 | -0.527 | -1.421 | 0.367 | 0.636 |
| 1:1 - 2:1 | 0.187 | -0.707 | 1.081 | 0.975 |
| 1:1 - 3:1 | -0.298 | -1.192 | 0.596 | 0.908 |
| 2:1 - 3:1 | -0.484 | -1.378 | 0.410 | 0.695 |
|  | **Feel tired peak (visit included in model)** | | | |
| 0:1 - 1:1 | -0.221 | -1.113 | 0.671 | 0.959 |
| 0:1 - 2:1 | -0.031 | -0.923 | 0.862 | 1.000 |
| 0:1 - 3:1 | -0.585 | -1.482 | 0.312 | 0.556 |
| 1:1 - 2:1 | 0.191 | -0.702 | 1.083 | 0.973 |
| 1:1 - 3:1 | -0.364 | -1.263 | 0.535 | 0.847 |
| 2:1 - 3:1 | -0.555 | -1.454 | 0.345 | 0.601 |
|  | **Like drug effect AUC** | | | |
| 0:1 - 1:1 | 0.836 | -1.376 | 3.048 | 0.872 |
| 0:1 - 2:1 | 2.200 | -0.012 | 4.411 | 0.192 |
| 0:1 - 3:1 | 0.322 | -1.890 | 2.534 | 0.991 |
| 1:1 - 2:1 | 1.364 | -0.848 | 3.575 | 0.602 |
| 1:1 - 3:1 | -0.514 | -2.725 | 1.698 | 0.966 |
| 2:1 - 3:1 | -1.877 | -4.089 | 0.334 | 0.323 |
|  | **Like drug effect AUC (visit included in model)** | | | |
| 0:1 - 1:1 | 0.835 | -1.385 | 3.055 | 0.873 |
| 0:1 - 2:1 | 2.198 | -0.023 | 4.418 | 0.196 |
| 0:1 - 3:1 | 0.331 | -1.902 | 2.563 | 0.991 |
| 1:1 - 2:1 | 1.363 | -0.857 | 3.583 | 0.605 |
| 1:1 - 3:1 | -0.504 | -2.740 | 1.732 | 0.969 |
| 2:1 - 3:1 | -1.867 | -4.105 | 0.371 | 0.338 |
|  | **Like drug effect peak** | | | |
| 0:1 - 1:1 | 0.233 | -0.515 | 0.981 | 0.923 |
| 0:1 - 2:1 | 0.744 | -0.004 | 1.492 | 0.192 |
| 0:1 - 3:1 | 0.273 | -0.475 | 1.021 | 0.882 |
| 1:1 - 2:1 | 0.511 | -0.237 | 1.259 | 0.516 |
| 1:1 - 3:1 | 0.040 | -0.708 | 0.788 | 1.000 |
| 2:1 - 3:1 | -0.471 | -1.219 | 0.277 | 0.585 |

|  | **Like drug effect peak (visit included in model)** | | | |
| --- | --- | --- | --- | --- |
| 0:1 - 1:1 | 0.231 | -0.520 | 0.981 | 0.926 |
| 0:1 - 2:1 | 0.740 | -0.010 | 1.490 | 0.198 |
| 0:1 - 3:1 | 0.294 | -0.460 | 1.049 | 0.861 |
| 1:1 - 2:1 | 0.510 | -0.240 | 1.260 | 0.521 |
| 1:1 - 3:1 | 0.064 | -0.692 | 0.819 | 0.998 |
| 2:1 - 3:1 | -0.446 | -1.202 | 0.310 | 0.636 |
|  | **Mentally impaired AUC** | | | |
| 0:1 - 1:1 | -0.716 | -2.595 | 1.164 | 0.869 |
| 0:1 - 2:1 | -1.612 | -3.491 | 0.268 | 0.314 |
| 0:1 - 3:1 | -2.687 | -4.567 | -0.808 | 0.024 |
| 1:1 - 2:1 | -0.896 | -2.776 | 0.983 | 0.772 |
| 1:1 - 3:1 | -1.972 | -3.851 | -0.092 | 0.154 |
| 2:1 - 3:1 | -1.075 | -2.955 | 0.804 | 0.658 |
|  | **Mentally impaired AUC (visit included in model)** | | | |
| 0:1 - 1:1 | -0.722 | -2.607 | 1.163 | 0.867 |
| 0:1 - 2:1 | -1.621 | -3.507 | 0.264 | 0.311 |
| 0:1 - 3:1 | -2.639 | -4.535 | -0.743 | 0.029 |
| 1:1 - 2:1 | -0.899 | -2.784 | 0.986 | 0.772 |
| 1:1 - 3:1 | -1.917 | -3.816 | -0.018 | 0.181 |
| 2:1 - 3:1 | -1.018 | -2.918 | 0.882 | 0.703 |
|  | **Mentally impaired peak** | | | |
| 0:1 - 1:1 | -0.229 | -0.986 | 0.529 | 0.929 |
| 0:1 - 2:1 | -0.093 | -0.851 | 0.664 | 0.995 |
| 0:1 - 3:1 | -0.804 | -1.562 | -0.047 | 0.146 |
| 1:1 - 2:1 | 0.136 | -0.622 | 0.893 | 0.984 |
| 1:1 - 3:1 | -0.576 | -1.333 | 0.182 | 0.422 |
| 2:1 - 3:1 | -0.711 | -1.469 | 0.046 | 0.237 |
|  | **Mentally impaired peak (visit included in model)** | | | |
| 0:1 - 1:1 | -0.232 | -0.992 | 0.527 | 0.927 |
| 0:1 - 2:1 | -0.099 | -0.858 | 0.661 | 0.994 |
| 0:1 - 3:1 | -0.778 | -1.541 | -0.014 | 0.174 |
| 1:1 - 2:1 | 0.134 | -0.625 | 0.893 | 0.985 |
| 1:1 - 3:1 | -0.545 | -1.310 | 0.219 | 0.479 |
| 2:1 - 3:1 | -0.679 | -1.445 | 0.086 | 0.284 |
|  | **Want alcohol AUC** | | | |
| 0:1 - 1:1 | 0.403 | -1.300 | 2.106 | 0.964 |
| 0:1 - 2:1 | 0.115 | -1.588 | 1.818 | 0.999 |
| 0:1 - 3:1 | 0.357 | -1.346 | 2.060 | 0.975 |
| 1:1 - 2:1 | -0.288 | -1.991 | 1.415 | 0.986 |
| 1:1 - 3:1 | -0.046 | -1.749 | 1.657 | 1.000 |
| 2:1 - 3:1 | 0.242 | -1.461 | 1.945 | 0.992 |
|  | **Want alcohol AUC (visit included in model)** | | | |
| 0:1 - 1:1 | 0.394 | -1.312 | 2.100 | 0.966 |
| 0:1 - 2:1 | 0.101 | -1.605 | 1.807 | 0.999 |
| 0:1 - 3:1 | 0.426 | -1.290 | 2.141 | 0.959 |
| 1:1 - 2:1 | -0.293 | -1.999 | 1.413 | 0.986 |
| 1:1 - 3:1 | 0.032 | -1.687 | 1.750 | 1.000 |
| 2:1 - 3:1 | 0.325 | -1.395 | 2.044 | 0.981 |
|  | **Want alcohol peak** | | | |
| 0:1 - 1:1 | 0.029 | -0.483 | 0.541 | 0.999 |
| 0:1 - 2:1 | -0.296 | -0.807 | 0.216 | 0.651 |
| 0:1 - 3:1 | -0.002 | -0.514 | 0.509 | 1.000 |
| 1:1 - 2:1 | -0.324 | -0.836 | 0.187 | 0.579 |
| 1:1 - 3:1 | -0.031 | -0.543 | 0.481 | 0.999 |
| 2:1 - 3:1 | 0.293 | -0.218 | 0.805 | 0.656 |

|  | **Want alcohol peak (visit included in model)** | | | |
| --- | --- | --- | --- | --- |
| 0:1 - 1:1 | 0.024 | -0.486 | 0.533 | 1.000 |
| 0:1 - 2:1 | -0.303 | -0.813 | 0.206 | 0.629 |
| 0:1 - 3:1 | 0.037 | -0.476 | 0.549 | 0.999 |
| 1:1 - 2:1 | -0.327 | -0.837 | 0.183 | 0.569 |
| 1:1 - 3:1 | 0.013 | -0.500 | 0.526 | 1.000 |
| 2:1 - 3:1 | 0.340 | -0.174 | 0.854 | 0.544 |
|  | **Want food AUC** | | | |
| 0:1 - 1:1 | -0.859 | -3.520 | 1.801 | 0.915 |
| 0:1 - 2:1 | 1.305 | -1.356 | 3.965 | 0.757 |
| 0:1 - 3:1 | -0.978 | -3.639 | 1.682 | 0.880 |
| 1:1 - 2:1 | 2.164 | -0.497 | 4.824 | 0.361 |
| 1:1 - 3:1 | -0.119 | -2.779 | 2.542 | 1.000 |
| 2:1 - 3:1 | -2.283 | -4.943 | 0.378 | 0.313 |
|  | **Want food AUC (visit included in model)** | | | |
| 0:1 - 1:1 | -0.892 | -3.532 | 1.748 | 0.904 |
| 0:1 - 2:1 | 1.256 | -1.385 | 3.896 | 0.774 |
| 0:1 - 3:1 | -0.734 | -3.389 | 1.921 | 0.944 |
| 1:1 - 2:1 | 2.148 | -0.492 | 4.788 | 0.361 |
| 1:1 - 3:1 | 0.158 | -2.502 | 2.817 | 0.999 |
| 2:1 - 3:1 | -1.990 | -4.652 | 0.671 | 0.437 |
|  | **Want food peak** | |  |  |
| 0:1 - 1:1 | -0.407 | -1.307 | 0.494 | 0.800 |
| 0:1 - 2:1 | 0.627 | -0.274 | 1.527 | 0.501 |
| 0:1 - 3:1 | -0.120 | -1.021 | 0.781 | 0.993 |
| 1:1 - 2:1 | -0.747 | -1.647 | 0.154 | 0.344 |
| 1:1 - 3:1 | -0.407 | -1.307 | 0.494 | 0.800 |
| 2:1 - 3:1 | 0.627 | -0.274 | 1.527 | 0.501 |
|  | **Want food peak (visit included in model)** | |  |  |
| 0:1 - 1:1 | -0.302 | -1.186 | 0.582 | 0.901 |
| 0:1 - 2:1 | 0.317 | -0.567 | 1.201 | 0.888 |
| 0:1 - 3:1 | -0.291 | -1.179 | 0.598 | 0.912 |
| 1:1 - 2:1 | 0.619 | -0.265 | 1.503 | 0.495 |
| 1:1 - 3:1 | 0.012 | -0.879 | 0.902 | 1.000 |
| 2:1 - 3:1 | -0.607 | -1.498 | 0.283 | 0.518 |
|  | **Want more drug AUC** | | | |
| 0:1 - 1:1 | -0.340 | -2.658 | 1.978 | 0.991 |
| 0:1 - 2:1 | 1.413 | -0.905 | 3.731 | 0.611 |
| 0:1 - 3:1 | 0.516 | -1.802 | 2.834 | 0.970 |
| 1:1 - 2:1 | 1.753 | -0.565 | 4.071 | 0.427 |
| 1:1 - 3:1 | 0.856 | -1.462 | 3.174 | 0.879 |
| 2:1 - 3:1 | -0.897 | -3.215 | 1.421 | 0.864 |
|  | **Want more drug AUC (visit included in model)** | | | |
| 0:1 - 1:1 | -0.371 | -2.666 | 1.923 | 0.988 |
| 0:1 - 2:1 | 1.366 | -0.929 | 3.660 | 0.629 |
| 0:1 - 3:1 | 0.751 | -1.556 | 3.058 | 0.913 |
| 1:1 - 2:1 | 1.737 | -0.557 | 4.031 | 0.426 |
| 1:1 - 3:1 | 1.122 | -1.188 | 3.433 | 0.762 |
| 2:1 - 3:1 | -0.615 | -2.927 | 1.698 | 0.950 |
|  | **Want more drug peak** | | | |
| 0:1 - 1:1 | -0.029 | -0.856 | 0.798 | 1.000 |
| 0:1 - 2:1 | 0.327 | -0.501 | 1.154 | 0.856 |
| 0:1 - 3:1 | 0.064 | -0.763 | 0.892 | 0.999 |
| 1:1 - 2:1 | 0.356 | -0.472 | 1.183 | 0.823 |
| 1:1 - 3:1 | 0.093 | -0.734 | 0.921 | 0.996 |
| 2:1 - 3:1 | -0.262 | -1.090 | 0.565 | 0.919 |

|  | **Want more drug peak (visit included in model)** | | | |
| --- | --- | --- | --- | --- |
| 0:1 - 1:1 | -0.052 | -0.830 | 0.725 | 0.999 |
| 0:1 - 2:1 | 0.291 | -0.486 | 1.069 | 0.875 |
| 0:1 - 3:1 | 0.241 | -0.540 | 1.023 | 0.925 |
| 1:1 - 2:1 | 0.344 | -0.434 | 1.121 | 0.810 |
| 1:1 - 3:1 | 0.294 | -0.489 | 1.077 | 0.874 |
| 2:1 - 3:1 | -0.050 | -0.834 | 0.734 | 0.999 |

**eFigure 10** Pleasurable response ratings for chocolate and music stratified by CBD:THC ratios. Circles show individual data points, diamonds show mean values, boxplots show median and interquartile range and half violin plots show the distribution of participants.

**A** Chocolate **B** Music


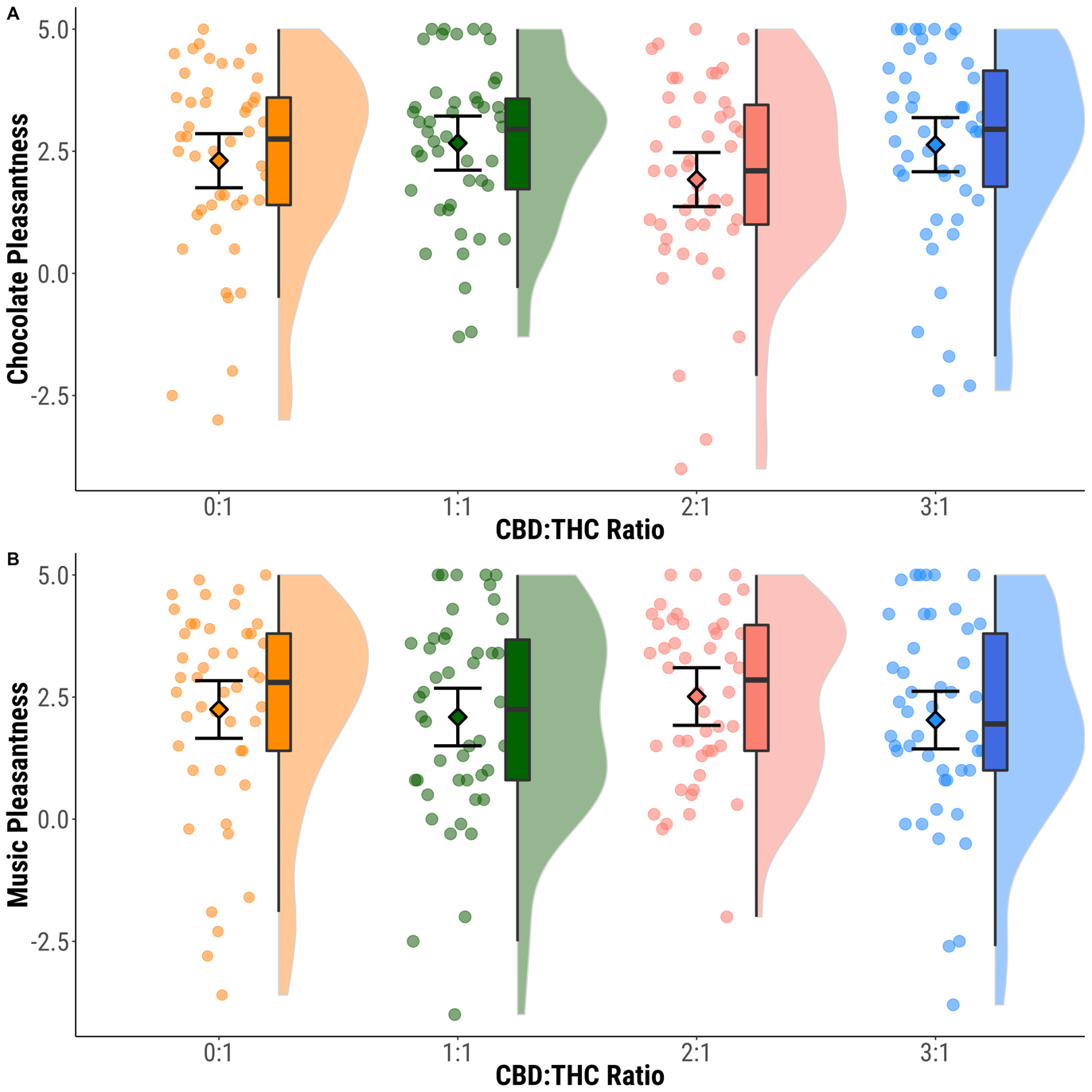


| **eTable 13** Pleasurable response ratings for chocolate and music stratified by CBD:THC ratio | | | | | |
| --- | --- | --- | --- | --- | --- |
|  |  | **CBD:THC Ratio** | | | |
|  |  | **0:1** | **1:1** | **2:1** | **3:1** |
| **Chocolate** | **Mean (SD)** | 2.31 (1.93) | 2.67 (1.64) | 1.92 (2.03) | 2.63 (1.98) |
| **Significant differences?** |  | No | No | No | No |
| **Music** | **Mean (SD)** | 2.25 (2.14) | 2.09 (2.11) | 2.51 (1.71) | 2.03 (2.10) |
| **Significant differences?** |  | No | No | No | No |

| **eTable 14** Results of linear mixed models of pleasurable response ratings for each contrast between CBD:THC ratios. Statistically significant rows are presented in bold. CI, confidence interval; EMM, estimated marginal mean. | | | | |
| --- | --- | --- | --- | --- |
|  |  | | | |
| **Contrast** | **EMM difference** | **Lower 95% CI** | **Upper 95% CI** | **p-value** |
|  | **Chocolate** |  |  |  |
| 0:1 - 1:1 | -0.361 | -1.045 | 0.324 | 0.713 |
| 0:1 - 2:1 | 0.385 | -0.300 | 1.069 | 0.670 |
| 0:1 - 3:1 | -0.328 | -1.013 | 0.356 | 0.769 |
| 1:1 - 2:1 | 0.746 | 0.061 | 1.430 | 0.130 |
| 1:1 - 3:1 | 0.033 | -0.652 | 0.717 | 1.000 |
| 2:1 - 3:1 | -0.713 | -1.397 | -0.029 | 0.159 |
|  | **Chocolate (visit included in model)** | | | |
| 0:1 - 1:1 | -0.368 | -1.054 | 0.318 | 0.702 |
| 0:1 - 2:1 | 0.374 | -0.312 | 1.060 | 0.691 |
| 0:1 - 3:1 | -0.304 | -0.992 | 0.385 | 0.811 |
| 1:1 - 2:1 | 0.742 | 0.057 | 1.428 | 0.134 |
| 1:1 - 3:1 | 0.064 | -0.627 | 0.755 | 0.998 |
| 2:1 - 3:1 | -0.678 | -1.370 | 0.014 | 0.203 |
|  | **Music** | | | |
| 0:1 - 1:1 | 0.154 | -0.546 | 0.854 | 0.971 |
| 0:1 - 2:1 | -0.265 | -0.965 | 0.435 | 0.871 |
| 0:1 - 3:1 | 0.217 | -0.483 | 0.917 | 0.924 |
| 1:1 - 2:1 | -0.420 | -1.120 | 0.280 | 0.623 |
| 1:1 - 3:1 | 0.063 | -0.637 | 0.763 | 0.998 |
| 2:1 - 3:1 | 0.483 | -0.217 | 1.183 | 0.509 |
|  | **Music (visit included in model)** | | | |
| 0:1 - 1:1 | 0.166 | -0.534 | 0.865 | 0.964 |
| 0:1 - 2:1 | -0.248 | -0.948 | 0.451 | 0.891 |
| 0:1 - 3:1 | 0.178 | -0.524 | 0.880 | 0.957 |
| 1:1 - 2:1 | -0.414 | -1.113 | 0.285 | 0.633 |
| 1:1 - 3:1 | 0.012 | -0.692 | 0.717 | 1.000 |
| 2:1 - 3:1 | 0.426 | -0.280 | 1.132 | 0.618 |

**
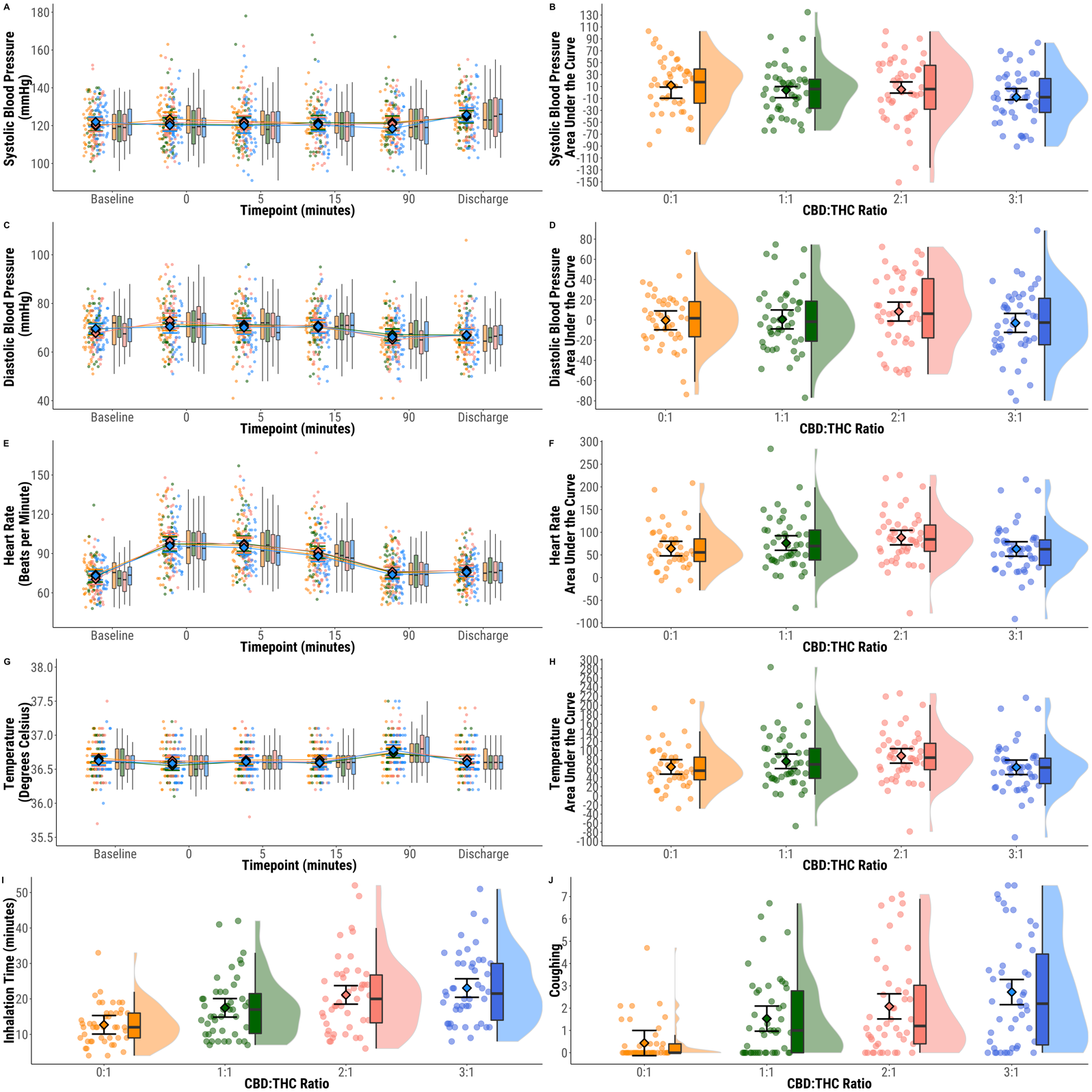
eFigure 11** Physiological outcomes stratified by CBD:THC ratio **A** Systolic blood pressure **B** Systolic blood pressure AUC **C** Diastolic blood pressure **D** Diastolic blood pressure AUC **E** Heart rate **F** Heart rate AUC **G** Body temperature **H** Body temperature AUC **I** Inhalation time **J** Coughing. Circles show individual data points, diamonds show mean values, boxplots show median and interquartile range and half violin plots show the distribution of participants.

2:1

1:1

0:1

3:1

| **eTable 15** Physiology stratified by CBD:THC ratio. All values in peak effects and AUC analyses were baseline corrected | | | | | |
| --- | --- | --- | --- | --- | --- |
|  |  | **CBD:THC Ratio** | | | |
|  |  | **0:1** | **1:1** | **2:1** | **3:1** |
| **Outcome** | **Systolic blood pressure (mmHg)** | | | | |
| **Baseline** | **Mean (SD)** | 119.83 (9.85) | 120.57 (11.40) | 120.33 (11.66) | 121.98 (8.86) |
| **0 minutes** | **Mean (SD)** | 123.52 (12.29) | 120.80 (11.95) | 121.59 (12.54) | 120.20 (10.70) |
| **5 minutes** | **Mean (SD)** | 121.70 (10.33) | 120.39 (14.58) | 121.63 (12.11) | 119.98 (13.47) |
| **15 minutes** | **Mean (SD)** | 121.39 (9.74) | 121.61 (12.81) | 120.61 (12.52) | 120.30 (10.95) |
| **90 minutes** | **Mean (SD)** | 122.02 (9.74) | 121.85 (13.06) | 120.09 (10.69) | 118.43 (10.94) |
| **Discharge** | **Mean (SD)** | 124.76 (10.16) | 124.28 (11.25) | 125.50 (11.67) | 125.76 (12.34) |
|  | **Diastolic blood pressure (mmHg)** | | | | |
| **Baseline** | **Mean (SD)** | 69.28 (8.07) | 69.54 (7.50) | 67.80 (6.92) | 69.63 (6.44) |
| **0 minutes** | **Mean (SD)** | 71.54 (9.11) | 70.50 (8.95) | 72.91 (10.39) | 70.46 (8.83) |
| **5 minutes** | **Mean (SD)** | 70.30 (9.58) | 71.35 (8.83) | 70.85 (8.49) | 70.07 (9.15) |
| **15 minutes** | **Mean (SD)** | 69.96 (7.50) | 70.72 (9.44) | 70.98 (9.10) | 70.43 (9.15) |
| **90 minutes** | **Mean (SD)** | 66.02 (9.37) | 67.50 (7.79) | 64.87 (7.34) | 66.11 (7.97) |
| **Discharge** | **Mean (SD)** | 66.63 (9.77) | 66.96 (7.75) | 67.02 (7.88) | 66.96 (7.71) |
|  | **Heart rate (beats per minute)** | | | | |
| **Baseline** | **Mean (SD)** | 73.91 (13.44) | 72.43 (14.24) | 70.43 (13.53) | 73.11 (10.94) |
| **0 minutes** | **Mean (SD)** | 97.37 (20.60) | 97.28 (18.47) | 99.54 (19.95) | 95.89 (16.40) |
| **5 minutes** | **Mean (SD)** | 95.54 (19.15) | 97.54 (19.71) | 96.74 (19.83) | 94.48 (17.29) |
| **15 minutes** | **Mean (SD)** | 88.87 (16.59) | 90.96 (15.73) | 91.50 (22.03) | 88.07 (14.14) |
| **90 minutes** | **Mean (SD)** | 75.17 (14.55) | 75.85 (14.04) | 77.11 (13.51) | 73.89 (12.13) |
| **Discharge** | **Mean (SD)** | 75.37 (11.20) | 77.22 (12.99) | 77.17 (12.53) | 75.80 (10.87) |
|  | **Body temperature (°Celsius)** | | | | |
| **Baseline** | **Mean (SD)** | 36.66 (0.23) | 36.63 (0.24) | 36.63 (0.29) | 36.62 (0.21) |
| **0 minutes** | **Mean (SD)** | 36.60 (0.24) | 36.55 (0.23) | 36.63 (0.21) | 36.58 (0.23) |
| **5 minutes** | **Mean (SD)** | 36.63 (0.20) | 36.61 (0.19) | 36.61 (0.26) | 36.61 (0.20) |
| **15 minutes** | **Mean (SD)** | 36.64 (0.20) | 36.59 (0.19) | 36.61 (0.20) | 36.60 (0.20) |
| **90 minutes** | **Mean (SD)** | 36.75 (0.26) | 36.73 (0.22) | 36.78 (0.25) | 36.78 (0.26) |
| **Discharge** | **Mean (SD)** | 36.64 (0.25) | 36.65 (0.22) | 36.64 (0.24) | 36.59 (0.21) |
|  | **Inhalation time (minutes)** | | | | |
|  | **Mean (SD)** | 12.7 (5.55) | 17.5 (8.62) | 21.1 (10.62) | 23.1 (9.97) |
|  | **Coughing (0, least severe – 10, most severe)** | | | | |
|  | **Mean (SD)** | 0.42 (0.87) | 1.63 (1.84) | 2.08 (2.19) | 2.86 (2.50) |

| **eTable 16** Results of linear mixed models of physiological measures for each contrast between CBD:THC ratios. Statistically significant rows are presented in bold. AUC, area under the curve; CI, confidence interval; EMM, estimated marginal mean. | | | | |
| --- | --- | --- | --- | --- |
|  |  | | | |
| **Contrast** | **EMM difference** | **Lower 95% CI** | **Upper 95% CI** | **p-value** |
|  | **Systolic blood pressure AUC** |  |  |  |
| 0:1 - 1:1 | 8.396 | -8.179 | 24.972 | 0.738 |
| 0:1 - 2:1 | 6.974 | -9.602 | 23.549 | 0.832 |
| 0:1 - 3:1 | 20.443 | 3.868 | 37.019 | 0.067 |
| 1:1 - 2:1 | -1.423 | -17.998 | 15.152 | 0.998 |
| 1:1 - 3:1 | 12.047 | -4.528 | 28.622 | 0.462 |
| 2:1 - 3:1 | 13.470 | -3.106 | 30.045 | 0.362 |
|  | **Systolic blood pressure AUC (visit included in model)** | | | |
| 0:1 - 1:1 | 8.629 | -7.950 | 25.209 | 0.721 |
| 0:1 - 2:1 | 7.323 | -9.265 | 23.910 | 0.811 |
| 0:1 - 3:1 | 19.629 | 2.978 | 36.281 | 0.087 |
| 1:1 - 2:1 | -1.306 | -17.881 | 15.268 | 0.999 |
| 1:1 - 3:1 | 11.000 | -5.702 | 27.703 | 0.548 |
| 2:1 - 3:1 | 12.307 | -4.426 | 29.039 | 0.452 |
|  | **Systolic blood pressure peak** | | | |
| 0:1 - 1:1 | 3.457 | -0.733 | 7.646 | 0.348 |
| 0:1 - 2:1 | 2.435 | -1.755 | 6.625 | 0.646 |
| 0:1 - 3:1 | 5.478 | 1.289 | 9.668 | 0.046 |
| 1:1 - 2:1 | -1.022 | -5.211 | 3.168 | 0.961 |
| 1:1 - 3:1 | 2.022 | -2.168 | 6.211 | 0.766 |
| 2:1 - 3:1 | 3.043 | -1.146 | 7.233 | 0.463 |
|  | **Systolic blood pressure peak (visit included in model)** | | | |
| 0:1 - 1:1 | 3.511 | -0.682 | 7.704 | 0.335 |
| 0:1 - 2:1 | 2.516 | -1.679 | 6.712 | 0.623 |
| 0:1 - 3:1 | 5.288 | 1.076 | 9.499 | 0.060 |
| 1:1 - 2:1 | -0.995 | -5.186 | 3.197 | 0.964 |
| 1:1 - 3:1 | 1.777 | -2.447 | 6.001 | 0.832 |
| 2:1 - 3:1 | 2.771 | -1.460 | 7.003 | 0.553 |
|  | **Diastolic blood pressure AUC** |  |  |  |
| 0:1 - 1:1 | -0.606 | -12.835 | 11.622 | 1.000 |
| 0:1 - 2:1 | -8.620 | -20.849 | 3.609 | 0.489 |
| 0:1 - 3:1 | 2.828 | -9.400 | 15.057 | 0.966 |
| 1:1 - 2:1 | -8.014 | -20.243 | 4.215 | 0.552 |
| 1:1 - 3:1 | 3.435 | -8.794 | 15.664 | 0.942 |
| 2:1 - 3:1 | 11.449 | -0.780 | 23.677 | 0.239 |
|  | **Diastolic blood pressure AUC (visit included in model)** | | | |
| 0:1 - 1:1 | -0.565 | -12.841 | 11.711 | 1.000 |
| 0:1 - 2:1 | -8.558 | -20.841 | 3.724 | 0.499 |
| 0:1 - 3:1 | 2.684 | -9.646 | 15.014 | 0.972 |
| 1:1 - 2:1 | -7.993 | -20.266 | 4.280 | 0.557 |
| 1:1 - 3:1 | 3.249 | -9.118 | 15.616 | 0.952 |
| 2:1 - 3:1 | 11.242 | -1.148 | 23.632 | 0.265 |
|  | **Diastolic blood pressure peak** | | | |
| 0:1 - 1:1 | 1.304 | -1.814 | 4.423 | 0.834 |
| 0:1 - 2:1 | -2.848 | -5.966 | 0.270 | 0.260 |
| 0:1 - 3:1 | 1.435 | -1.684 | 4.553 | 0.791 |
| 1:1 - 2:1 | -4.152 | -7.270 | -1.034 | 0.041 |
| 1:1 - 3:1 | 0.130 | -2.988 | 3.249 | 1.000 |
| 2:1 - 3:1 | 4.283 | 1.164 | 7.401 | 0.032 |

|  | **Diastolic blood pressure peak (visit included in model)** | | | |
| --- | --- | --- | --- | --- |
| 0:1 - 1:1 | 1.332 | -1.794 | 4.459 | 0.826 |
| 0:1 - 2:1 | -2.806 | -5.934 | 0.322 | 0.275 |
| 0:1 - 3:1 | 1.337 | -1.803 | 4.476 | 0.827 |
| 1:1 - 2:1 | -4.138 | -7.263 | -1.013 | 0.042 |
| 1:1 - 3:1 | 0.004 | -3.145 | 3.154 | 1.000 |
| 2:1 - 3:1 | 4.142 | 0.987 | 7.298 | 0.045 |
|  | **Heart rate AUC** |  |  |  |
| 0:1 - 1:1 | -12.381 | -30.537 | 5.775 | 0.518 |
| 0:1 - 2:1 | -24.781 | -42.937 | -6.625 | 0.034 |
| 0:1 - 3:1 | 0.630 | -17.526 | 18.786 | 1.000 |
| 1:1 - 2:1 | -12.400 | -30.556 | 5.756 | 0.517 |
| 1:1 - 3:1 | 13.011 | -5.145 | 31.167 | 0.475 |
| 2:1 - 3:1 | 25.411 | 7.255 | 43.567 | 0.028 |
|  | **Heart rate AUC (visit included in model)** | | | |
| 0:1 - 1:1 | -12.099 | -30.244 | 6.046 | 0.537 |
| 0:1 - 2:1 | -24.357 | -42.511 | -6.204 | 0.038 |
| 0:1 - 3:1 | -0.357 | -18.581 | 17.866 | 1.000 |
| 1:1 - 2:1 | -12.259 | -30.398 | 5.881 | 0.526 |
| 1:1 - 3:1 | 11.741 | -6.538 | 30.021 | 0.569 |
| 2:1 - 3:1 | 24.000 | 5.687 | 42.313 | 0.045 |
|  | **Heart rate peak** | | | |
| 0:1 - 1:1 | -1.391 | -6.094 | 3.312 | 0.933 |
| 0:1 - 2:1 | -5.652 | -10.355 | -0.949 | 0.078 |
| 0:1 - 3:1 | 0.674 | -4.029 | 5.377 | 0.992 |
| 1:1 - 2:1 | -4.261 | -8.964 | 0.442 | 0.266 |
| 1:1 - 3:1 | 2.065 | -2.638 | 6.768 | 0.813 |
| 2:1 - 3:1 | 6.326 | 1.623 | 11.029 | 0.038 |
|  | **Heart rate peak (visit included in model)** | | | |
| 0:1 - 1:1 | -1.317 | -6.016 | 3.383 | 0.942 |
| 0:1 - 2:1 | -5.541 | -10.242 | -0.839 | 0.087 |
| 0:1 - 3:1 | 0.414 | -4.306 | 5.133 | 0.998 |
| 1:1 - 2:1 | -4.224 | -8.922 | 0.474 | 0.273 |
| 1:1 - 3:1 | 1.730 | -3.004 | 6.465 | 0.882 |
| 2:1 - 3:1 | 5.954 | 1.211 | 10.697 | 0.060 |
|  | **Temperature AUC** |  |  |  |
| 0:1 - 1:1 | -0.637 | -12.531 | 11.256 | 1.000 |
| 0:1 - 2:1 | -8.582 | -20.476 | 3.312 | 0.469 |
| 0:1 - 3:1 | 2.605 | -9.289 | 14.499 | 0.971 |
| 1:1 - 2:1 | -7.944 | -19.838 | 3.949 | 0.536 |
| 1:1 - 3:1 | 3.242 | -8.651 | 15.136 | 0.947 |
| 2:1 - 3:1 | 11.187 | -0.707 | 23.081 | 0.235 |
|  | **Temperature AUC (visit included in model)** | | | |
| 0:1 - 1:1 | -0.596 | -12.536 | 11.344 | 1.000 |
| 0:1 - 2:1 | -8.519 | -20.465 | 3.426 | 0.479 |
| 0:1 - 3:1 | 2.459 | -9.532 | 14.451 | 0.976 |
| 1:1 - 2:1 | -7.924 | -19.860 | 4.013 | 0.541 |
| 1:1 - 3:1 | 3.055 | -8.973 | 15.083 | 0.956 |
| 2:1 - 3:1 | 10.979 | -1.071 | 23.029 | 0.262 |
|  | **Temperature peak** | | | |
| 0:1 - 1:1 | 0.015 | -0.105 | 0.135 | 0.994 |
| 0:1 - 2:1 | -0.061 | -0.181 | 0.059 | 0.738 |
| 0:1 - 3:1 | -0.026 | -0.146 | 0.094 | 0.972 |
| 1:1 - 2:1 | -0.076 | -0.196 | 0.044 | 0.580 |
| 1:1 - 3:1 | -0.041 | -0.161 | 0.079 | 0.900 |
| 2:1 - 3:1 | 0.035 | -0.085 | 0.155 | 0.937 |

|  | **Temperature peak (visit included in model)** | | | |
| --- | --- | --- | --- | --- |
| 0:1 - 1:1 | 0.016 | -0.105 | 0.136 | 0.994 |
| 0:1 - 2:1 | -0.060 | -0.181 | 0.061 | 0.748 |
| 0:1 - 3:1 | -0.028 | -0.149 | 0.093 | 0.967 |
| 1:1 - 2:1 | -0.076 | -0.196 | 0.045 | 0.586 |
| 1:1 - 3:1 | -0.044 | -0.165 | 0.078 | 0.888 |
| 2:1 - 3:1 | 0.032 | -0.090 | 0.154 | 0.951 |
|  | **Inhalation** | | | |
| **0:1 - 1:1** | **-4.783** | **-6.934** | **-2.631** | **9.31x10^-5^** |
| **0:1 - 2:1** | **-8.435** | **-10.586** | **-6.283** | **5.34x10^-12^** |
| **0:1 - 3:1** | **-10.370** | **-12.521** | **-8.218** | **2.96x10^-14^** |
| **1:1 - 2:1** | **-3.652** | **-5.804** | **-1.501** | **0.005** |
| **1:1 - 3:1** | **-5.587** | **-7.738** | **-3.436** | **3.74x10^-6^** |
| 2:1 - 3:1 | -1.935 | -4.086 | 0.217 | 0.273 |
|  | **Inhalation (visit included in model)** | | | |
| **0:1 - 1:1** | **-4.783** | **-6.943** | **-2.623** | **1.00x10^-4^** |
| **0:1 - 2:1** | **-8.435** | **-10.596** | **-6.274** | **6.70x10^-12^** |
| **0:1 - 3:1** | **-10.369** | **-12.538** | **-8.199** | **2.58x10^-14^** |
| **1:1 - 2:1** | **-3.652** | **-5.812** | **-1.493** | **0.005** |
| **1:1 - 3:1** | **-5.586** | **-7.762** | **-3.410** | **4.94x10^-6^** |
| 2:1 - 3:1 | -1.934 | -4.114 | 0.247 | 0.284 |
|  | **Coughing** | | | |
| **0:1 - 1:1** | **-1.141** | **-1.704** | **-0.536** | **0.001** |
| **0:1 - 2:1** | **-1.689** | **-2.241** | **-1.072** | **4.36x10^-7^** |
| **0:1 - 3:1** | **-2.430** | **-3.093** | **-1.920** | **1.04x10^-12^** |
| 1:1 - 2:1 | -0.548 | -1.121 | 0.047 | 0.256 |
| **1:1 - 3:1** | **-1.289** | **-1.975** | **-0.799** | **1.56x10^-4^** |
| 2:1 - 3:1 | -0.741 | -1.439 | -0.261 | 0.064 |
|  | **Coughing (visit included in model)** | | | |
| **0:1 - 1:1** | **-1.120** | **-1.704** | **-0.536** | **9.94x10^-4^** |
| **0:1 - 2:1** | **-1.657** | **-2.241** | **-1.072** | **4.14x10^-7^** |
| **0:1 - 3:1** | **-2.507** | **-3.093** | **-1.920** | **1.32x10^-13^** |
| 1:1 - 2:1 | -0.537 | -1.121 | 0.047 | 0.254 |
| **1:1 - 3:1** | **-1.387** | **-1.975** | **-0.799** | **3.06x10^-5^** |
| 2:1 - 3:1 | -0.850 | -1.439 | -0.261 | 0.022 |

**eFigure 12** Correlation matrices comparing peak THC and CBD, THC and CBD AUC and subjective high with inhalation time in **A** 0:1 **B** 1:1 **C** 2:1 **D** 3:1. *p<0.008, **p<0.001, ***p<0.0001


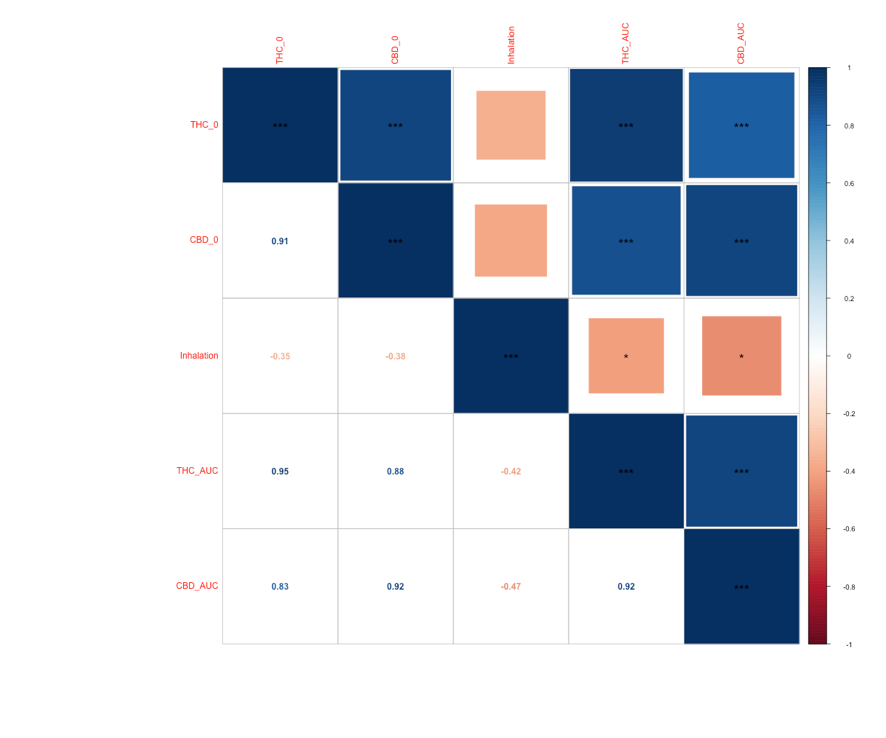

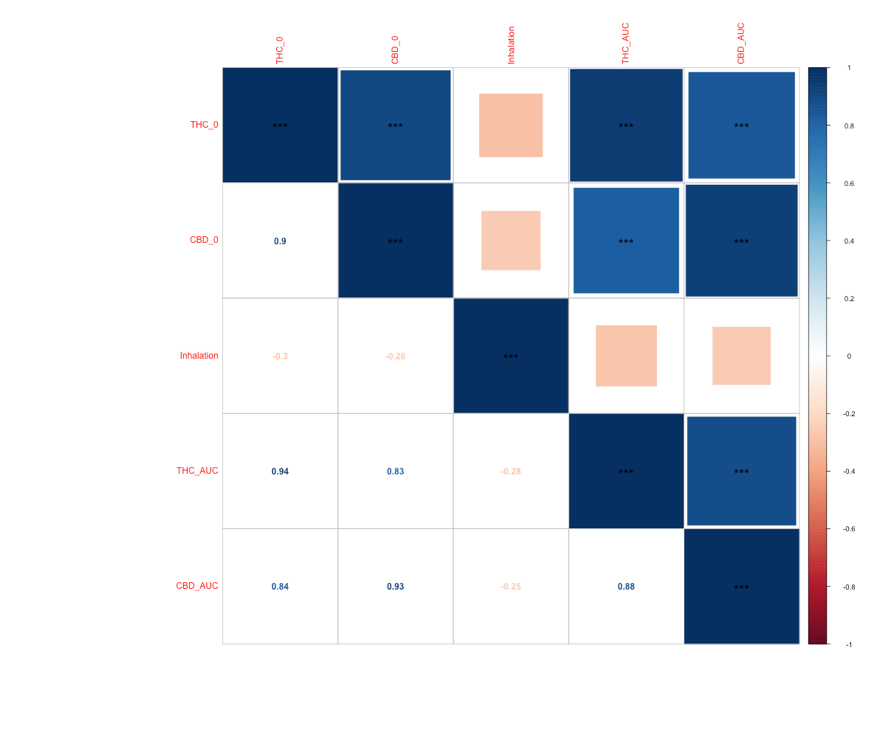

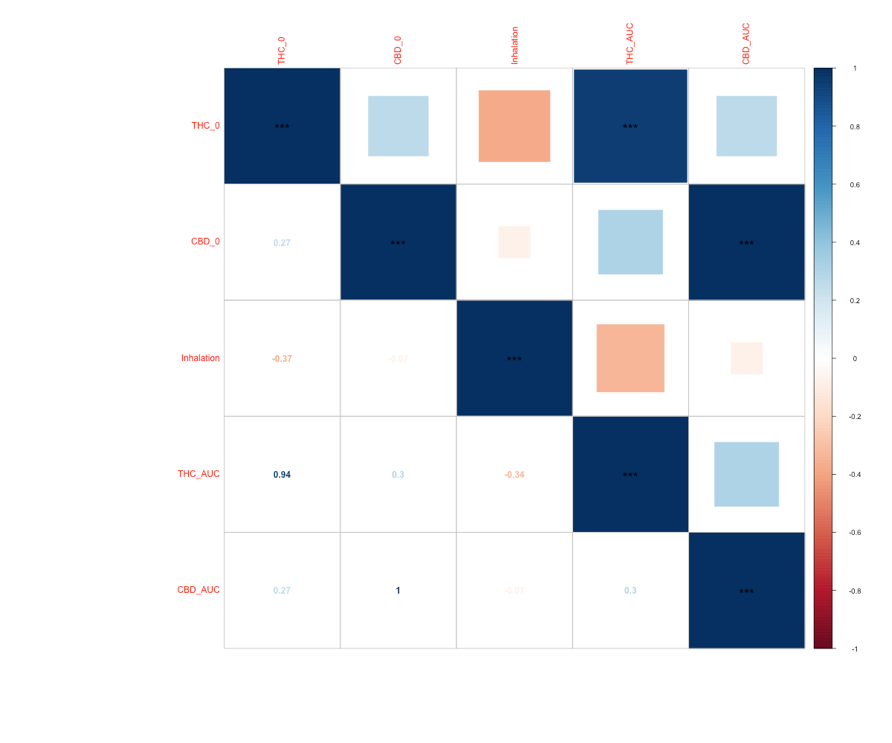


**A**

**B**


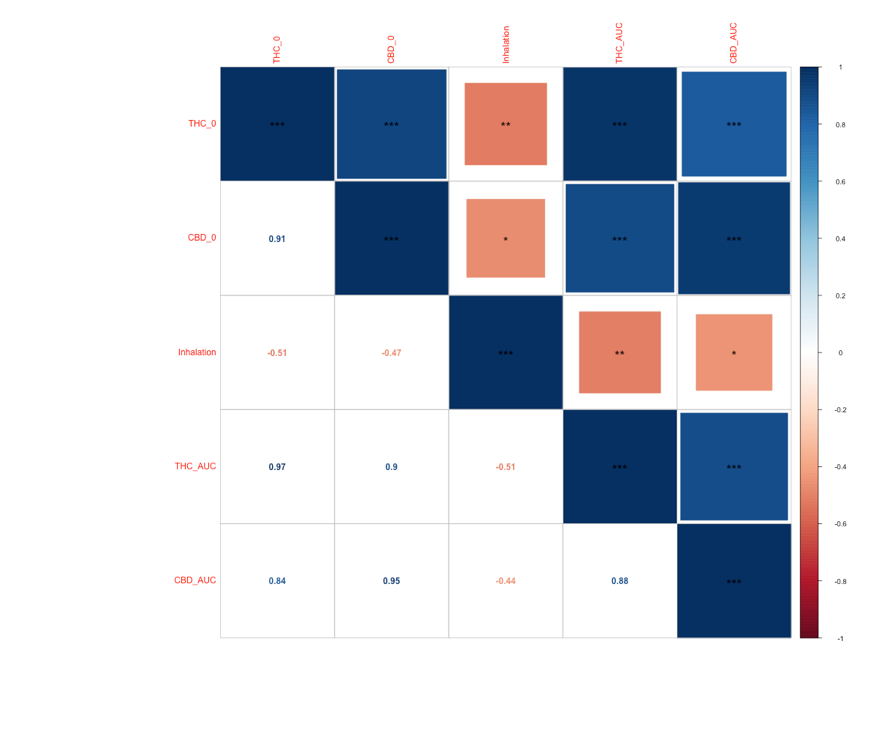


**C**

**D**


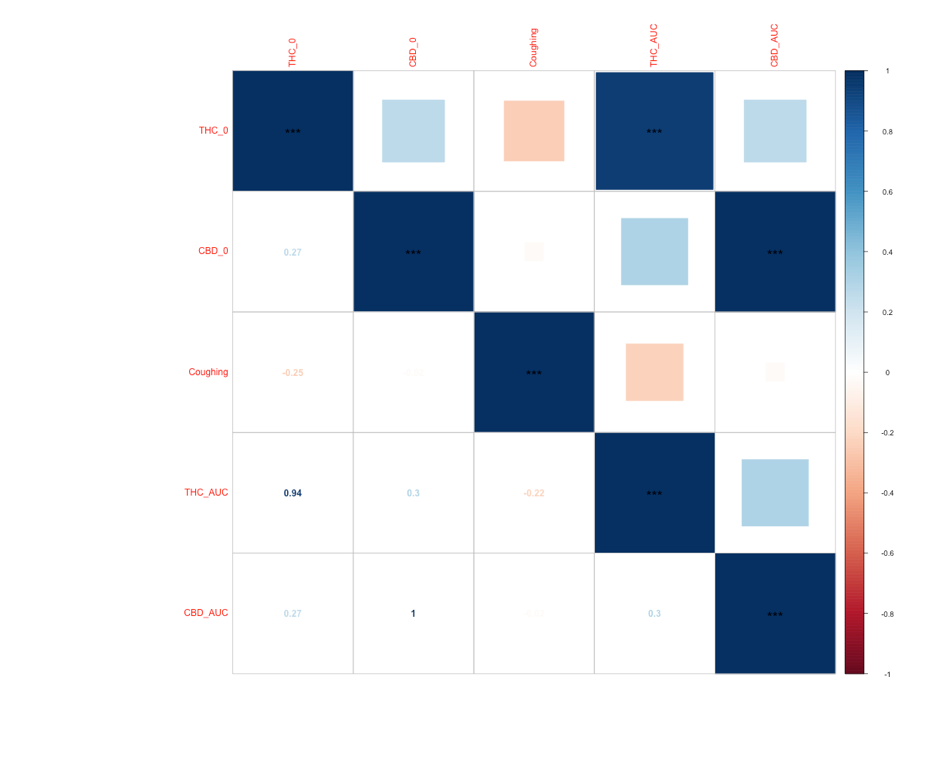
**eFigure 13** Correlation matrices comparing peak THC and CBD, THC and CBD AUC and subjective high with coughing in **A** 0:1 **B** 1:1 **C** 2:1 **D** 3:1. *p<0.008, **p<0.001, ***p<0.0001


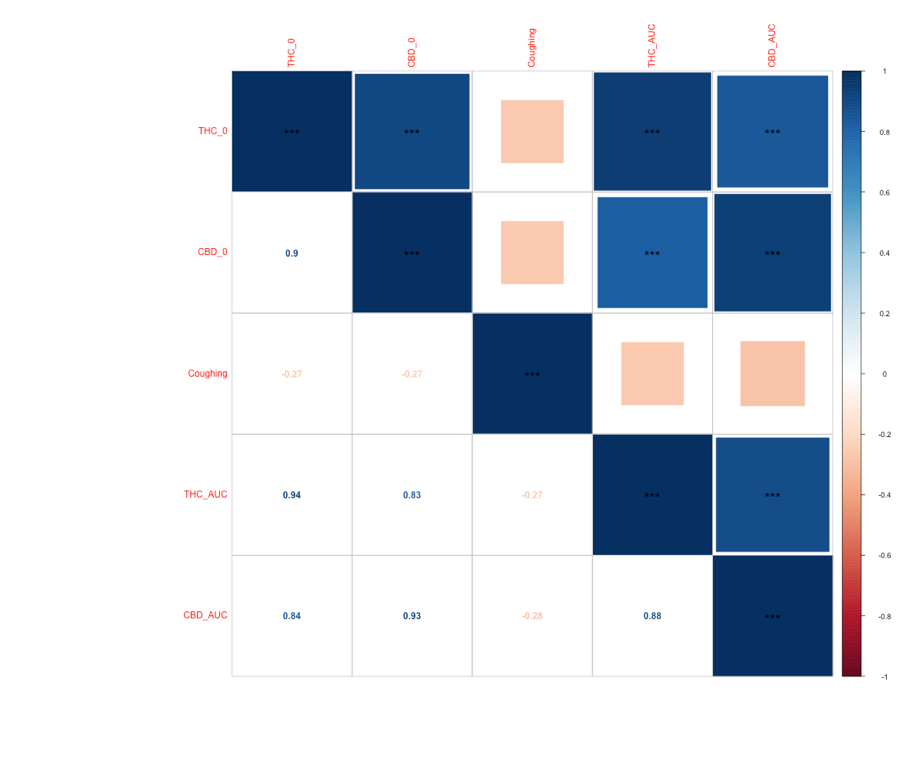

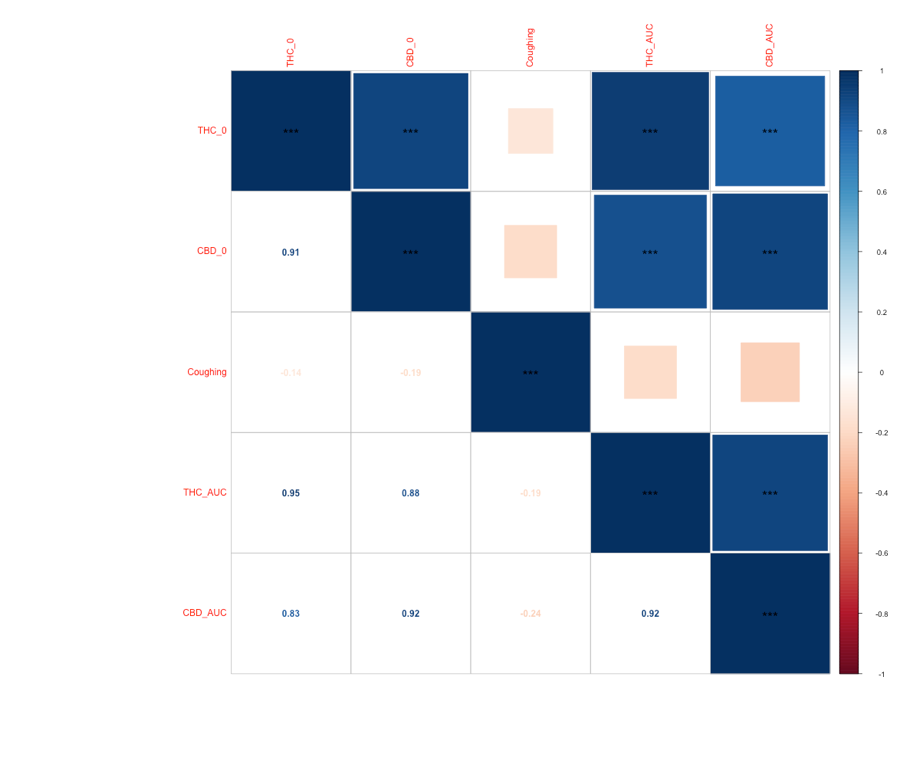


**A**

**B**


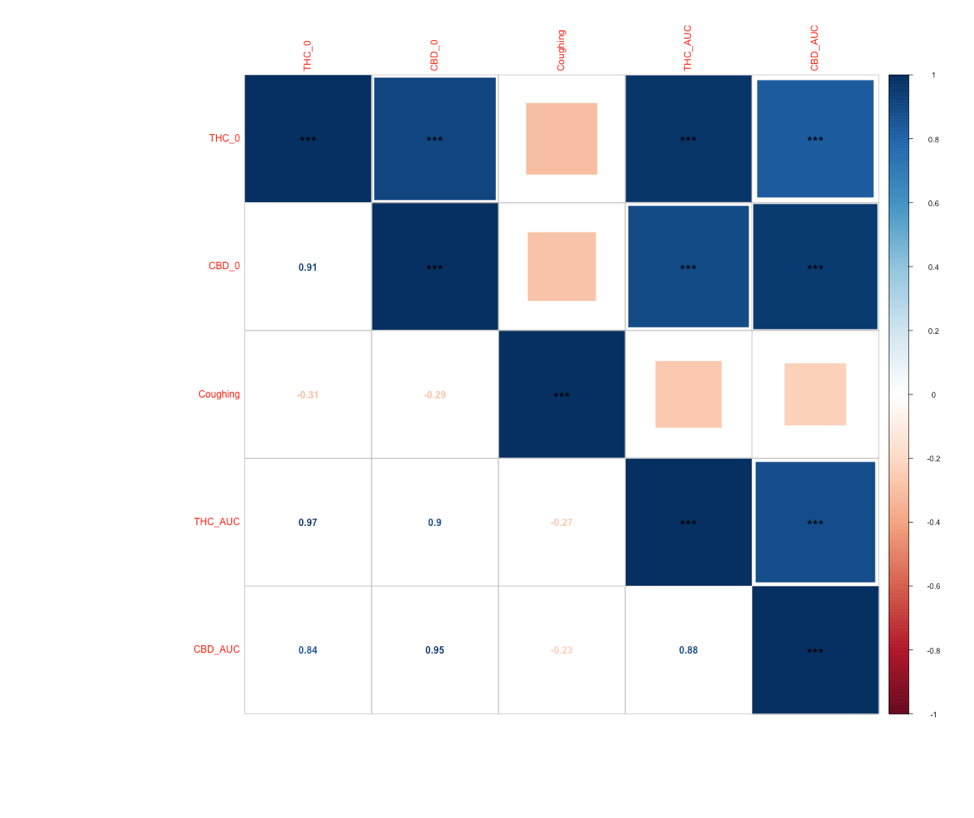


**D**

**C**

**eTable 17** Delayed recall performance on the HVLT-R for Visit 1 across ratios compared with all visits combined

| **CBD (n Visit 1)** | **Mean (Visit 1 only)** | **SE (Visit 1 only)** | **Mean (All visits)** | **SE (All visits)** |
| --- | --- | --- | --- | --- |
| 0:1 (n=11) | 9.45 | 0.612 | 9.41 | 0.314 |
| 1:1 (n=14) | 9.29 | 0.542 | 9.26 | 0.314 |
| 2:1 (n=14) | 8.57 | 0.542 | 9.09 | 0.314 |
| 3:1 (n=7) | 10.00 | 0.767 | 9.39 | 0.314 |

| **eTable 18** Gender differences across ratios on all outcomes | | | | |
| --- | --- | --- | --- | --- |
| **Contrast** | **EMM difference** | **Lower 95% CI** | **Upper 95% CI** | **p-value** |
|  | **THC AUC** |  |  |  |
| Female 0:1 - Male 0:1 | -41.306 | -84.324 | 1.712 | 0.533 |
| Female 0:1 - Female 1:1 | -17.924 | -43.611 | 7.763 | 0.853 |
| Female 0:1 - Female 2:1 | -19.002 | -44.689 | 6.684 | 0.812 |
| Female 0:1 - Female 3:1 | -19.536 | -45.223 | 6.151 | 0.789 |
| Male 0:1 - Male 1:1 | -13.162 | -36.704 | 10.381 | 0.950 |
| Male 0:1 - Male 2:1 | 3.912 | -19.631 | 27.454 | 1.000 |
| Male 0:1 - Male 3:1 | 2.772 | -20.771 | 26.314 | 1.000 |
| Female 1:1 - Male 1:1 | -36.544 | -79.562 | 6.475 | 0.680 |
| Female 1:1 - Female 2:1 | -1.078 | -26.765 | 24.608 | 1.000 |
| Female 1:1 - Female 3:1 | -1.612 | -27.299 | 24.074 | 1.000 |
| Male 1:1 - Male 2:1 | 17.073 | -6.469 | 40.615 | 0.827 |
| Male 1:1 - Male 3:1 | 15.933 | -7.609 | 39.476 | 0.872 |
| Female 2:1 - Male 2:1 | -18.392 | -61.411 | 24.626 | 0.989 |
| Female 2:1 - Female 3:1 | -0.534 | -26.221 | 25.153 | 1.000 |
| Male 2:1 - Male 3:1 | -1.140 | -24.682 | 22.403 | 1.000 |
| Female 3:1 - Male 3:1 | -18.998 | -62.016 | 24.020 | 0.986 |
|  | **THC Peak** |  |  |  |
| Female 0:1 - Male 0:1 | -27.809 | -53.006 | -2.612 | 0.348 |
| Female 0:1 - Female 1:1 | -8.279 | -26.043 | 9.485 | 0.982 |
| Female 0:1 - Female 2:1 | -10.297 | -28.062 | 7.467 | 0.940 |
| Female 0:1 - Female 3:1 | -11.812 | -29.576 | 5.952 | 0.882 |
| Male 0:1 - Male 1:1 | -10.075 | -26.357 | 6.206 | 0.916 |
| Male 0:1 - Male 2:1 | 8.493 | -7.788 | 24.774 | 0.965 |
| Male 0:1 - Male 3:1 | 8.058 | -8.223 | 24.339 | 0.974 |
| Female 1:1 - Male 1:1 | -29.606 | -54.803 | -4.408 | 0.270 |
| Female 1:1 - Female 2:1 | -2.018 | -19.783 | 15.746 | 1.000 |
| Female 1:1 - Female 3:1 | -3.533 | -21.297 | 14.231 | 1.000 |
| Male 1:1 - Male 2:1 | 18.569 | 2.288 | 34.850 | 0.303 |
| Male 1:1 - Male 3:1 | 18.133 | 1.852 | 34.414 | 0.333 |
| Female 2:1 - Male 2:1 | -9.018 | -34.216 | 16.179 | 0.996 |
| Female 2:1 - Female 3:1 | -1.514 | -19.278 | 16.250 | 1.000 |
| Male 2:1 - Male 3:1 | -0.436 | -16.717 | 15.845 | 1.000 |
| Female 3:1 - Male 3:1 | -7.940 | -33.137 | 17.258 | 0.998 |
|  | **CBD AUC** |  |  |  |
| Female 0:1 - Male 0:1 | -0.230 | -81.878 | 81.417 | 1 |
| Female 0:1 - Female 1:1 | -107.960 | -174.811 | -41.108 | 0.031 |
| **Female 0:1 - Female 2:1** | **-222.557** | **-289.408** | **-155.705** | **1.49x10^-8^** |
| **Female 0:1 - Female 3:1** | **-312.700** | **-379.552** | **-245.848** | **5.88x10^-14^** |
| **Male 0:1 - Male 1:1** | **-136.938** | **-198.209** | **-75.668** | **3.83x10^-4^** |
| **Male 0:1 - Male 2:1** | **-262.605** | **-323.875** | **-201.334** | **5.22x10^-13^** |
| Male 0:1 - Male 3:1 | -372.199 | -433.469 | -310.928 | 0 |
| Female 1:1 - Male 1:1 | -29.209 | -110.857 | 52.439 | 0.996 |
| Female 1:1 - Female 2:1 | -114.597 | -181.449 | -47.746 | 0.017 |
| **Female 1:1 - Female 3:1** | **-204.740** | **-271.592** | **-137.889** | **2.18x10^-7^** |
| **Male 1:1 - Male 2:1** | **-125.666** | **-186.937** | **-64.396** | **0.002** |
| **Male 1:1 - Male 3:1** | **-235.260** | **-296.531** | **-173.990** | **6.65x10^-11^** |
| Female 2:1 - Male 2:1 | -40.278 | -121.926 | 41.369 | 0.975 |
| Female 2:1 - Female 3:1 | -90.143 | -156.995 | -23.292 | 0.127 |
| Male 2:1 - Male 3:1 | -109.594 | -170.864 | -48.324 | 0.01 |
| Female 3:1 - Male 3:1 | -59.729 | -141.377 | 21.918 | 0.82 |
|  | **CBD Peak** |  |  |  |
| Female 0:1 - Male 0:1 | -0.094 | -45.980 | 45.791 | 1 |
| Female 0:1 - Female 1:1 | -47.211 | -86.202 | -8.220 | 0.232 |
| **Female 0:1 - Female 2:1** | **-98.808** | **-137.799** | **-59.817** | **3.07x10^-5^** |
| **Female 0:1 - Female 3:1** | **-141.682** | **-180.673** | **-102.691** | **6.12x10^-10^** |
| **Male 0:1 - Male 1:1** | **-73.581** | **-109.317** | **-37.845** | **0.002** |
| **Male 0:1 - Male 2:1** | **-118.974** | **-154.709** | **-83.238** | **1.49 x10^-8^** |
| **Male 0:1 - Male 3:1** | **-164.015** | **-199.751** | **-128.279** | **6.98x10^-14^** |
| Female 1:1 - Male 1:1 | -26.464 | -72.350 | 19.421 | 0.941 |
| Female 1:1 - Female 2:1 | -51.597 | -90.588 | -12.606 | 0.143 |
| **Female 1:1 - Female 3:1** | **-94.471** | **-133.461** | **-55.480** | **8.05x10^-5^** |
| Male 1:1 - Male 2:1 | -45.393 | -81.128 | -9.657 | 0.181 |
| **Male 1:1 - Male 3:1** | **-90.434** | **-126.169** | **-54.698** | **3.16x10^-5^** |
| Female 2:1 - Male 2:1 | -20.260 | -66.145 | 25.625 | 0.987 |
| Female 2:1 - Female 3:1 | -42.874 | -81.865 | -3.883 | 0.35 |
| Male 2:1 - Male 3:1 | -45.041 | -80.777 | -9.305 | 0.189 |
| Female 3:1 - Male 3:1 | -22.428 | -68.313 | 23.458 | 0.976 |
|  | **OH-THC AUC** |  |  |  |
| Female 0:1 - Male 0:1 | -3.732 | -14.858 | 7.393 | 0.997 |
| Female 0:1 - Female 1:1 | -0.227 | -3.390 | 2.935 | 1.000 |
| Female 0:1 - Female 2:1 | -1.263 | -4.426 | 1.900 | 0.993 |
| Female 0:1 - Female 3:1 | -1.707 | -4.869 | 1.456 | 0.959 |
| Male 0:1 - Male 1:1 | -2.254 | -5.153 | 0.645 | 0.769 |
| Male 0:1 - Male 2:1 | -0.669 | -3.568 | 2.230 | 1.000 |
| Male 0:1 - Male 3:1 | -1.685 | -4.583 | 1.214 | 0.939 |
| Female 1:1 - Male 1:1 | -5.759 | -16.885 | 5.366 | 0.965 |
| Female 1:1 - Female 2:1 | -1.036 | -4.198 | 2.127 | 0.998 |
| Female 1:1 - Female 3:1 | -1.479 | -4.642 | 1.683 | 0.981 |
| Male 1:1 - Male 2:1 | 1.585 | -1.314 | 4.483 | 0.956 |
| Male 1:1 - Male 3:1 | 0.569 | -2.329 | 3.468 | 1.000 |
| Female 2:1 - Male 2:1 | -3.139 | -14.264 | 7.987 | 0.999 |
| Female 2:1 - Female 3:1 | -0.444 | -3.606 | 2.719 | 1.000 |
| Male 2:1 - Male 3:1 | -1.015 | -3.914 | 1.883 | 0.997 |
| Female 3:1 - Male 3:1 | -3.710 | -14.836 | 7.415 | 0.997 |
|  | **OH-THC Peak** |  |  |  |
| Female 0:1 - Male 0:1 | -0.893 | -4.132 | 2.346 | 0.999 |
| Female 0:1 - Female 1:1 | -0.163 | -1.208 | 0.883 | 1.000 |
| Female 0:1 - Female 2:1 | -0.659 | -1.705 | 0.386 | 0.908 |
| Female 0:1 - Female 3:1 | -0.332 | -1.377 | 0.714 | 0.998 |
| Male 0:1 - Male 1:1 | -0.871 | -1.830 | 0.087 | 0.600 |
| Male 0:1 - Male 2:1 | -0.264 | -1.223 | 0.694 | 0.999 |
| Male 0:1 - Male 3:1 | -0.273 | -1.232 | 0.685 | 0.999 |
| Female 1:1 - Male 1:1 | -1.601 | -4.840 | 1.638 | 0.973 |
| Female 1:1 - Female 2:1 | -0.497 | -1.542 | 0.549 | 0.980 |
| Female 1:1 - Female 3:1 | -0.169 | -1.214 | 0.877 | 1.000 |
| Male 1:1 - Male 2:1 | 0.607 | -0.351 | 1.565 | 0.906 |
| Male 1:1 - Male 3:1 | 0.598 | -0.360 | 1.556 | 0.913 |
| Female 2:1 - Male 2:1 | -0.498 | -3.737 | 2.741 | 1.000 |
| Female 2:1 - Female 3:1 | 0.328 | -0.718 | 1.373 | 0.998 |
| Male 2:1 - Male 3:1 | -0.009 | -0.967 | 0.949 | 1.000 |
| Female 3:1 - Male 3:1 | -0.834 | -4.073 | 2.405 | 1.000 |
|  | **COOH-THC AUC** | |  |  |
| Female 0:1 - Male 0:1 | -9.125 | -52.446 | 34.197 | 1.000 |
| Female 0:1 - Female 1:1 | 2.328 | -15.363 | 20.019 | 1.000 |
| Female 0:1 - Female 2:1 | -12.926 | -30.616 | 4.765 | 0.821 |
| Female 0:1 - Female 3:1 | -4.657 | -22.348 | 13.034 | 0.999 |
| Male 0:1 - Male 1:1 | -12.835 | -29.049 | 3.379 | 0.753 |
| Male 0:1 - Male 2:1 | -12.455 | -28.669 | 3.759 | 0.780 |
| Male 0:1 - Male 3:1 | -14.946 | -31.160 | 1.268 | 0.583 |
| Female 1:1 - Male 1:1 | -24.288 | -67.609 | 19.034 | 0.947 |
| Female 1:1 - Female 2:1 | -15.253 | -32.944 | 2.437 | 0.663 |
| Female 1:1 - Female 3:1 | -6.985 | -24.675 | 10.706 | 0.993 |
| Male 1:1 - Male 2:1 | 0.380 | -15.834 | 16.594 | 1.000 |
| Male 1:1 - Male 3:1 | -2.111 | -18.325 | 14.103 | 1.000 |
| Female 2:1 - Male 2:1 | -8.654 | -51.976 | 34.667 | 1.000 |
| Female 2:1 - Female 3:1 | 8.269 | -9.422 | 25.959 | 0.981 |
| Male 2:1 - Male 3:1 | -2.491 | -18.705 | 13.723 | 1.000 |
| Female 3:1 - Male 3:1 | -19.414 | -62.736 | 23.907 | 0.985 |
|  | **COOH-THC Peak** | |  |  |
| Female 0:1 - Male 0:1 | -0.892 | -12.395 | 10.610 | 1.000 |
| Female 0:1 - Female 1:1 | 0.571 | -6.119 | 7.261 | 1.000 |
| Female 0:1 - Female 2:1 | -3.554 | -10.243 | 3.136 | 0.962 |
| Female 0:1 - Female 3:1 | -2.189 | -8.874 | 4.497 | 0.998 |
| Male 0:1 - Male 1:1 | -6.879 | -13.005 | -0.753 | 0.323 |
| Male 0:1 - Male 2:1 | -2.588 | -8.715 | 3.540 | 0.990 |
| Male 0:1 - Male 3:1 | -4.302 | -10.467 | 1.863 | 0.853 |
| Female 1:1 - Male 1:1 | -8.342 | -19.842 | 3.157 | 0.825 |
| Female 1:1 - Female 2:1 | -4.125 | -10.809 | 2.559 | 0.917 |
| Female 1:1 - Female 3:1 | -2.760 | -9.457 | 3.937 | 0.991 |
| Male 1:1 - Male 2:1 | 4.291 | -1.836 | 10.418 | 0.851 |
| Male 1:1 - Male 3:1 | 2.577 | -3.589 | 8.742 | 0.990 |
| Female 2:1 - Male 2:1 | 0.074 | -11.426 | 11.574 | 1.000 |
| Female 2:1 - Female 3:1 | 1.365 | -5.332 | 8.062 | 1.000 |
| Male 2:1 - Male 3:1 | -1.714 | -7.894 | 4.465 | 0.999 |
| Female 3:1 - Male 3:1 | -3.006 | -14.510 | 8.498 | 0.999 |
|  | **7-OH-CBD AUC** | |  |  |
| Female 0:1 - Male 0:1 | -0.105 | -4.167 | 3.957 | 1 |
| Female 0:1 - Female 1:1 | -2.183 | -6.096 | 1.731 | 0.951 |
| Female 0:1 - Female 2:1 | -4.720 | -8.634 | -0.807 | 0.236 |
| **Female 0:1 - Female 3:1** | **-7.508** | **-11.421** | **-3.594** | **0.004** |
| Male 0:1 - Male 1:1 | -1.948 | -5.535 | 1.638 | 0.957 |
| Male 0:1 - Male 2:1 | -4.638 | -8.225 | -1.052 | 0.164 |
| **Male 0:1 - Male 3:1** | **-10.066** | **-13.653** | **-6.479** | **2.57x10^-6^** |
| Female 1:1 - Male 1:1 | 0.130 | -3.932 | 4.192 | 1 |
| Female 1:1 - Female 2:1 | -2.537 | -6.451 | 1.376 | 0.895 |
| Female 1:1 - Female 3:1 | -5.325 | -9.239 | -1.412 | 0.12 |
| Male 1:1 - Male 2:1 | -2.690 | -6.277 | 0.897 | 0.801 |
| **Male 1:1 - Male 3:1** | **-8.117** | **-11.704** | **-4.531** | **3.05x10^-4^** |
| Female 2:1 - Male 2:1 | -0.023 | -4.085 | 4.039 | 1 |
| Female 2:1 - Female 3:1 | -2.788 | -6.701 | 1.126 | 0.839 |
| Male 2:1 - Male 3:1 | -5.427 | -9.014 | -1.841 | 0.055 |
| Female 3:1 - Male 3:1 | -2.663 | -6.725 | 1.399 | 0.89 |
|  | **7-OH-CBD Peak** | |  |  |
| Female 0:1 - Male 0:1 | 0.000 | -1.827 | 1.827 | 1 |
| Female 0:1 - Female 1:1 | -0.610 | -2.445 | 1.226 | 0.998 |
| Female 0:1 - Female 2:1 | -0.846 | -2.681 | 0.990 | 0.983 |
| Female 0:1 - Female 3:1 | -2.436 | -4.272 | -0.601 | 0.14 |
| Male 0:1 - Male 1:1 | -0.371 | -2.054 | 1.311 | 1 |
| Male 0:1 - Male 2:1 | -0.967 | -2.650 | 0.715 | 0.942 |
| Male 0:1 - Male 3:1 | -2.895 | -4.577 | -1.213 | 0.016 |
| Female 1:1 - Male 1:1 | 0.238 | -1.589 | 2.065 | 1 |
| Female 1:1 - Female 2:1 | -0.236 | -2.072 | 1.600 | 1 |
| Female 1:1 - Female 3:1 | -1.827 | -3.663 | 0.009 | 0.483 |
| Male 1:1 - Male 2:1 | -0.596 | -2.278 | 1.087 | 0.996 |
| Male 1:1 - Male 3:1 | -2.524 | -4.206 | -0.841 | 0.059 |
| Female 2:1 - Male 2:1 | -0.121 | -1.949 | 1.706 | 1 |
| Female 2:1 - Female 3:1 | -1.591 | -3.426 | 0.245 | 0.658 |
| Male 2:1 - Male 3:1 | -1.928 | -3.610 | -0.245 | 0.297 |
| Female 3:1 - Male 3:1 | -0.459 | -2.286 | 1.369 | 1 |
|  | **HVLT Immediate** | |  |  |
| Female 0:1 - Male 0:1 | -1.364 | -4.158 | 1.430 | 0.976 |
| Female 0:1 - Female 1:1 | 0.333 | -1.574 | 2.241 | 1.000 |
| Female 0:1 - Female 2:1 | 0.714 | -1.193 | 2.622 | 0.995 |
| Female 0:1 - Female 3:1 | 0.048 | -1.860 | 1.955 | 1.000 |
| Male 0:1 - Male 1:1 | 0.720 | -1.028 | 2.468 | 0.991 |
| Male 0:1 - Male 2:1 | 1.480 | -0.268 | 3.228 | 0.684 |
| Male 0:1 - Male 3:1 | -0.400 | -2.148 | 1.348 | 1.000 |
| Female 1:1 - Male 1:1 | -0.977 | -3.771 | 1.817 | 0.997 |
| Female 1:1 - Female 2:1 | 0.381 | -1.527 | 2.289 | 1.000 |
| Female 1:1 - Female 3:1 | -0.286 | -2.193 | 1.622 | 1.000 |
| Male 1:1 - Male 2:1 | 0.760 | -0.988 | 2.508 | 0.988 |
| Male 1:1 - Male 3:1 | -1.120 | -2.868 | 0.628 | 0.901 |
| Female 2:1 - Male 2:1 | -0.598 | -3.392 | 2.196 | 1.000 |
| Female 2:1 - Female 3:1 | -0.667 | -2.574 | 1.241 | 0.997 |
| Male 2:1 - Male 3:1 | -1.880 | -3.628 | -0.132 | 0.379 |
| Female 3:1 - Male 3:1 | -1.811 | -4.605 | 0.982 | 0.894 |
|  | **HVLT Delayed** | |  |  |
| Female 0:1 - Male 0:1 | -0.760 | -2.128 | 0.608 | 0.951 |
| Female 0:1 - Female 1:1 | 0.286 | -0.715 | 1.286 | 0.999 |
| Female 0:1 - Female 2:1 | -0.048 | -1.048 | 0.953 | 1.000 |
| Female 0:1 - Female 3:1 | 0.095 | -0.905 | 1.096 | 1.000 |
| Male 0:1 - Male 1:1 | 0.040 | -0.877 | 0.957 | 1.000 |
| Male 0:1 - Male 2:1 | 0.640 | -0.277 | 1.557 | 0.853 |
| Male 0:1 - Male 3:1 | -0.040 | -0.957 | 0.877 | 1.000 |
| Female 1:1 - Male 1:1 | -1.006 | -2.374 | 0.363 | 0.816 |
| Female 1:1 - Female 2:1 | -0.333 | -1.334 | 0.667 | 0.998 |
| Female 1:1 - Female 3:1 | -0.190 | -1.191 | 0.810 | 1.000 |
| Male 1:1 - Male 2:1 | 0.600 | -0.317 | 1.517 | 0.891 |
| Male 1:1 - Male 3:1 | -0.080 | -0.997 | 0.837 | 1.000 |
| Female 2:1 - Male 2:1 | -0.072 | -1.441 | 1.296 | 1.000 |
| Female 2:1 - Female 3:1 | 0.143 | -0.858 | 1.144 | 1.000 |
| Male 2:1 - Male 3:1 | -0.680 | -1.597 | 0.237 | 0.810 |
| Female 3:1 - Male 3:1 | -0.895 | -2.264 | 0.473 | 0.890 |
|  | **HVLT Retention** | |  |  |
| Female 0:1 - Male 0:1 | -4.816 | -13.828 | 4.197 | 0.961 |
| Female 0:1 - Female 1:1 | 1.471 | -6.795 | 9.738 | 1.000 |
| Female 0:1 - Female 2:1 | -0.510 | -8.776 | 7.757 | 1.000 |
| Female 0:1 - Female 3:1 | 2.076 | -6.190 | 10.343 | 1.000 |
| Male 0:1 - Male 1:1 | -1.056 | -8.632 | 6.520 | 1.000 |
| Male 0:1 - Male 2:1 | 4.932 | -2.644 | 12.508 | 0.893 |
| Male 0:1 - Male 3:1 | 2.640 | -4.936 | 10.216 | 0.997 |
| Female 1:1 - Male 1:1 | -7.343 | -16.356 | 1.670 | 0.725 |
| Female 1:1 - Female 2:1 | -1.981 | -10.247 | 6.286 | 1.000 |
| Female 1:1 - Female 3:1 | 0.605 | -7.662 | 8.871 | 1.000 |
| Male 1:1 - Male 2:1 | 5.988 | -1.588 | 13.564 | 0.754 |
| Male 1:1 - Male 3:1 | 3.696 | -3.880 | 11.272 | 0.976 |
| Female 2:1 - Male 2:1 | 0.626 | -8.387 | 9.639 | 1.000 |
| Female 2:1 - Female 3:1 | 2.586 | -5.681 | 10.852 | 0.998 |
| Male 2:1 - Male 3:1 | -2.292 | -9.868 | 5.284 | 0.999 |
| Female 3:1 - Male 3:1 | -4.252 | -13.264 | 4.761 | 0.980 |
|  | **HVLT Delayed Repetitions** | |  |  |
| Female 0:1 - Male 0:1 | 0.476 | 0.176 | 0.776 | 0.035 |
| Female 0:1 - Female 1:1 | 0.381 | 0.081 | 0.680 | 0.180 |
| Female 0:1 - Female 2:1 | 0.238 | -0.061 | 0.538 | 0.749 |
| Female 0:1 - Female 3:1 | 0.286 | -0.014 | 0.585 | 0.539 |
| Male 0:1 - Male 1:1 | -0.080 | -0.355 | 0.195 | 0.999 |
| Male 0:1 - Male 2:1 | -0.160 | -0.435 | 0.115 | 0.938 |
| Male 0:1 - Male 3:1 | -0.160 | -0.435 | 0.115 | 0.938 |
| Female 1:1 - Male 1:1 | 0.015 | -0.285 | 0.316 | 1.000 |
| Female 1:1 - Female 2:1 | -0.143 | -0.442 | 0.157 | 0.979 |
| Female 1:1 - Female 3:1 | -0.095 | -0.395 | 0.204 | 0.998 |
| Male 1:1 - Male 2:1 | -0.080 | -0.355 | 0.195 | 0.999 |
| Male 1:1 - Male 3:1 | -0.080 | -0.355 | 0.195 | 0.999 |
| Female 2:1 - Male 2:1 | 0.078 | -0.222 | 0.378 | 1.000 |
| Female 2:1 - Female 3:1 | 0.048 | -0.252 | 0.347 | 1.000 |
| Male 2:1 - Male 3:1 | 0.000 | -0.275 | 0.275 | 1.000 |
| Female 3:1 - Male 3:1 | 0.030 | -0.270 | 0.331 | 1.000 |
|  | **HVLT Delayed Intrusions** | |  |  |
| Female 0:1 - Male 0:1 | 0.017 | -0.572 | 0.606 | 1.000 |
| Female 0:1 - Female 1:1 | 0.048 | -0.495 | 0.591 | 1.000 |
| Female 0:1 - Female 2:1 | 0.048 | -0.495 | 0.591 | 1.000 |
| Female 0:1 - Female 3:1 | 0.095 | -0.448 | 0.638 | 1.000 |
| Male 0:1 - Male 1:1 | 0.240 | -0.258 | 0.738 | 0.978 |
| Male 0:1 - Male 2:1 | 0.360 | -0.138 | 0.858 | 0.828 |
| Male 0:1 - Male 3:1 | 0.000 | -0.498 | 0.498 | 1.000 |
| Female 1:1 - Male 1:1 | 0.210 | -0.380 | 0.799 | 0.996 |
| Female 1:1 - Female 2:1 | 0.000 | -0.543 | 0.543 | 1.000 |
| Female 1:1 - Female 3:1 | 0.048 | -0.495 | 0.591 | 1.000 |
| Male 1:1 - Male 2:1 | 0.120 | -0.378 | 0.618 | 1.000 |
| Male 1:1 - Male 3:1 | -0.240 | -0.738 | 0.258 | 0.978 |
| Female 2:1 - Male 2:1 | 0.330 | -0.260 | 0.919 | 0.950 |
| Female 2:1 - Female 3:1 | 0.048 | -0.495 | 0.591 | 1.000 |
| Male 2:1 - Male 3:1 | -0.360 | -0.858 | 0.138 | 0.828 |
| Female 3:1 - Male 3:1 | -0.078 | -0.667 | 0.511 | 1.000 |
|  | **HVLT Immediate Repetitions** | |  |  |
| Female 0:1 - Male 0:1 | 0.448 | -0.739 | 1.634 | 0.995 |
| Female 0:1 - Female 1:1 | 0.905 | -0.054 | 1.864 | 0.553 |
| Female 0:1 - Female 2:1 | 0.810 | -0.150 | 1.769 | 0.687 |
| Female 0:1 - Female 3:1 | 0.762 | -0.197 | 1.721 | 0.749 |
| Male 0:1 - Male 1:1 | 0.280 | -0.599 | 1.159 | 0.998 |
| Male 0:1 - Male 2:1 | 0.560 | -0.319 | 1.439 | 0.904 |
| Male 0:1 - Male 3:1 | -0.280 | -1.159 | 0.599 | 0.998 |
| Female 1:1 - Male 1:1 | -0.177 | -1.363 | 1.009 | 1.000 |
| Female 1:1 - Female 2:1 | -0.095 | -1.054 | 0.864 | 1.000 |
| Female 1:1 - Female 3:1 | -0.143 | -1.102 | 0.816 | 1.000 |
| Male 1:1 - Male 2:1 | 0.280 | -0.599 | 1.159 | 0.998 |
| Male 1:1 - Male 3:1 | -0.560 | -1.439 | 0.319 | 0.904 |
| Female 2:1 - Male 2:1 | 0.198 | -0.988 | 1.384 | 1.000 |
| Female 2:1 - Female 3:1 | -0.048 | -1.007 | 0.911 | 1.000 |
| Male 2:1 - Male 3:1 | -0.840 | -1.719 | 0.039 | 0.537 |
| Female 3:1 - Male 3:1 | -0.594 | -1.780 | 0.592 | 0.972 |
|  | **HVLT Immediate Intrusions** | |  |  |
| Female 0:1 - Male 0:1 | 0.800 | -0.325 | 1.925 | 0.841 |
| Female 0:1 - Female 1:1 | -0.095 | -1.139 | 0.949 | 1.000 |
| Female 0:1 - Female 2:1 | 0.190 | -0.853 | 1.234 | 1.000 |
| Female 0:1 - Female 3:1 | 0.048 | -0.996 | 1.092 | 1.000 |
| Male 0:1 - Male 1:1 | 0.080 | -0.877 | 1.037 | 1.000 |
| Male 0:1 - Male 2:1 | 0.120 | -0.837 | 1.077 | 1.000 |
| Male 0:1 - Male 3:1 | -0.440 | -1.397 | 0.517 | 0.983 |
| Female 1:1 - Male 1:1 | 0.975 | -0.149 | 2.100 | 0.657 |
| Female 1:1 - Female 2:1 | 0.286 | -0.758 | 1.330 | 0.999 |
| Female 1:1 - Female 3:1 | 0.143 | -0.901 | 1.187 | 1.000 |
| Male 1:1 - Male 2:1 | 0.040 | -0.917 | 0.997 | 1.000 |
| Male 1:1 - Male 3:1 | -0.520 | -1.477 | 0.437 | 0.957 |
| Female 2:1 - Male 2:1 | 0.730 | -0.395 | 1.854 | 0.895 |
| Female 2:1 - Female 3:1 | -0.143 | -1.187 | 0.901 | 1.000 |
| Male 2:1 - Male 3:1 | -0.560 | -1.517 | 0.397 | 0.937 |
| Female 3:1 - Male 3:1 | 0.312 | -0.812 | 1.437 | 0.999 |
|  | **Digit Span Forward** | |  |  |
| Female 0:1 - Male 0:1 | -0.387 | -1.118 | 0.345 | 0.963 |
| Female 0:1 - Female 1:1 | 0.048 | -0.520 | 0.615 | 1.000 |
| Female 0:1 - Female 2:1 | 0.190 | -0.377 | 0.758 | 0.997 |
| Female 0:1 - Female 3:1 | 0.095 | -0.472 | 0.663 | 1.000 |
| Male 0:1 - Male 1:1 | 0.400 | -0.120 | 0.920 | 0.779 |
| Male 0:1 - Male 2:1 | 0.280 | -0.240 | 0.800 | 0.959 |
| Male 0:1 - Male 3:1 | 0.000 | -0.520 | 0.520 | 1.000 |
| Female 1:1 - Male 1:1 | -0.034 | -0.766 | 0.697 | 1.000 |
| Female 1:1 - Female 2:1 | 0.143 | -0.425 | 0.710 | 1.000 |
| Female 1:1 - Female 3:1 | 0.048 | -0.520 | 0.615 | 1.000 |
| Male 1:1 - Male 2:1 | -0.120 | -0.640 | 0.400 | 1.000 |
| Male 1:1 - Male 3:1 | -0.400 | -0.920 | 0.120 | 0.779 |
| Female 2:1 - Male 2:1 | -0.297 | -1.029 | 0.434 | 0.992 |
| Female 2:1 - Female 3:1 | -0.095 | -0.663 | 0.472 | 1.000 |
| Male 2:1 - Male 3:1 | -0.280 | -0.800 | 0.240 | 0.959 |
| Female 3:1 - Male 3:1 | -0.482 | -1.213 | 0.250 | 0.887 |
|  | **Digit Span Reverse** | |  |  |
| Female 0:1 - Male 0:1 | -0.438 | -1.051 | 0.175 | 0.837 |
| Female 0:1 - Female 1:1 | 0.048 | -0.480 | 0.575 | 1.000 |
| Female 0:1 - Female 2:1 | -0.095 | -0.623 | 0.432 | 1.000 |
| Female 0:1 - Female 3:1 | 0.000 | -0.527 | 0.527 | 1.000 |
| Male 0:1 - Male 1:1 | 0.080 | -0.403 | 0.563 | 1.000 |
| Male 0:1 - Male 2:1 | 0.240 | -0.243 | 0.723 | 0.974 |
| Male 0:1 - Male 3:1 | 0.120 | -0.363 | 0.603 | 1.000 |
| Female 1:1 - Male 1:1 | -0.406 | -1.018 | 0.207 | 0.884 |
| Female 1:1 - Female 2:1 | -0.143 | -0.670 | 0.385 | 0.999 |
| Female 1:1 - Female 3:1 | -0.048 | -0.575 | 0.480 | 1.000 |
| Male 1:1 - Male 2:1 | 0.160 | -0.323 | 0.643 | 0.998 |
| Male 1:1 - Male 3:1 | 0.040 | -0.443 | 0.523 | 1.000 |
| Female 2:1 - Male 2:1 | -0.103 | -0.716 | 0.510 | 1.000 |
| Female 2:1 - Female 3:1 | 0.095 | -0.432 | 0.623 | 1.000 |
| Male 2:1 - Male 3:1 | -0.120 | -0.603 | 0.363 | 1.000 |
| Female 3:1 - Male 3:1 | -0.318 | -0.931 | 0.295 | 0.966 |
|  | **Spatial N-Back Zero Back** | |  |  |
| Female 0:1 - Male 0:1 | -0.026 | -0.064 | 0.013 | 0.883 |
| Female 0:1 - Female 1:1 | -0.008 | -0.041 | 0.025 | 1.000 |
| Female 0:1 - Female 2:1 | -0.018 | -0.051 | 0.015 | 0.959 |
| Female 0:1 - Female 3:1 | -0.019 | -0.052 | 0.014 | 0.942 |
| Male 0:1 - Male 1:1 | 0.007 | -0.023 | 0.037 | 1.000 |
| Male 0:1 - Male 2:1 | -0.014 | -0.044 | 0.016 | 0.983 |
| Male 0:1 - Male 3:1 | -0.018 | -0.048 | 0.012 | 0.933 |
| Female 1:1 - Male 1:1 | -0.010 | -0.049 | 0.028 | 0.999 |
| Female 1:1 - Female 2:1 | -0.010 | -0.043 | 0.024 | 0.999 |
| Female 1:1 - Female 3:1 | -0.011 | -0.044 | 0.022 | 0.998 |
| Male 1:1 - Male 2:1 | -0.021 | -0.051 | 0.009 | 0.860 |
| Male 1:1 - Male 3:1 | -0.025 | -0.055 | 0.005 | 0.715 |
| Female 2:1 - Male 2:1 | -0.022 | -0.061 | 0.017 | 0.948 |
| Female 2:1 - Female 3:1 | -0.001 | -0.034 | 0.032 | 1.000 |
| Male 2:1 - Male 3:1 | -0.004 | -0.034 | 0.026 | 1.000 |
| Female 3:1 - Male 3:1 | -0.025 | -0.063 | 0.014 | 0.904 |
|  | **Spatial N-Back One Back** | |  |  |
| Female 0:1 - Male 0:1 | -0.112 | -0.182 | -0.042 | 0.033 |
| Female 0:1 - Female 1:1 | -0.017 | -0.075 | 0.042 | 0.999 |
| Female 0:1 - Female 2:1 | -0.065 | -0.124 | -0.007 | 0.332 |
| Female 0:1 - Female 3:1 | -0.019 | -0.078 | 0.040 | 0.998 |
| Male 0:1 - Male 1:1 | 0.057 | 0.003 | 0.111 | 0.399 |
| Male 0:1 - Male 2:1 | 0.031 | -0.023 | 0.085 | 0.942 |
| Male 0:1 - Male 3:1 | 0.035 | -0.019 | 0.089 | 0.894 |
| Female 1:1 - Male 1:1 | -0.039 | -0.109 | 0.031 | 0.953 |
| Female 1:1 - Female 2:1 | -0.049 | -0.108 | 0.010 | 0.704 |
| Female 1:1 - Female 3:1 | -0.002 | -0.061 | 0.056 | 1.000 |
| Male 1:1 - Male 2:1 | -0.026 | -0.080 | 0.028 | 0.977 |
| Male 1:1 - Male 3:1 | -0.022 | -0.076 | 0.032 | 0.992 |
| Female 2:1 - Male 2:1 | -0.016 | -0.086 | 0.054 | 1.000 |
| Female 2:1 - Female 3:1 | 0.046 | -0.012 | 0.105 | 0.754 |
| Male 2:1 - Male 3:1 | 0.004 | -0.050 | 0.058 | 1.000 |
| Female 3:1 - Male 3:1 | -0.058 | -0.128 | 0.012 | 0.703 |
|  | **Spatial N-Back Two Back** | |  |  |
| Female 0:1 - Male 0:1 | -0.092 | -0.192 | 0.008 | 0.581 |
| Female 0:1 - Female 1:1 | -0.008 | -0.068 | 0.051 | 1.000 |
| Female 0:1 - Female 2:1 | 0.042 | -0.018 | 0.101 | 0.850 |
| Female 0:1 - Female 3:1 | -0.019 | -0.078 | 0.040 | 0.998 |
| Male 0:1 - Male 1:1 | -0.017 | -0.071 | 0.037 | 0.998 |
| Male 0:1 - Male 2:1 | -0.029 | -0.083 | 0.025 | 0.961 |
| Male 0:1 - Male 3:1 | -0.019 | -0.073 | 0.035 | 0.997 |
| Female 1:1 - Male 1:1 | -0.101 | -0.201 | -0.001 | 0.465 |
| Female 1:1 - Female 2:1 | 0.050 | -0.009 | 0.109 | 0.690 |
| Female 1:1 - Female 3:1 | -0.011 | -0.070 | 0.049 | 1.000 |
| Male 1:1 - Male 2:1 | -0.012 | -0.066 | 0.042 | 1.000 |
| Male 1:1 - Male 3:1 | -0.002 | -0.056 | 0.052 | 1.000 |
| Female 2:1 - Male 2:1 | -0.163 | -0.263 | -0.063 | 0.032 |
| Female 2:1 - Female 3:1 | -0.061 | -0.120 | -0.001 | 0.447 |
| Male 2:1 - Male 3:1 | 0.010 | -0.044 | 0.064 | 1.000 |
| Female 3:1 - Male 3:1 | -0.092 | -0.192 | 0.008 | 0.581 |
|  | **PANSS** | |  |  |
| Female 0:1 - Male 0:1 | 0.019 | -1.294 | 1.332 | 1.000 |
| Female 0:1 - Female 1:1 | -1.069 | -2.150 | 0.011 | 0.490 |
| Female 0:1 - Female 2:1 | -0.714 | -1.779 | 0.350 | 0.877 |
| Female 0:1 - Female 3:1 | -0.374 | -1.454 | 0.707 | 0.997 |
| Male 0:1 - Male 1:1 | -0.320 | -1.295 | 0.655 | 0.998 |
| Male 0:1 - Male 2:1 | 0.440 | -0.535 | 1.415 | 0.985 |
| Male 0:1 - Male 3:1 | 0.120 | -0.855 | 1.095 | 1.000 |
| Female 1:1 - Male 1:1 | 0.768 | -0.558 | 2.095 | 0.940 |
| Female 1:1 - Female 2:1 | 0.355 | -0.726 | 1.436 | 0.998 |
| Female 1:1 - Female 3:1 | 0.695 | -0.402 | 1.792 | 0.906 |
| Male 1:1 - Male 2:1 | 0.760 | -0.215 | 1.735 | 0.768 |
| Male 1:1 - Male 3:1 | 0.440 | -0.535 | 1.415 | 0.985 |
| Female 2:1 - Male 2:1 | 1.173 | -0.140 | 2.487 | 0.622 |
| Female 2:1 - Female 3:1 | 0.340 | -0.740 | 1.421 | 0.998 |
| Male 2:1 - Male 3:1 | -0.320 | -1.295 | 0.655 | 0.998 |
| Female 3:1 - Male 3:1 | 0.513 | -0.814 | 1.840 | 0.994 |
|  | **SSPS** | |  |  |
| Female 0:1 - Male 0:1 | -0.272 | -0.753 | 0.208 | 0.946 |
| Female 0:1 - Female 1:1 | -0.619 | -1.113 | -0.125 | 0.194 |
| Female 0:1 - Female 2:1 | -0.381 | -0.875 | 0.113 | 0.776 |
| Female 0:1 - Female 3:1 | -0.095 | -0.589 | 0.399 | 1.000 |
| Male 0:1 - Male 1:1 | 0.000 | -0.453 | 0.453 | 1.000 |
| Male 0:1 - Male 2:1 | -0.120 | -0.573 | 0.333 | 0.999 |
| Male 0:1 - Male 3:1 | 0.240 | -0.213 | 0.693 | 0.962 |
| Female 1:1 - Male 1:1 | 0.347 | -0.134 | 0.827 | 0.830 |
| Female 1:1 - Female 2:1 | 0.238 | -0.256 | 0.732 | 0.978 |
| Female 1:1 - Female 3:1 | 0.524 | 0.030 | 1.018 | 0.397 |
| Male 1:1 - Male 2:1 | -0.120 | -0.573 | 0.333 | 0.999 |
| Male 1:1 - Male 3:1 | 0.240 | -0.213 | 0.693 | 0.962 |
| Female 2:1 - Male 2:1 | -0.011 | -0.492 | 0.469 | 1.000 |
| Female 2:1 - Female 3:1 | 0.286 | -0.208 | 0.779 | 0.940 |
| Male 2:1 - Male 3:1 | 0.360 | -0.093 | 0.813 | 0.748 |
| Female 3:1 - Male 3:1 | 0.063 | -0.417 | 0.543 | 1.000 |
|  | **CAPE** | |  |  |
| Female 0:1 - Male 0:1 | 0.328 | -2.578 | 3.235 | 1.000 |
| Female 0:1 - Female 1:1 | -1.236 | -3.269 | 0.797 | 0.923 |
| Female 0:1 - Female 2:1 | -0.085 | -2.118 | 1.948 | 1.000 |
| Female 0:1 - Female 3:1 | -2.762 | -4.763 | -0.761 | 0.109 |
| Male 0:1 - Male 1:1 | 1.515 | -0.383 | 3.413 | 0.745 |
| Male 0:1 - Male 2:1 | -0.494 | -2.354 | 1.366 | 0.999 |
| Male 0:1 - Male 3:1 | 1.146 | -0.714 | 3.006 | 0.918 |
| Female 1:1 - Male 1:1 | 3.079 | 0.134 | 6.025 | 0.419 |
| Female 1:1 - Female 2:1 | 1.151 | -0.914 | 3.215 | 0.951 |
| Female 1:1 - Female 3:1 | -1.526 | -3.559 | 0.507 | 0.800 |
| Male 1:1 - Male 2:1 | -2.009 | -3.895 | -0.123 | 0.392 |
| Male 1:1 - Male 3:1 | -0.369 | -2.255 | 1.517 | 1.000 |
| Female 2:1 - Male 2:1 | -0.080 | -2.993 | 2.832 | 1.000 |
| Female 2:1 - Female 3:1 | -2.677 | -4.709 | -0.644 | 0.148 |
| Male 2:1 - Male 3:1 | 1.640 | -0.194 | 3.474 | 0.621 |
| Female 3:1 - Male 3:1 | 4.236 | 1.346 | 7.127 | 0.074 |
|  | **PSI** | |  |  |
| Female 0:1 - Male 0:1 | -3.849 | -12.589 | 4.892 | 0.987 |
| Female 0:1 - Female 1:1 | -4.409 | -12.518 | 3.700 | 0.956 |
| Female 0:1 - Female 2:1 | -6.597 | -14.544 | 1.349 | 0.705 |
| Female 0:1 - Female 3:1 | -4.152 | -11.932 | 3.628 | 0.960 |
| Male 0:1 - Male 1:1 | -0.223 | -7.838 | 7.393 | 1.000 |
| Male 0:1 - Male 2:1 | 1.564 | -5.925 | 9.053 | 1.000 |
| Male 0:1 - Male 3:1 | 4.847 | -2.374 | 12.068 | 0.875 |
| Female 1:1 - Male 1:1 | 0.337 | -8.194 | 8.869 | 1.000 |
| Female 1:1 - Female 2:1 | -2.189 | -9.890 | 5.513 | 0.999 |
| Female 1:1 - Female 3:1 | 0.257 | -7.280 | 7.794 | 1.000 |
| Male 1:1 - Male 2:1 | 1.786 | -5.698 | 9.271 | 1.000 |
| Male 1:1 - Male 3:1 | 5.069 | -2.093 | 12.232 | 0.842 |
| Female 2:1 - Male 2:1 | 4.312 | -3.717 | 12.341 | 0.959 |
| Female 2:1 - Female 3:1 | 2.446 | -4.697 | 9.588 | 0.997 |
| Male 2:1 - Male 3:1 | 3.283 | -3.739 | 10.305 | 0.981 |
| Female 3:1 - Male 3:1 | 5.150 | -2.357 | 12.657 | 0.863 |
|  | **VAS Drug effects pleasurable AUC** | |  |  |
| Female 0:1 - Male 0:1 | 1.087 | -3.011 | 5.185 | 0.999 |
| Female 0:1 - Female 1:1 | 2.205 | -0.897 | 5.307 | 0.841 |
| Female 0:1 - Female 2:1 | 3.728 | 0.626 | 6.830 | 0.240 |
| Female 0:1 - Female 3:1 | 2.296 | -0.806 | 5.398 | 0.811 |
| Male 0:1 - Male 1:1 | -0.014 | -2.789 | 2.760 | 1.000 |
| Male 0:1 - Male 2:1 | 1.288 | -1.486 | 4.063 | 0.982 |
| Male 0:1 - Male 3:1 | -0.226 | -3.001 | 2.548 | 1.000 |
| Female 1:1 - Male 1:1 | -1.133 | -5.231 | 2.965 | 0.999 |
| Female 1:1 - Female 2:1 | 1.523 | -1.579 | 4.625 | 0.975 |
| Female 1:1 - Female 3:1 | 0.091 | -3.011 | 3.193 | 1.000 |
| Male 1:1 - Male 2:1 | 1.303 | -1.472 | 4.077 | 0.981 |
| Male 1:1 - Male 3:1 | -0.212 | -2.986 | 2.562 | 1.000 |
| Female 2:1 - Male 2:1 | -1.353 | -5.451 | 2.745 | 0.998 |
| Female 2:1 - Female 3:1 | -1.432 | -4.534 | 1.670 | 0.983 |
| Male 2:1 - Male 3:1 | -1.515 | -4.289 | 1.260 | 0.956 |
| Female 3:1 - Male 3:1 | -1.436 | -5.534 | 2.662 | 0.997 |
|  | **VAS Drug effects pleasurable peak** | |  |  |
| Female 0:1 - Male 0:1 | 0.198 | -1.144 | 1.540 | 1.000 |
| Female 0:1 - Female 1:1 | 0.635 | -0.345 | 1.615 | 0.895 |
| Female 0:1 - Female 2:1 | 0.815 | -0.165 | 1.795 | 0.703 |
| Female 0:1 - Female 3:1 | 0.435 | -0.545 | 1.415 | 0.986 |
| Male 0:1 - Male 1:1 | 0.136 | -0.740 | 1.012 | 1.000 |
| Male 0:1 - Male 2:1 | 0.220 | -0.656 | 1.096 | 1.000 |
| Male 0:1 - Male 3:1 | 0.004 | -0.872 | 0.880 | 1.000 |
| Female 1:1 - Male 1:1 | -0.301 | -1.643 | 1.041 | 1.000 |
| Female 1:1 - Female 2:1 | 0.180 | -0.800 | 1.160 | 1.000 |
| Female 1:1 - Female 3:1 | -0.200 | -1.180 | 0.780 | 1.000 |
| Male 1:1 - Male 2:1 | 0.084 | -0.792 | 0.960 | 1.000 |
| Male 1:1 - Male 3:1 | -0.132 | -1.008 | 0.744 | 1.000 |
| Female 2:1 - Male 2:1 | -0.397 | -1.739 | 0.945 | 0.999 |
| Female 2:1 - Female 3:1 | -0.380 | -1.360 | 0.600 | 0.994 |
| Male 2:1 - Male 3:1 | -0.216 | -1.092 | 0.660 | 1.000 |
| Female 3:1 - Male 3:1 | -0.233 | -1.575 | 1.109 | 1.000 |
|  | **Dry mouth AUC** | |  |  |
| Female 0:1 - Male 0:1 | -0.209 | -4.750 | 4.331 | 1.000 |
| Female 0:1 - Female 1:1 | -1.836 | -5.556 | 1.883 | 0.974 |
| Female 0:1 - Female 2:1 | -2.105 | -5.824 | 1.615 | 0.947 |
| Female 0:1 - Female 3:1 | -5.338 | -9.057 | -1.618 | 0.083 |
| Male 0:1 - Male 1:1 | 0.453 | -2.873 | 3.780 | 1.000 |
| Male 0:1 - Male 2:1 | 1.613 | -1.713 | 4.940 | 0.977 |
| Male 0:1 - Male 3:1 | 1.249 | -2.078 | 4.576 | 0.995 |
| Female 1:1 - Male 1:1 | 2.080 | -2.460 | 6.621 | 0.983 |
| Female 1:1 - Female 2:1 | -0.268 | -3.987 | 3.451 | 1.000 |
| Female 1:1 - Female 3:1 | -3.501 | -7.220 | 0.218 | 0.556 |
| Male 1:1 - Male 2:1 | 1.160 | -2.167 | 4.486 | 0.997 |
| Male 1:1 - Male 3:1 | 0.796 | -2.531 | 4.122 | 1.000 |
| Female 2:1 - Male 2:1 | 3.508 | -1.032 | 8.049 | 0.775 |
| Female 2:1 - Female 3:1 | -3.233 | -6.952 | 0.486 | 0.654 |
| Male 2:1 - Male 3:1 | -0.364 | -3.691 | 2.962 | 1.000 |
| Female 3:1 - Male 3:1 | 6.377 | 1.837 | 10.918 | 0.098 |
|  | **Dry mouth peak** | |  |  |
| Female 0:1 - Male 0:1 | -0.499 | -2.139 | 1.141 | 0.999 |
| Female 0:1 - Female 1:1 | -0.295 | -1.638 | 1.048 | 1.000 |
| Female 0:1 - Female 2:1 | -0.885 | -2.228 | 0.458 | 0.887 |
| Female 0:1 - Female 3:1 | -1.275 | -2.618 | 0.068 | 0.545 |
| Male 0:1 - Male 1:1 | 0.164 | -1.037 | 1.365 | 1.000 |
| Male 0:1 - Male 2:1 | 1.520 | 0.319 | 2.721 | 0.185 |
| Male 0:1 - Male 3:1 | 0.968 | -0.233 | 2.169 | 0.735 |
| Female 1:1 - Male 1:1 | -0.040 | -1.680 | 1.600 | 1.000 |
| Female 1:1 - Female 2:1 | -0.590 | -1.933 | 0.753 | 0.987 |
| Female 1:1 - Female 3:1 | -0.980 | -2.323 | 0.363 | 0.822 |
| Male 1:1 - Male 2:1 | 1.356 | 0.155 | 2.557 | 0.316 |
| Male 1:1 - Male 3:1 | 0.804 | -0.397 | 2.005 | 0.878 |
| Female 2:1 - Male 2:1 | 1.906 | 0.266 | 3.546 | 0.281 |
| Female 2:1 - Female 3:1 | -0.390 | -1.733 | 0.953 | 0.999 |
| Male 2:1 - Male 3:1 | -0.552 | -1.753 | 0.649 | 0.983 |
| Female 3:1 - Male 3:1 | 1.744 | 0.104 | 3.384 | 0.395 |
|  | **Enhanced colour perception AUC** | |  |  |
| Female 0:1 - Male 0:1 | -1.964 | -7.162 | 3.233 | 0.995 |
| Female 0:1 - Female 1:1 | -0.271 | -3.401 | 2.859 | 1.000 |
| Female 0:1 - Female 2:1 | -1.552 | -4.682 | 1.578 | 0.974 |
| Female 0:1 - Female 3:1 | -3.194 | -6.324 | -0.064 | 0.449 |
| Male 0:1 - Male 1:1 | -0.894 | -3.693 | 1.906 | 0.998 |
| Male 0:1 - Male 2:1 | -0.055 | -2.855 | 2.744 | 1.000 |
| Male 0:1 - Male 3:1 | -1.312 | -4.111 | 1.488 | 0.981 |
| Female 1:1 - Male 1:1 | -2.587 | -7.785 | 2.611 | 0.973 |
| Female 1:1 - Female 2:1 | -1.281 | -4.411 | 1.849 | 0.991 |
| Female 1:1 - Female 3:1 | -2.923 | -6.053 | 0.207 | 0.566 |
| Male 1:1 - Male 2:1 | 0.838 | -1.961 | 3.638 | 0.999 |
| Male 1:1 - Male 3:1 | -0.418 | -3.217 | 2.381 | 1.000 |
| Female 2:1 - Male 2:1 | -0.468 | -5.665 | 4.730 | 1.000 |
| Female 2:1 - Female 3:1 | -1.642 | -4.772 | 1.488 | 0.964 |
| Male 2:1 - Male 3:1 | -1.256 | -4.056 | 1.543 | 0.985 |
| Female 3:1 - Male 3:1 | -0.082 | -5.280 | 5.115 | 1.000 |
|  | **Enhanced colour perception peak** | |  |  |
| Female 0:1 - Male 0:1 | -0.940 | -2.777 | 0.897 | 0.969 |
| Female 0:1 - Female 1:1 | -0.035 | -1.396 | 1.326 | 1.000 |
| Female 0:1 - Female 2:1 | -0.830 | -2.191 | 0.531 | 0.922 |
| Female 0:1 - Female 3:1 | -1.370 | -2.731 | -0.009 | 0.467 |
| Male 0:1 - Male 1:1 | -0.100 | -1.317 | 1.117 | 1.000 |
| Male 0:1 - Male 2:1 | -0.012 | -1.229 | 1.205 | 1.000 |
| Male 0:1 - Male 3:1 | -0.216 | -1.433 | 1.001 | 1.000 |
| Female 1:1 - Male 1:1 | -1.005 | -2.842 | 0.832 | 0.955 |
| Female 1:1 - Female 2:1 | -0.795 | -2.156 | 0.566 | 0.937 |
| Female 1:1 - Female 3:1 | -1.335 | -2.696 | 0.026 | 0.502 |
| Male 1:1 - Male 2:1 | 0.088 | -1.129 | 1.305 | 1.000 |
| Male 1:1 - Male 3:1 | -0.116 | -1.333 | 1.101 | 1.000 |
| Female 2:1 - Male 2:1 | -0.122 | -1.959 | 1.715 | 1.000 |
| Female 2:1 - Female 3:1 | -0.540 | -1.901 | 0.821 | 0.993 |
| Male 2:1 - Male 3:1 | -0.204 | -1.421 | 1.013 | 1.000 |
| Female 3:1 - Male 3:1 | 0.214 | -1.623 | 2.051 | 1.000 |
|  | **Enhanced sound perception AUC** | |  |  |
| Female 0:1 - Male 0:1 | 0.371 | -4.589 | 5.331 | 1.000 |
| Female 0:1 - Female 1:1 | -0.061 | -3.251 | 3.129 | 1.000 |
| Female 0:1 - Female 2:1 | -0.259 | -3.449 | 2.931 | 1.000 |
| Female 0:1 - Female 3:1 | -2.835 | -6.025 | 0.355 | 0.628 |
| Male 0:1 - Male 1:1 | -0.332 | -3.185 | 2.522 | 1.000 |
| Male 0:1 - Male 2:1 | -1.092 | -3.945 | 1.761 | 0.994 |
| Male 0:1 - Male 3:1 | -2.726 | -5.580 | 0.127 | 0.537 |
| Female 1:1 - Male 1:1 | 0.101 | -4.859 | 5.060 | 1.000 |
| Female 1:1 - Female 2:1 | -0.198 | -3.388 | 2.992 | 1.000 |
| Female 1:1 - Female 3:1 | -2.774 | -5.964 | 0.416 | 0.654 |
| Male 1:1 - Male 2:1 | -0.760 | -3.614 | 2.093 | 0.999 |
| Male 1:1 - Male 3:1 | -2.395 | -5.248 | 0.458 | 0.693 |
| Female 2:1 - Male 2:1 | -0.462 | -5.421 | 4.498 | 1.000 |
| Female 2:1 - Female 3:1 | -2.576 | -5.766 | 0.614 | 0.734 |
| Male 2:1 - Male 3:1 | -1.634 | -4.488 | 1.219 | 0.943 |
| Female 3:1 - Male 3:1 | 0.480 | -4.480 | 5.439 | 1.000 |
|  | **Enhanced sound perception peak** | |  |  |
| Female 0:1 - Male 0:1 | -0.127 | -1.823 | 1.569 | 1.000 |
| Female 0:1 - Female 1:1 | 0.370 | -0.957 | 1.697 | 0.999 |
| Female 0:1 - Female 2:1 | -0.310 | -1.637 | 1.017 | 1.000 |
| Female 0:1 - Female 3:1 | -1.310 | -2.637 | 0.017 | 0.494 |
| Male 0:1 - Male 1:1 | -0.056 | -1.243 | 1.131 | 1.000 |
| Male 0:1 - Male 2:1 | -0.448 | -1.635 | 0.739 | 0.995 |
| Male 0:1 - Male 3:1 | -0.380 | -1.567 | 0.807 | 0.998 |
| Female 1:1 - Male 1:1 | -0.553 | -2.249 | 1.143 | 0.998 |
| Female 1:1 - Female 2:1 | -0.680 | -2.007 | 0.647 | 0.969 |
| Female 1:1 - Female 3:1 | -1.680 | -3.007 | -0.353 | 0.185 |
| Male 1:1 - Male 2:1 | -0.392 | -1.579 | 0.795 | 0.998 |
| Male 1:1 - Male 3:1 | -0.324 | -1.511 | 0.863 | 0.999 |
| Female 2:1 - Male 2:1 | -0.265 | -1.961 | 1.431 | 1.000 |
| Female 2:1 - Female 3:1 | -1.000 | -2.327 | 0.327 | 0.797 |
| Male 2:1 - Male 3:1 | 0.068 | -1.119 | 1.255 | 1.000 |
| Female 3:1 - Male 3:1 | 0.803 | -0.893 | 2.499 | 0.980 |
|  | **Feel anxious AUC** | |  |  |
| Female 0:1 - Male 0:1 | -0.515 | -4.116 | 3.087 | 1.000 |
| Female 0:1 - Female 1:1 | -0.419 | -3.438 | 2.599 | 1.000 |
| Female 0:1 - Female 2:1 | -2.821 | -5.839 | 0.198 | 0.566 |
| Female 0:1 - Female 3:1 | -2.741 | -5.760 | 0.278 | 0.602 |
| Male 0:1 - Male 1:1 | 0.705 | -1.995 | 3.405 | 1.000 |
| Male 0:1 - Male 2:1 | -0.637 | -3.337 | 2.063 | 1.000 |
| Male 0:1 - Male 3:1 | -0.165 | -2.865 | 2.535 | 1.000 |
| Female 1:1 - Male 1:1 | 0.610 | -2.992 | 4.212 | 1.000 |
| Female 1:1 - Female 2:1 | -2.401 | -5.420 | 0.617 | 0.748 |
| Female 1:1 - Female 3:1 | -2.322 | -5.340 | 0.697 | 0.779 |
| Male 1:1 - Male 2:1 | -1.342 | -4.042 | 1.358 | 0.974 |
| Male 1:1 - Male 3:1 | -0.870 | -3.570 | 1.830 | 0.998 |
| Female 2:1 - Male 2:1 | 1.669 | -1.932 | 5.271 | 0.982 |
| Female 2:1 - Female 3:1 | 0.080 | -2.939 | 3.099 | 1.000 |
| Male 2:1 - Male 3:1 | 0.472 | -2.228 | 3.172 | 1.000 |
| Female 3:1 - Male 3:1 | 2.062 | -1.540 | 5.663 | 0.943 |
|  | **Feel anxious peak** | |  |  |
| Female 0:1 - Male 0:1 | 0.150 | -1.044 | 1.344 | 1.000 |
| Female 0:1 - Female 1:1 | 0.400 | -0.681 | 1.481 | 0.995 |
| Female 0:1 - Female 2:1 | -0.320 | -1.401 | 0.761 | 0.999 |
| Female 0:1 - Female 3:1 | 0.015 | -1.066 | 1.096 | 1.000 |
| Male 0:1 - Male 1:1 | 0.580 | -0.387 | 1.547 | 0.928 |
| Male 0:1 - Male 2:1 | 0.056 | -0.911 | 1.023 | 1.000 |
| Male 0:1 - Male 3:1 | 0.044 | -0.923 | 1.011 | 1.000 |
| Female 1:1 - Male 1:1 | 0.330 | -0.864 | 1.524 | 0.999 |
| Female 1:1 - Female 2:1 | -0.720 | -1.801 | 0.361 | 0.881 |
| Female 1:1 - Female 3:1 | -0.385 | -1.466 | 0.696 | 0.996 |
| Male 1:1 - Male 2:1 | -0.524 | -1.491 | 0.443 | 0.958 |
| Male 1:1 - Male 3:1 | -0.536 | -1.503 | 0.431 | 0.952 |
| Female 2:1 - Male 2:1 | 0.526 | -0.668 | 1.720 | 0.987 |
| Female 2:1 - Female 3:1 | 0.335 | -0.746 | 1.416 | 0.998 |
| Male 2:1 - Male 3:1 | -0.012 | -0.979 | 0.955 | 1.000 |
| Female 3:1 - Male 3:1 | 0.179 | -1.015 | 1.373 | 1.000 |
|  | **Feel calm and relaxed AUC** | |  |  |
| Female 0:1 - Male 0:1 | 0.869 | -4.812 | 6.549 | 1.000 |
| Female 0:1 - Female 1:1 | -3.921 | -8.437 | 0.595 | 0.656 |
| Female 0:1 - Female 2:1 | 3.373 | -1.143 | 7.889 | 0.804 |
| Female 0:1 - Female 3:1 | 2.084 | -2.433 | 6.600 | 0.983 |
| Male 0:1 - Male 1:1 | 1.106 | -2.934 | 5.145 | 0.999 |
| Male 0:1 - Male 2:1 | 1.166 | -2.873 | 5.206 | 0.999 |
| Male 0:1 - Male 3:1 | 2.510 | -1.529 | 6.550 | 0.915 |
| Female 1:1 - Male 1:1 | 5.896 | 0.215 | 11.576 | 0.428 |
| Female 1:1 - Female 2:1 | 7.294 | 2.778 | 11.811 | 0.031 |
| Female 1:1 - Female 3:1 | 6.005 | 1.488 | 10.521 | 0.139 |
| Male 1:1 - Male 2:1 | 0.060 | -3.979 | 4.100 | 1.000 |
| Male 1:1 - Male 3:1 | 1.404 | -2.635 | 5.444 | 0.997 |
| Female 2:1 - Male 2:1 | -1.338 | -7.019 | 4.343 | 1.000 |
| Female 2:1 - Female 3:1 | -1.289 | -5.806 | 3.227 | 0.999 |
| Male 2:1 - Male 3:1 | 1.344 | -2.696 | 5.383 | 0.998 |
| Female 3:1 - Male 3:1 | 1.295 | -4.385 | 6.976 | 1.000 |
|  | **VAS Feel calm and relaxed peak** | |  |  |
| Female 0:1 - Male 0:1 | 0.122 | -1.719 | 1.963 | 1.000 |
| Female 0:1 - Female 1:1 | -0.770 | -2.355 | 0.815 | 0.977 |
| Female 0:1 - Female 2:1 | 1.025 | -0.560 | 2.610 | 0.896 |
| Female 0:1 - Female 3:1 | 0.470 | -1.115 | 2.055 | 0.999 |
| Male 0:1 - Male 1:1 | -0.012 | -1.430 | 1.406 | 1.000 |
| Male 0:1 - Male 2:1 | 0.252 | -1.166 | 1.670 | 1.000 |
| Male 0:1 - Male 3:1 | 0.944 | -0.474 | 2.362 | 0.881 |
| Female 1:1 - Male 1:1 | 0.880 | -0.961 | 2.721 | 0.979 |
| Female 1:1 - Female 2:1 | 1.795 | 0.210 | 3.380 | 0.312 |
| Female 1:1 - Female 3:1 | 1.240 | -0.345 | 2.825 | 0.764 |
| Male 1:1 - Male 2:1 | 0.264 | -1.154 | 1.682 | 1.000 |
| Male 1:1 - Male 3:1 | 0.956 | -0.462 | 2.374 | 0.874 |
| Female 2:1 - Male 2:1 | -0.651 | -2.492 | 1.190 | 0.997 |
| Female 2:1 - Female 3:1 | -0.555 | -2.140 | 1.030 | 0.997 |
| Male 2:1 - Male 3:1 | 0.692 | -0.726 | 2.110 | 0.976 |
| Female 3:1 - Male 3:1 | 0.596 | -1.245 | 2.437 | 0.998 |
|  | **VAS Feel drug effect AUC** | |  |  |
| Female 0:1 - Male 0:1 | 1.418 | -1.406 | 4.242 | 0.972 |
| Female 0:1 - Female 1:1 | 0.037 | -1.926 | 2.000 | 1.000 |
| Female 0:1 - Female 2:1 | -0.127 | -2.090 | 1.836 | 1.000 |
| Female 0:1 - Female 3:1 | -0.939 | -2.902 | 1.024 | 0.979 |
| Male 0:1 - Male 1:1 | -1.540 | -3.296 | 0.216 | 0.644 |
| Male 0:1 - Male 2:1 | -0.987 | -2.743 | 0.768 | 0.948 |
| Male 0:1 - Male 3:1 | -0.888 | -2.643 | 0.868 | 0.971 |
| Female 1:1 - Male 1:1 | -0.159 | -2.983 | 2.665 | 1.000 |
| Female 1:1 - Female 2:1 | -0.164 | -2.127 | 1.799 | 1.000 |
| Female 1:1 - Female 3:1 | -0.976 | -2.939 | 0.987 | 0.973 |
| Male 1:1 - Male 2:1 | 0.552 | -1.203 | 2.308 | 0.998 |
| Male 1:1 - Male 3:1 | 0.652 | -1.104 | 2.408 | 0.995 |
| Female 2:1 - Male 2:1 | 0.557 | -2.267 | 3.381 | 1.000 |
| Female 2:1 - Female 3:1 | -0.812 | -2.775 | 1.151 | 0.991 |
| Male 2:1 - Male 3:1 | 0.100 | -1.656 | 1.856 | 1.000 |
| Female 3:1 - Male 3:1 | 1.469 | -1.355 | 4.293 | 0.965 |
|  | **VAS Feel drug effect peak** | |  |  |
| Female 0:1 - Male 0:1 | 0.435 | -0.439 | 1.309 | 0.973 |
| Female 0:1 - Female 1:1 | 0.240 | -0.466 | 0.946 | 0.997 |
| Female 0:1 - Female 2:1 | 0.035 | -0.671 | 0.741 | 1.000 |
| Female 0:1 - Female 3:1 | -0.315 | -1.021 | 0.391 | 0.986 |
| Male 0:1 - Male 1:1 | -0.024 | -0.655 | 0.607 | 1.000 |
| Male 0:1 - Male 2:1 | 0.080 | -0.551 | 0.711 | 1.000 |
| Male 0:1 - Male 3:1 | -0.188 | -0.819 | 0.443 | 0.999 |
| Female 1:1 - Male 1:1 | 0.171 | -0.703 | 1.045 | 1.000 |
| Female 1:1 - Female 2:1 | -0.205 | -0.911 | 0.501 | 0.999 |
| Female 1:1 - Female 3:1 | -0.555 | -1.261 | 0.151 | 0.759 |
| Male 1:1 - Male 2:1 | 0.104 | -0.527 | 0.735 | 1.000 |
| Male 1:1 - Male 3:1 | -0.164 | -0.795 | 0.467 | 1.000 |
| Female 2:1 - Male 2:1 | 0.480 | -0.394 | 1.354 | 0.954 |
| Female 2:1 - Female 3:1 | -0.350 | -1.056 | 0.356 | 0.974 |
| Male 2:1 - Male 3:1 | -0.268 | -0.899 | 0.363 | 0.989 |
| Female 3:1 - Male 3:1 | 0.562 | -0.312 | 1.436 | 0.899 |
|  | **VAS Feel hungry AUC** | |  |  |
| Female 0:1 - Male 0:1 | 1.668 | -1.323 | 4.660 | 0.950 |
| Female 0:1 - Female 1:1 | -0.815 | -2.810 | 1.180 | 0.992 |
| Female 0:1 - Female 2:1 | -0.393 | -2.388 | 1.602 | 1.000 |
| Female 0:1 - Female 3:1 | -1.988 | -3.983 | 0.006 | 0.481 |
| Male 0:1 - Male 1:1 | -1.760 | -3.544 | 0.024 | 0.495 |
| Male 0:1 - Male 2:1 | -1.463 | -3.248 | 0.321 | 0.718 |
| Male 0:1 - Male 3:1 | -1.703 | -3.487 | 0.081 | 0.538 |
| Female 1:1 - Male 1:1 | 0.723 | -2.268 | 3.715 | 1.000 |
| Female 1:1 - Female 2:1 | 0.422 | -1.573 | 2.417 | 1.000 |
| Female 1:1 - Female 3:1 | -1.173 | -3.168 | 0.821 | 0.935 |
| Male 1:1 - Male 2:1 | 0.296 | -1.488 | 2.081 | 1.000 |
| Male 1:1 - Male 3:1 | 0.057 | -1.727 | 1.841 | 1.000 |
| Female 2:1 - Male 2:1 | 0.598 | -2.394 | 3.589 | 1.000 |
| Female 2:1 - Female 3:1 | -1.595 | -3.590 | 0.399 | 0.743 |
| Male 2:1 - Male 3:1 | -0.240 | -2.024 | 1.544 | 1.000 |
| Female 3:1 - Male 3:1 | 1.953 | -1.038 | 4.945 | 0.891 |
|  | **VAS Feel hungry peak** | |  |  |
| Female 0:1 - Male 0:1 | 0.729 | -0.247 | 1.705 | 0.804 |
| Female 0:1 - Female 1:1 | -0.200 | -0.913 | 0.513 | 0.999 |
| Female 0:1 - Female 2:1 | 0.025 | -0.688 | 0.738 | 1.000 |
| Female 0:1 - Female 3:1 | -0.305 | -1.018 | 0.408 | 0.989 |
| Male 0:1 - Male 1:1 | -0.224 | -0.862 | 0.414 | 0.997 |
| Male 0:1 - Male 2:1 | -0.264 | -0.902 | 0.374 | 0.991 |
| Male 0:1 - Male 3:1 | -0.264 | -0.902 | 0.374 | 0.991 |
| Female 1:1 - Male 1:1 | 0.705 | -0.271 | 1.681 | 0.829 |
| Female 1:1 - Female 2:1 | 0.225 | -0.488 | 0.938 | 0.998 |
| Female 1:1 - Female 3:1 | -0.105 | -0.818 | 0.608 | 1.000 |
| Male 1:1 - Male 2:1 | -0.040 | -0.678 | 0.598 | 1.000 |
| Male 1:1 - Male 3:1 | -0.040 | -0.678 | 0.598 | 1.000 |
| Female 2:1 - Male 2:1 | 0.440 | -0.536 | 1.416 | 0.985 |
| Female 2:1 - Female 3:1 | -0.330 | -1.043 | 0.383 | 0.982 |
| Male 2:1 - Male 3:1 | 0.000 | -0.638 | 0.638 | 1.000 |
| Female 3:1 - Male 3:1 | 0.770 | -0.206 | 1.746 | 0.756 |
|  | **VAS Feel paranoid AUC** | |  |  |
| Female 0:1 - Male 0:1 | -1.093 | -4.366 | 2.181 | 0.998 |
| Female 0:1 - Female 1:1 | -2.262 | -4.904 | 0.381 | 0.672 |
| Female 0:1 - Female 2:1 | -4.358 | -7.051 | -1.664 | 0.031 |
| Female 0:1 - Female 3:1 | -2.748 | -5.419 | -0.077 | 0.439 |
| Male 0:1 - Male 1:1 | -0.290 | -2.680 | 2.101 | 1.000 |
| Male 0:1 - Male 2:1 | 0.207 | -2.184 | 2.597 | 1.000 |
| Male 0:1 - Male 3:1 | 1.422 | -0.954 | 3.798 | 0.929 |
| Female 1:1 - Male 1:1 | 0.879 | -2.395 | 4.153 | 0.999 |
| Female 1:1 - Female 2:1 | -2.096 | -4.789 | 0.597 | 0.768 |
| Female 1:1 - Female 3:1 | -0.487 | -3.158 | 2.184 | 1.000 |
| Male 1:1 - Male 2:1 | 0.497 | -1.894 | 2.887 | 1.000 |
| Male 1:1 - Male 3:1 | 1.712 | -0.664 | 4.088 | 0.831 |
| Female 2:1 - Male 2:1 | 3.472 | 0.157 | 6.786 | 0.415 |
| Female 2:1 - Female 3:1 | 1.609 | -1.089 | 4.307 | 0.930 |
| Male 2:1 - Male 3:1 | 1.215 | -1.160 | 3.591 | 0.969 |
| Female 3:1 - Male 3:1 | 3.078 | -0.165 | 6.320 | 0.546 |
|  | **VAS Feel paranoid peak** | |  |  |
| Female 0:1 - Male 0:1 | -0.060 | -1.118 | 0.998 | 1.000 |
| Female 0:1 - Female 1:1 | -0.211 | -1.090 | 0.668 | 1.000 |
| Female 0:1 - Female 2:1 | -0.961 | -1.857 | -0.066 | 0.383 |
| Female 0:1 - Female 3:1 | -0.290 | -1.178 | 0.598 | 0.998 |
| Male 0:1 - Male 1:1 | 0.005 | -0.791 | 0.800 | 1.000 |
| Male 0:1 - Male 2:1 | 0.373 | -0.422 | 1.168 | 0.981 |
| Male 0:1 - Male 3:1 | 0.436 | -0.354 | 1.226 | 0.953 |
| Female 1:1 - Male 1:1 | 0.156 | -0.903 | 1.214 | 1.000 |
| Female 1:1 - Female 2:1 | -0.750 | -1.646 | 0.145 | 0.696 |
| Female 1:1 - Female 3:1 | -0.079 | -0.967 | 0.809 | 1.000 |
| Male 1:1 - Male 2:1 | 0.368 | -0.427 | 1.163 | 0.982 |
| Male 1:1 - Male 3:1 | 0.431 | -0.359 | 1.222 | 0.956 |
| Female 2:1 - Male 2:1 | 1.274 | 0.202 | 2.346 | 0.255 |
| Female 2:1 - Female 3:1 | 0.671 | -0.226 | 1.568 | 0.802 |
| Male 2:1 - Male 3:1 | 0.063 | -0.727 | 0.853 | 1.000 |
| Female 3:1 - Male 3:1 | 0.666 | -0.382 | 1.714 | 0.904 |
|  | **VAS Feel stoned AUC** | |  |  |
| Female 0:1 - Male 0:1 | 0.653 | -3.167 | 4.473 | 1.000 |
| Female 0:1 - Female 1:1 | -0.567 | -3.061 | 1.928 | 1.000 |
| Female 0:1 - Female 2:1 | -0.594 | -3.088 | 1.901 | 1.000 |
| Female 0:1 - Female 3:1 | -2.492 | -4.987 | 0.002 | 0.478 |
| Male 0:1 - Male 1:1 | -1.716 | -3.947 | 0.515 | 0.779 |
| Male 0:1 - Male 2:1 | -1.228 | -3.459 | 1.004 | 0.954 |
| Male 0:1 - Male 3:1 | -0.854 | -3.085 | 1.377 | 0.994 |
| Female 1:1 - Male 1:1 | -0.496 | -4.316 | 3.324 | 1.000 |
| Female 1:1 - Female 2:1 | -0.027 | -2.521 | 2.468 | 1.000 |
| Female 1:1 - Female 3:1 | -1.926 | -4.420 | 0.569 | 0.776 |
| Male 1:1 - Male 2:1 | 0.488 | -1.743 | 2.720 | 1.000 |
| Male 1:1 - Male 3:1 | 0.862 | -1.369 | 3.093 | 0.994 |
| Female 2:1 - Male 2:1 | 0.019 | -3.801 | 3.839 | 1.000 |
| Female 2:1 - Female 3:1 | -1.899 | -4.394 | 0.596 | 0.788 |
| Male 2:1 - Male 3:1 | 0.374 | -1.858 | 2.605 | 1.000 |
| Female 3:1 - Male 3:1 | 2.292 | -1.528 | 6.112 | 0.927 |
|  | **VAS Feel stoned peak** | |  |  |
| Female 0:1 - Male 0:1 | 0.937 | -0.320 | 2.194 | 0.805 |
| Female 0:1 - Female 1:1 | 0.550 | -0.427 | 1.527 | 0.948 |
| Female 0:1 - Female 2:1 | 0.575 | -0.402 | 1.552 | 0.935 |
| Female 0:1 - Female 3:1 | -0.120 | -1.097 | 0.857 | 1.000 |
| Male 0:1 - Male 1:1 | -0.512 | -1.386 | 0.362 | 0.936 |
| Male 0:1 - Male 2:1 | -0.508 | -1.382 | 0.366 | 0.939 |
| Male 0:1 - Male 3:1 | -0.164 | -1.038 | 0.710 | 1.000 |
| Female 1:1 - Male 1:1 | -0.125 | -1.382 | 1.132 | 1.000 |
| Female 1:1 - Female 2:1 | 0.025 | -0.952 | 1.002 | 1.000 |
| Female 1:1 - Female 3:1 | -0.670 | -1.647 | 0.307 | 0.864 |
| Male 1:1 - Male 2:1 | 0.004 | -0.870 | 0.878 | 1.000 |
| Male 1:1 - Male 3:1 | 0.348 | -0.526 | 1.222 | 0.993 |
| Female 2:1 - Male 2:1 | -0.146 | -1.403 | 1.111 | 1.000 |
| Female 2:1 - Female 3:1 | -0.695 | -1.672 | 0.282 | 0.840 |
| Male 2:1 - Male 3:1 | 0.344 | -0.530 | 1.218 | 0.993 |
| Female 3:1 - Male 3:1 | 0.893 | -0.364 | 2.150 | 0.841 |
|  | **VAS Feel tired AUC** | |  |  |
| Female 0:1 - Male 0:1 | 1.163 | -3.192 | 5.518 | 0.999 |
| Female 0:1 - Female 1:1 | -1.408 | -5.156 | 2.340 | 0.995 |
| Female 0:1 - Female 2:1 | -2.521 | -6.269 | 1.228 | 0.876 |
| Female 0:1 - Female 3:1 | -4.156 | -7.904 | -0.407 | 0.339 |
| Male 0:1 - Male 1:1 | -0.084 | -3.436 | 3.269 | 1.000 |
| Male 0:1 - Male 2:1 | -0.508 | -3.861 | 2.845 | 1.000 |
| Male 0:1 - Male 3:1 | -1.330 | -4.682 | 2.023 | 0.993 |
| Female 1:1 - Male 1:1 | 2.487 | -1.868 | 6.843 | 0.944 |
| Female 1:1 - Female 2:1 | -1.113 | -4.861 | 2.636 | 0.999 |
| Female 1:1 - Female 3:1 | -2.748 | -6.496 | 1.001 | 0.819 |
| Male 1:1 - Male 2:1 | -0.424 | -3.777 | 2.928 | 1.000 |
| Male 1:1 - Male 3:1 | -1.246 | -4.599 | 2.107 | 0.995 |
| Female 2:1 - Male 2:1 | 3.176 | -1.180 | 7.531 | 0.823 |
| Female 2:1 - Female 3:1 | -1.635 | -5.384 | 2.113 | 0.987 |
| Male 2:1 - Male 3:1 | -0.822 | -4.174 | 2.531 | 1.000 |
| Female 3:1 - Male 3:1 | 3.989 | -0.366 | 8.344 | 0.591 |
|  | **VAS Feel tired peak** | |  |  |
| Female 0:1 - Male 0:1 | 1.263 | -0.228 | 2.754 | 0.683 |
| Female 0:1 - Female 1:1 | 0.260 | -1.090 | 1.610 | 1.000 |
| Female 0:1 - Female 2:1 | 0.135 | -1.215 | 1.485 | 1.000 |
| Female 0:1 - Female 3:1 | -0.545 | -1.895 | 0.805 | 0.992 |
| Male 0:1 - Male 1:1 | -0.620 | -1.827 | 0.587 | 0.968 |
| Male 0:1 - Male 2:1 | -0.184 | -1.391 | 1.023 | 1.000 |
| Male 0:1 - Male 3:1 | -0.512 | -1.719 | 0.695 | 0.989 |
| Female 1:1 - Male 1:1 | 0.383 | -1.108 | 1.874 | 1.000 |
| Female 1:1 - Female 2:1 | -0.125 | -1.475 | 1.225 | 1.000 |
| Female 1:1 - Female 3:1 | -0.805 | -2.155 | 0.545 | 0.930 |
| Male 1:1 - Male 2:1 | 0.436 | -0.771 | 1.643 | 0.996 |
| Male 1:1 - Male 3:1 | 0.108 | -1.099 | 1.315 | 1.000 |
| Female 2:1 - Male 2:1 | 0.944 | -0.547 | 2.435 | 0.907 |
| Female 2:1 - Female 3:1 | -0.680 | -2.030 | 0.670 | 0.971 |
| Male 2:1 - Male 3:1 | -0.328 | -1.535 | 0.879 | 0.999 |
| Female 3:1 - Male 3:1 | 1.296 | -0.195 | 2.787 | 0.654 |
|  | **VAS Like drug effect AUC** | |  |  |
| Female 0:1 - Male 0:1 | 0.833 | -3.110 | 4.777 | 1.000 |
| Female 0:1 - Female 1:1 | 1.840 | -1.476 | 5.155 | 0.952 |
| Female 0:1 - Female 2:1 | 3.981 | 0.665 | 7.296 | 0.242 |
| Female 0:1 - Female 3:1 | 2.274 | -1.041 | 5.590 | 0.864 |
| Male 0:1 - Male 1:1 | 0.033 | -2.932 | 2.999 | 1.000 |
| Male 0:1 - Male 2:1 | 0.774 | -2.191 | 3.740 | 1.000 |
| Male 0:1 - Male 3:1 | -1.239 | -4.205 | 1.726 | 0.990 |
| Female 1:1 - Male 1:1 | -0.973 | -4.917 | 2.970 | 1.000 |
| Female 1:1 - Female 2:1 | 2.141 | -1.174 | 5.457 | 0.897 |
| Female 1:1 - Female 3:1 | 0.435 | -2.881 | 3.750 | 1.000 |
| Male 1:1 - Male 2:1 | 0.741 | -2.224 | 3.707 | 1.000 |
| Male 1:1 - Male 3:1 | -1.273 | -4.238 | 1.693 | 0.989 |
| Female 2:1 - Male 2:1 | -2.373 | -6.317 | 1.570 | 0.927 |
| Female 2:1 - Female 3:1 | -1.707 | -5.022 | 1.609 | 0.968 |
| Male 2:1 - Male 3:1 | -2.014 | -4.979 | 0.952 | 0.870 |
| Female 3:1 - Male 3:1 | -2.681 | -6.624 | 1.263 | 0.869 |
|  | **VAS Like drug effect peak** | |  |  |
| Female 0:1 - Male 0:1 | 0.486 | -0.797 | 1.769 | 0.995 |
| Female 0:1 - Female 1:1 | 0.495 | -0.625 | 1.615 | 0.986 |
| Female 0:1 - Female 2:1 | 1.475 | 0.355 | 2.595 | 0.147 |
| Female 0:1 - Female 3:1 | 0.780 | -0.340 | 1.900 | 0.855 |
| Male 0:1 - Male 1:1 | 0.024 | -0.978 | 1.026 | 1.000 |
| Male 0:1 - Male 2:1 | 0.160 | -0.842 | 1.162 | 1.000 |
| Male 0:1 - Male 3:1 | -0.132 | -1.134 | 0.870 | 1.000 |
| Female 1:1 - Male 1:1 | 0.015 | -1.268 | 1.298 | 1.000 |
| Female 1:1 - Female 2:1 | 0.980 | -0.140 | 2.100 | 0.646 |
| Female 1:1 - Female 3:1 | 0.285 | -0.835 | 1.405 | 1.000 |
| Male 1:1 - Male 2:1 | 0.136 | -0.866 | 1.138 | 1.000 |
| Male 1:1 - Male 3:1 | -0.156 | -1.158 | 0.846 | 1.000 |
| Female 2:1 - Male 2:1 | -0.829 | -2.112 | 0.454 | 0.897 |
| Female 2:1 - Female 3:1 | -0.695 | -1.815 | 0.425 | 0.915 |
| Male 2:1 - Male 3:1 | -0.292 | -1.294 | 0.710 | 0.999 |
| Female 3:1 - Male 3:1 | -0.426 | -1.709 | 0.857 | 0.998 |
|  | **VAS Mentally impaired AUC** | |  |  |
| Female 0:1 - Male 0:1 | 0.952 | -3.312 | 5.216 | 1.000 |
| Female 0:1 - Female 1:1 | 0.073 | -2.733 | 2.878 | 1.000 |
| Female 0:1 - Female 2:1 | -0.274 | -3.079 | 2.531 | 1.000 |
| Female 0:1 - Female 3:1 | -3.355 | -6.160 | -0.549 | 0.246 |
| Male 0:1 - Male 1:1 | -1.346 | -3.855 | 1.163 | 0.960 |
| Male 0:1 - Male 2:1 | -2.682 | -5.191 | -0.173 | 0.387 |
| Male 0:1 - Male 3:1 | -2.153 | -4.662 | 0.356 | 0.669 |
| Female 1:1 - Male 1:1 | -0.467 | -4.731 | 3.798 | 1.000 |
| Female 1:1 - Female 2:1 | -0.347 | -3.152 | 2.459 | 1.000 |
| Female 1:1 - Female 3:1 | -3.428 | -6.233 | -0.622 | 0.222 |
| Male 1:1 - Male 2:1 | -1.336 | -3.845 | 1.173 | 0.961 |
| Male 1:1 - Male 3:1 | -0.807 | -3.316 | 1.702 | 0.998 |
| Female 2:1 - Male 2:1 | -1.456 | -5.720 | 2.808 | 0.997 |
| Female 2:1 - Female 3:1 | -3.081 | -5.886 | -0.276 | 0.352 |
| Male 2:1 - Male 3:1 | 0.529 | -1.980 | 3.038 | 1.000 |
| Female 3:1 - Male 3:1 | 2.154 | -2.110 | 6.418 | 0.971 |
|  | **VAS Mentally impaired peak** | |  |  |
| Female 0:1 - Male 0:1 | 0.540 | -1.061 | 2.141 | 0.997 |
| Female 0:1 - Female 1:1 | -0.005 | -1.128 | 1.118 | 1.000 |
| Female 0:1 - Female 2:1 | 0.770 | -0.353 | 1.893 | 0.864 |
| Female 0:1 - Female 3:1 | -0.850 | -1.973 | 0.273 | 0.793 |
| Male 0:1 - Male 1:1 | -0.408 | -1.413 | 0.597 | 0.992 |
| Male 0:1 - Male 2:1 | -0.784 | -1.789 | 0.221 | 0.766 |
| Male 0:1 - Male 3:1 | -0.768 | -1.773 | 0.237 | 0.784 |
| Female 1:1 - Male 1:1 | 0.137 | -1.464 | 1.738 | 1.000 |
| Female 1:1 - Female 2:1 | 0.775 | -0.348 | 1.898 | 0.860 |
| Female 1:1 - Female 3:1 | -0.845 | -1.968 | 0.278 | 0.798 |
| Male 1:1 - Male 2:1 | -0.376 | -1.381 | 0.629 | 0.995 |
| Male 1:1 - Male 3:1 | -0.360 | -1.365 | 0.645 | 0.996 |
| Female 2:1 - Male 2:1 | -1.014 | -2.615 | 0.587 | 0.905 |
| Female 2:1 - Female 3:1 | -1.620 | -2.743 | -0.497 | 0.080 |
| Male 2:1 - Male 3:1 | 0.016 | -0.989 | 1.021 | 1.000 |
| Female 3:1 - Male 3:1 | 0.622 | -0.979 | 2.223 | 0.994 |
|  | **VAS Want alcohol AUC** | |  |  |
| Female 0:1 - Male 0:1 | 0.492 | -2.676 | 3.660 | 1.000 |
| Female 0:1 - Female 1:1 | 0.996 | -1.577 | 3.569 | 0.994 |
| Female 0:1 - Female 2:1 | 0.589 | -1.983 | 3.162 | 1.000 |
| Female 0:1 - Female 3:1 | 1.368 | -1.205 | 3.941 | 0.962 |
| Male 0:1 - Male 1:1 | -0.071 | -2.372 | 2.230 | 1.000 |
| Male 0:1 - Male 2:1 | -0.265 | -2.566 | 2.036 | 1.000 |
| Male 0:1 - Male 3:1 | -0.452 | -2.753 | 1.850 | 1.000 |
| Female 1:1 - Male 1:1 | -0.575 | -3.743 | 2.593 | 1.000 |
| Female 1:1 - Female 2:1 | -0.407 | -2.979 | 2.166 | 1.000 |
| Female 1:1 - Female 3:1 | 0.372 | -2.201 | 2.945 | 1.000 |
| Male 1:1 - Male 2:1 | -0.194 | -2.495 | 2.107 | 1.000 |
| Male 1:1 - Male 3:1 | -0.381 | -2.682 | 1.920 | 1.000 |
| Female 2:1 - Male 2:1 | -0.362 | -3.530 | 2.806 | 1.000 |
| Female 2:1 - Female 3:1 | 0.779 | -1.794 | 3.351 | 0.999 |
| Male 2:1 - Male 3:1 | -0.187 | -2.488 | 2.114 | 1.000 |
| Female 3:1 - Male 3:1 | -1.328 | -4.496 | 1.841 | 0.990 |
|  | **VAS Want alcohol peak** | |  |  |
| Female 0:1 - Male 0:1 | 0.358 | -0.599 | 1.315 | 0.995 |
| Female 0:1 - Female 1:1 | 0.420 | -0.351 | 1.191 | 0.956 |
| Female 0:1 - Female 2:1 | -0.150 | -0.921 | 0.621 | 1.000 |
| Female 0:1 - Female 3:1 | 0.140 | -0.631 | 0.911 | 1.000 |
| Male 0:1 - Male 1:1 | -0.284 | -0.973 | 0.405 | 0.991 |
| Male 0:1 - Male 2:1 | -0.412 | -1.101 | 0.277 | 0.929 |
| Male 0:1 - Male 3:1 | -0.116 | -0.805 | 0.573 | 1.000 |
| Female 1:1 - Male 1:1 | -0.346 | -1.303 | 0.611 | 0.996 |
| Female 1:1 - Female 2:1 | -0.570 | -1.341 | 0.201 | 0.812 |
| Female 1:1 - Female 3:1 | -0.280 | -1.051 | 0.491 | 0.996 |
| Male 1:1 - Male 2:1 | -0.128 | -0.817 | 0.561 | 1.000 |
| Male 1:1 - Male 3:1 | 0.168 | -0.521 | 0.857 | 1.000 |
| Female 2:1 - Male 2:1 | 0.096 | -0.861 | 1.053 | 1.000 |
| Female 2:1 - Female 3:1 | 0.290 | -0.481 | 1.061 | 0.995 |
| Male 2:1 - Male 3:1 | 0.296 | -0.393 | 0.985 | 0.989 |
| Female 3:1 - Male 3:1 | 0.102 | -0.855 | 1.059 | 1.000 |
|  | **VAS Want food AUC** | |  |  |
| Female 0:1 - Male 0:1 | -1.614 | -6.106 | 2.878 | 0.996 |
| Female 0:1 - Female 1:1 | -1.182 | -5.203 | 2.838 | 0.999 |
| Female 0:1 - Female 2:1 | -0.130 | -4.150 | 3.891 | 1.000 |
| Female 0:1 - Female 3:1 | -1.384 | -5.405 | 2.636 | 0.997 |
| Male 0:1 - Male 1:1 | -0.601 | -4.197 | 2.995 | 1.000 |
| Male 0:1 - Male 2:1 | 2.452 | -1.144 | 6.048 | 0.868 |
| Male 0:1 - Male 3:1 | -0.653 | -4.250 | 2.943 | 1.000 |
| Female 1:1 - Male 1:1 | -1.032 | -5.525 | 3.460 | 1.000 |
| Female 1:1 - Female 2:1 | 1.053 | -2.968 | 5.073 | 0.999 |
| Female 1:1 - Female 3:1 | -0.202 | -4.223 | 3.819 | 1.000 |
| Male 1:1 - Male 2:1 | 3.053 | -0.543 | 6.649 | 0.681 |
| Male 1:1 - Male 3:1 | -0.052 | -3.649 | 3.544 | 1.000 |
| Female 2:1 - Male 2:1 | 0.968 | -3.524 | 5.460 | 1.000 |
| Female 2:1 - Female 3:1 | -1.255 | -5.275 | 2.766 | 0.998 |
| Male 2:1 - Male 3:1 | -3.106 | -6.702 | 0.491 | 0.662 |
| Female 3:1 - Male 3:1 | -0.883 | -5.375 | 3.610 | 1.000 |
|  | **VAS Want food peak** | |  |  |
| Female 0:1 - Male 0:1 | 0.356 | -1.139 | 1.851 | 1.000 |
| Female 0:1 - Female 1:1 | 0.270 | -1.084 | 1.624 | 1.000 |
| Female 0:1 - Female 2:1 | 0.365 | -0.989 | 1.719 | 0.999 |
| Female 0:1 - Female 3:1 | 0.185 | -1.169 | 1.539 | 1.000 |
| Male 0:1 - Male 1:1 | -0.732 | -1.943 | 0.479 | 0.925 |
| Male 0:1 - Male 2:1 | 0.320 | -0.891 | 1.531 | 0.999 |
| Male 0:1 - Male 3:1 | -0.880 | -2.091 | 0.331 | 0.825 |
| Female 1:1 - Male 1:1 | -0.646 | -2.141 | 0.849 | 0.988 |
| Female 1:1 - Female 2:1 | 0.095 | -1.259 | 1.449 | 1.000 |
| Female 1:1 - Female 3:1 | -0.085 | -1.439 | 1.269 | 1.000 |
| Male 1:1 - Male 2:1 | 1.052 | -0.159 | 2.263 | 0.654 |
| Male 1:1 - Male 3:1 | -0.148 | -1.359 | 1.063 | 1.000 |
| Female 2:1 - Male 2:1 | 0.311 | -1.184 | 1.806 | 1.000 |
| Female 2:1 - Female 3:1 | -0.180 | -1.534 | 1.174 | 1.000 |
| Male 2:1 - Male 3:1 | -1.200 | -2.411 | 0.011 | 0.488 |
| Female 3:1 - Male 3:1 | -0.709 | -2.204 | 0.786 | 0.980 |
|  | **VAS Want more drug AUC** | |  |  |
| Female 0:1 - Male 0:1 | -0.105 | -5.433 | 5.222 | 1.000 |
| Female 0:1 - Female 1:1 | 0.867 | -2.603 | 4.337 | 1.000 |
| Female 0:1 - Female 2:1 | 3.476 | 0.005 | 6.946 | 0.474 |
| Female 0:1 - Female 3:1 | 2.604 | -0.867 | 6.074 | 0.800 |
| Male 0:1 - Male 1:1 | -1.306 | -4.409 | 1.798 | 0.990 |
| Male 0:1 - Male 2:1 | -0.238 | -3.342 | 2.866 | 1.000 |
| Male 0:1 - Male 3:1 | -1.154 | -4.258 | 1.950 | 0.995 |
| Female 1:1 - Male 1:1 | -2.278 | -7.605 | 3.050 | 0.989 |
| Female 1:1 - Female 2:1 | 2.609 | -0.861 | 6.079 | 0.799 |
| Female 1:1 - Female 3:1 | 1.737 | -1.733 | 5.207 | 0.973 |
| Male 1:1 - Male 2:1 | 1.068 | -2.036 | 4.172 | 0.997 |
| Male 1:1 - Male 3:1 | 0.151 | -2.952 | 3.255 | 1.000 |
| Female 2:1 - Male 2:1 | -3.819 | -9.146 | 1.509 | 0.834 |
| Female 2:1 - Female 3:1 | -0.872 | -4.342 | 2.598 | 1.000 |
| Male 2:1 - Male 3:1 | -0.916 | -4.020 | 2.188 | 0.999 |
| Female 3:1 - Male 3:1 | -3.863 | -9.190 | 1.464 | 0.826 |
|  | **VAS Want more drug peak** | |  |  |
| Female 0:1 - Male 0:1 | -0.425 | -2.181 | 1.331 | 1.000 |
| Female 0:1 - Female 1:1 | -0.080 | -1.332 | 1.172 | 1.000 |
| Female 0:1 - Female 2:1 | 0.630 | -0.622 | 1.882 | 0.972 |
| Female 0:1 - Female 3:1 | 0.250 | -1.002 | 1.502 | 1.000 |
| Male 0:1 - Male 1:1 | 0.012 | -1.108 | 1.132 | 1.000 |
| Male 0:1 - Male 2:1 | 0.084 | -1.036 | 1.204 | 1.000 |
| Male 0:1 - Male 3:1 | -0.084 | -1.204 | 1.036 | 1.000 |
| Female 1:1 - Male 1:1 | -0.333 | -2.089 | 1.423 | 1.000 |
| Female 1:1 - Female 2:1 | 0.710 | -0.542 | 1.962 | 0.946 |
| Female 1:1 - Female 3:1 | 0.330 | -0.922 | 1.582 | 0.999 |
| Male 1:1 - Male 2:1 | 0.072 | -1.048 | 1.192 | 1.000 |
| Male 1:1 - Male 3:1 | -0.096 | -1.216 | 1.024 | 1.000 |
| Female 2:1 - Male 2:1 | -0.971 | -2.727 | 0.785 | 0.952 |
| Female 2:1 - Female 3:1 | -0.380 | -1.632 | 0.872 | 0.999 |
| Male 2:1 - Male 3:1 | -0.168 | -1.288 | 0.952 | 1.000 |
| Female 3:1 - Male 3:1 | -0.759 | -2.515 | 0.997 | 0.988 |
|  | **Chocolate** | |  |  |
| Female 0:1 - Male 0:1 | -0.801 | -1.570 | -0.032 | 0.428 |
| Female 0:1 - Female 1:1 | -0.123 | -0.549 | 0.303 | 0.999 |
| Female 0:1 - Female 2:1 | 0.171 | -0.255 | 0.597 | 0.992 |
| Female 0:1 - Female 3:1 | -0.220 | -0.646 | 0.206 | 0.967 |
| Male 0:1 - Male 1:1 | -0.246 | -1.098 | 0.605 | 0.999 |
| Male 0:1 - Male 2:1 | 0.342 | -0.509 | 1.194 | 0.992 |
| Male 0:1 - Male 3:1 | -0.440 | -1.291 | 0.412 | 0.967 |
| Female 1:1 - Male 1:1 | -0.924 | -1.693 | -0.155 | 0.252 |
| Female 1:1 - Female 2:1 | 0.294 | -0.131 | 0.720 | 0.859 |
| Female 1:1 - Female 3:1 | -0.097 | -0.522 | 0.329 | 1.000 |
| Male 1:1 - Male 2:1 | 0.588 | -0.263 | 1.440 | 0.859 |
| Male 1:1 - Male 3:1 | -0.193 | -1.045 | 0.658 | 1.000 |
| Female 2:1 - Male 2:1 | -0.630 | -1.399 | 0.139 | 0.718 |
| Female 2:1 - Female 3:1 | -0.391 | -0.817 | 0.035 | 0.588 |
| Male 2:1 - Male 3:1 | -0.782 | -1.633 | 0.070 | 0.588 |
| Female 3:1 - Male 3:1 | -1.021 | -1.790 | -0.252 | 0.152 |
|  | **Systolic blood pressure AUC** | |  |  |
| Female 0:1 - Male 0:1 | -4.275 | -32.040 | 23.490 | 1.000 |
| Female 0:1 - Female 1:1 | -0.736 | -25.299 | 23.828 | 1.000 |
| Female 0:1 - Female 2:1 | 12.179 | -12.384 | 36.743 | 0.974 |
| Female 0:1 - Female 3:1 | 17.643 | -6.921 | 42.206 | 0.834 |
| Male 0:1 - Male 1:1 | 16.067 | -6.446 | 38.580 | 0.838 |
| Male 0:1 - Male 2:1 | 2.601 | -19.912 | 25.114 | 1.000 |
| Male 0:1 - Male 3:1 | 22.796 | 0.283 | 45.309 | 0.460 |
| Female 1:1 - Male 1:1 | 12.528 | -15.237 | 40.293 | 0.985 |
| Female 1:1 - Female 2:1 | 12.915 | -11.649 | 37.478 | 0.964 |
| Female 1:1 - Female 3:1 | 18.378 | -6.185 | 42.942 | 0.802 |
| Male 1:1 - Male 2:1 | -13.466 | -35.979 | 9.047 | 0.929 |
| Male 1:1 - Male 3:1 | 6.728 | -15.784 | 29.241 | 0.999 |
| Female 2:1 - Male 2:1 | -13.854 | -41.619 | 13.911 | 0.973 |
| Female 2:1 - Female 3:1 | 5.464 | -19.100 | 30.027 | 1.000 |
| Male 2:1 - Male 3:1 | 20.195 | -2.318 | 42.708 | 0.617 |
| Female 3:1 - Male 3:1 | 0.877 | -26.887 | 28.642 | 1.000 |
|  | **Systolic blood pressure peak** | |  |  |
| Female 0:1 - Male 0:1 | -0.491 | -7.883 | 6.900 | 1.000 |
| Female 0:1 - Female 1:1 | -0.762 | -6.934 | 5.411 | 1.000 |
| Female 0:1 - Female 2:1 | 1.952 | -4.220 | 8.125 | 0.998 |
| Female 0:1 - Female 3:1 | 3.429 | -2.744 | 9.601 | 0.952 |
| Male 0:1 - Male 1:1 | 7.000 | 1.343 | 12.657 | 0.208 |
| Male 0:1 - Male 2:1 | 2.840 | -2.817 | 8.497 | 0.972 |
| Male 0:1 - Male 3:1 | 7.200 | 1.543 | 12.857 | 0.179 |
| Female 1:1 - Male 1:1 | 7.270 | -0.121 | 14.662 | 0.499 |
| Female 1:1 - Female 2:1 | 2.714 | -3.458 | 8.887 | 0.987 |
| Female 1:1 - Female 3:1 | 4.190 | -1.982 | 10.363 | 0.870 |
| Male 1:1 - Male 2:1 | -4.160 | -9.817 | 1.497 | 0.816 |
| Male 1:1 - Male 3:1 | 0.200 | -5.457 | 5.857 | 1.000 |
| Female 2:1 - Male 2:1 | 0.396 | -6.995 | 7.787 | 1.000 |
| Female 2:1 - Female 3:1 | 1.476 | -4.696 | 7.649 | 1.000 |
| Male 2:1 - Male 3:1 | 4.360 | -1.297 | 10.017 | 0.777 |
| Female 3:1 - Male 3:1 | 3.280 | -4.111 | 10.671 | 0.986 |
|  | **Diastolic blood pressure AUC** | |  |  |
| Female 0:1 - Male 0:1 | -10.197 | -29.409 | 9.015 | 0.962 |
| Female 0:1 - Female 1:1 | -8.386 | -26.387 | 9.616 | 0.982 |
| Female 0:1 - Female 2:1 | -17.583 | -35.584 | 0.419 | 0.508 |
| Female 0:1 - Female 3:1 | -10.877 | -28.879 | 7.124 | 0.926 |
| Male 0:1 - Male 1:1 | 5.928 | -10.570 | 22.427 | 0.996 |
| Male 0:1 - Male 2:1 | -1.092 | -17.590 | 15.407 | 1.000 |
| Male 0:1 - Male 3:1 | 14.341 | -2.158 | 30.840 | 0.654 |
| Female 1:1 - Male 1:1 | 4.117 | -15.095 | 23.330 | 1.000 |
| Female 1:1 - Female 2:1 | -9.197 | -27.198 | 8.805 | 0.969 |
| Female 1:1 - Female 3:1 | -2.491 | -20.493 | 15.510 | 1.000 |
| Male 1:1 - Male 2:1 | -7.020 | -23.519 | 9.479 | 0.989 |
| Male 1:1 - Male 3:1 | 8.413 | -8.086 | 24.911 | 0.970 |
| Female 2:1 - Male 2:1 | 6.294 | -12.918 | 25.507 | 0.998 |
| Female 2:1 - Female 3:1 | 6.706 | -11.296 | 24.707 | 0.995 |
| Male 2:1 - Male 3:1 | 15.433 | -1.066 | 31.931 | 0.564 |
| Female 3:1 - Male 3:1 | 15.021 | -4.191 | 34.234 | 0.765 |
|  | **Diastolic blood pressure peak** | |  |  |
| Female 0:1 - Male 0:1 | -0.830 | -6.242 | 4.581 | 1.000 |
| Female 0:1 - Female 1:1 | 0.381 | -4.260 | 5.022 | 1.000 |
| Female 0:1 - Female 2:1 | -4.476 | -9.117 | 0.165 | 0.524 |
| Female 0:1 - Female 3:1 | -0.476 | -5.117 | 4.165 | 1.000 |
| Male 0:1 - Male 1:1 | 2.080 | -2.173 | 6.333 | 0.976 |
| Male 0:1 - Male 2:1 | -1.480 | -5.733 | 2.773 | 0.997 |
| Male 0:1 - Male 3:1 | 3.040 | -1.213 | 7.293 | 0.837 |
| Female 1:1 - Male 1:1 | 0.869 | -4.543 | 6.280 | 1.000 |
| Female 1:1 - Female 2:1 | -4.857 | -9.498 | -0.216 | 0.415 |
| Female 1:1 - Female 3:1 | -0.857 | -5.498 | 3.784 | 1.000 |
| Male 1:1 - Male 2:1 | -3.560 | -7.813 | 0.693 | 0.696 |
| Male 1:1 - Male 3:1 | 0.960 | -3.293 | 5.213 | 1.000 |
| Female 2:1 - Male 2:1 | 2.166 | -3.246 | 7.577 | 0.993 |
| Female 2:1 - Female 3:1 | 4.000 | -0.641 | 8.641 | 0.664 |
| Male 2:1 - Male 3:1 | 4.520 | 0.267 | 8.773 | 0.395 |
| Female 3:1 - Male 3:1 | 2.686 | -2.726 | 8.097 | 0.974 |
|  | **Heart rate AUC** | |  |  |
| Female 0:1 - Male 0:1 | -10.572 | -43.512 | 22.368 | 0.998 |
| Female 0:1 - Female 1:1 | -13.664 | -40.810 | 13.482 | 0.972 |
| Female 0:1 - Female 2:1 | -29.776 | -56.922 | -2.630 | 0.353 |
| Female 0:1 - Female 3:1 | -0.749 | -27.896 | 26.397 | 1.000 |
| Male 0:1 - Male 1:1 | -11.303 | -36.183 | 13.577 | 0.984 |
| Male 0:1 - Male 2:1 | -20.585 | -45.465 | 4.295 | 0.709 |
| Male 0:1 - Male 3:1 | 1.789 | -23.090 | 26.669 | 1.000 |
| Female 1:1 - Male 1:1 | -8.211 | -41.151 | 24.729 | 1.000 |
| Female 1:1 - Female 2:1 | -16.112 | -43.258 | 11.035 | 0.932 |
| Female 1:1 - Female 3:1 | 12.915 | -14.231 | 40.061 | 0.979 |
| Male 1:1 - Male 2:1 | -9.282 | -34.162 | 15.598 | 0.995 |
| Male 1:1 - Male 3:1 | 13.093 | -11.787 | 37.973 | 0.964 |
| Female 2:1 - Male 2:1 | -1.381 | -34.321 | 31.559 | 1.000 |
| Female 2:1 - Female 3:1 | 29.026 | 1.880 | 56.172 | 0.387 |
| Male 2:1 - Male 3:1 | 22.375 | -2.505 | 47.255 | 0.614 |
| Female 3:1 - Male 3:1 | -8.033 | -40.973 | 24.907 | 1.000 |
|  | **Heart rate peak** | |  |  |
| Female 0:1 - Male 0:1 | -1.190 | -10.345 | 7.964 | 1.000 |
| Female 0:1 - Female 1:1 | -0.952 | -7.953 | 6.049 | 1.000 |
| Female 0:1 - Female 2:1 | -7.810 | -14.811 | -0.808 | 0.331 |
| Female 0:1 - Female 3:1 | 1.190 | -5.811 | 8.192 | 1.000 |
| Male 0:1 - Male 1:1 | -1.760 | -8.177 | 4.657 | 0.999 |
| Male 0:1 - Male 2:1 | -3.840 | -10.257 | 2.577 | 0.929 |
| Male 0:1 - Male 3:1 | 0.240 | -6.177 | 6.657 | 1.000 |
| Female 1:1 - Male 1:1 | -1.998 | -11.153 | 7.157 | 1.000 |
| Female 1:1 - Female 2:1 | -6.857 | -13.858 | 0.144 | 0.504 |
| Female 1:1 - Female 3:1 | 2.143 | -4.858 | 9.144 | 0.999 |
| Male 1:1 - Male 2:1 | -2.080 | -8.497 | 4.337 | 0.998 |
| Male 1:1 - Male 3:1 | 2.000 | -4.417 | 8.417 | 0.998 |
| Female 2:1 - Male 2:1 | 2.779 | -6.376 | 11.934 | 0.999 |
| Female 2:1 - Female 3:1 | 9.000 | 1.999 | 16.001 | 0.169 |
| Male 2:1 - Male 3:1 | 4.080 | -2.337 | 10.497 | 0.904 |
| Female 3:1 - Male 3:1 | -2.141 | -11.296 | 7.014 | 1.000 |
|  | **Temperature AUC** | |  |  |
| Female 0:1 - Male 0:1 | -10.337 | -29.082 | 8.407 | 0.954 |
| Female 0:1 - Female 1:1 | -8.430 | -25.940 | 9.080 | 0.978 |
| Female 0:1 - Female 2:1 | -17.511 | -35.021 | -0.001 | 0.476 |
| Female 0:1 - Female 3:1 | -10.610 | -28.121 | 6.900 | 0.925 |
| Male 0:1 - Male 1:1 | 5.908 | -10.140 | 21.957 | 0.996 |
| Male 0:1 - Male 2:1 | -1.081 | -17.130 | 14.967 | 1.000 |
| Male 0:1 - Male 3:1 | 13.706 | -2.342 | 29.754 | 0.674 |
| Female 1:1 - Male 1:1 | 4.001 | -14.744 | 22.745 | 1.000 |
| Female 1:1 - Female 2:1 | -9.081 | -26.591 | 8.429 | 0.967 |
| Female 1:1 - Female 3:1 | -2.181 | -19.691 | 15.330 | 1.000 |
| Male 1:1 - Male 2:1 | -6.990 | -23.038 | 9.059 | 0.988 |
| Male 1:1 - Male 3:1 | 7.798 | -8.251 | 23.846 | 0.977 |
| Female 2:1 - Male 2:1 | 6.092 | -12.652 | 24.837 | 0.998 |
| Female 2:1 - Female 3:1 | 6.900 | -10.610 | 24.411 | 0.993 |
| Male 2:1 - Male 3:1 | 14.787 | -1.261 | 30.836 | 0.583 |
| Female 3:1 - Male 3:1 | 13.979 | -4.765 | 32.724 | 0.805 |
|  | **Temperature peak** | |  |  |
| Female 0:1 - Male 0:1 | -0.121 | -0.297 | 0.056 | 0.866 |
| Female 0:1 - Female 1:1 | -0.048 | -0.225 | 0.130 | 0.999 |
| Female 0:1 - Female 2:1 | -0.124 | -0.301 | 0.054 | 0.854 |
| Female 0:1 - Female 3:1 | 0.010 | -0.168 | 0.187 | 1.000 |
| Male 0:1 - Male 1:1 | 0.068 | -0.095 | 0.231 | 0.990 |
| Male 0:1 - Male 2:1 | -0.008 | -0.171 | 0.155 | 1.000 |
| Male 0:1 - Male 3:1 | -0.056 | -0.219 | 0.107 | 0.997 |
| Female 1:1 - Male 1:1 | -0.005 | -0.181 | 0.171 | 1.000 |
| Female 1:1 - Female 2:1 | -0.076 | -0.254 | 0.101 | 0.989 |
| Female 1:1 - Female 3:1 | 0.057 | -0.120 | 0.235 | 0.998 |
| Male 1:1 - Male 2:1 | -0.076 | -0.239 | 0.087 | 0.981 |
| Male 1:1 - Male 3:1 | -0.124 | -0.287 | 0.039 | 0.787 |
| Female 2:1 - Male 2:1 | -0.005 | -0.181 | 0.171 | 1.000 |
| Female 2:1 - Female 3:1 | 0.133 | -0.044 | 0.311 | 0.800 |
| Male 2:1 - Male 3:1 | -0.048 | -0.211 | 0.115 | 0.999 |
| Female 3:1 - Male 3:1 | -0.186 | -0.362 | -0.010 | 0.401 |
|  | **Inhalation** | |  |  |
| Female 0:1 - Male 0:1 | 4.766 | -0.375 | 9.906 | 0.577 |
| Female 0:1 - Female 1:1 | -4.905 | -8.114 | -1.696 | 0.05 |
| **Female 0:1 - Female 2:1** | **-9.238** | **-12.447** | **-6.029** | **1.30x10^-6^** |
| **Female 0:1 - Female 3:1** | **-10.095** | **-13.304** | **-6.886** | **9.53x10^-8^** |
| Male 0:1 - Male 1:1 | -4.680 | -7.621 | -1.739 | 0.035 |
| **Male 0:1 - Male 2:1** | **-7.760** | **-10.701** | **-4.819** | **1.21x10^-5^** |
| **Male 0:1 - Male 3:1** | **-10.600** | **-13.541** | **-7.659** | **8.38x10^-10^** |
| Female 1:1 - Male 1:1 | 4.990 | -0.150 | 10.131 | 0.518 |
| Female 1:1 - Female 2:1 | -4.333 | -7.542 | -1.124 | 0.126 |
| Female 1:1 - Female 3:1 | -5.190 | -8.399 | -1.982 | 0.03 |
| Male 1:1 - Male 2:1 | -3.080 | -6.021 | -0.139 | 0.415 |
| **Male 1:1 - Male 3:1** | **-5.920** | **-8.861** | **-2.979** | **0.002** |
| Female 2:1 - Male 2:1 | 6.244 | 1.104 | 11.384 | 0.234 |
| Female 2:1 - Female 3:1 | -0.857 | -4.066 | 2.352 | 0.999 |
| Male 2:1 - Male 3:1 | -2.840 | -5.781 | 0.101 | 0.523 |
| Female 3:1 - Male 3:1 | 4.261 | -0.879 | 9.401 | 0.706 |
|  | **Coughing** | |  |  |
| Female 0:1 - Male 0:1 | -0.260 | -1.416 | 0.896 | 1 |
| Female 0:1 - Female 1:1 | -1.500 | -2.388 | -0.612 | 0.019 |
| **Female 0:1 - Female 2:1** | **-1.719** | **-2.607** | **-0.831** | **0.004** |
| **Female 0:1 - Female 3:1** | **-2.424** | **-3.312** | **-1.536** | **5.24 x10^-6^** |
| Male 0:1 - Male 1:1 | -0.840 | -1.654 | -0.026 | 0.434 |
| **Male 0:1 - Male 2:1** | **-1.664** | **-2.478** | **-0.850** | **0.002** |
| **Male 0:1 - Male 3:1** | **-2.436** | **-3.250** | **-1.622** | **4.29x10^-7^** |
| Female 1:1 - Male 1:1 | 0.400 | -0.756 | 1.556 | 0.997 |
| Female 1:1 - Female 2:1 | -0.219 | -1.107 | 0.669 | 1 |
| Female 1:1 - Female 3:1 | -0.924 | -1.812 | -0.036 | 0.423 |
| Male 1:1 - Male 2:1 | -0.824 | -1.638 | -0.010 | 0.46 |
| **Male 1:1 - Male 3:1** | **-1.596** | **-2.410** | **-0.782** | **0.003** |
| Female 2:1 - Male 2:1 | -0.205 | -1.361 | 0.951 | 1 |
| Female 2:1 - Female 3:1 | -0.705 | -1.593 | 0.183 | 0.75 |
| Male 2:1 - Male 3:1 | -0.772 | -1.586 | 0.042 | 0.546 |
| Female 3:1 - Male 3:1 | -0.273 | -1.428 | 0.883 | 1 |
